# Vibronic structure of photosynthetic pigments probed by polarized two-dimensional electronic spectroscopy and *ab initio* calculations†
†Electronic supplementary information (ESI) available. See DOI: 10.1039/c9sc02329a


**DOI:** 10.1039/c9sc02329a

**Published:** 2019-07-03

**Authors:** Yin Song, Alexander Schubert, Elizabeth Maret, Ryan K. Burdick, Barry D. Dunietz, Eitan Geva, Jennifer P. Ogilvie

**Affiliations:** a Department of Physics , University of Michigan , 450 Church St , Ann Arbor , MI 48109 , USA . Email: jogilvie@umich.edu; b Department of Chemistry , University of Michigan , 930 N University Ave , Ann Arbor , MI 48109 , USA; c Department of Chemistry and Biochemistry , Kent State University , 1175 Risman Drive , Kent , OH 44242 , USA; d Applied Physics Program , University of Michigan , 450 Church St , Ann Arbor , MI 48109 , USA

## Abstract

Bacteriochlorophyll a (Bchl a) and chlorophyll a (Chl a) play important roles as light absorbers in photosynthetic antennae and participate in the initial charge-separation steps in photosynthetic reaction centers. Despite decades of study, questions remain about the interplay of electronic and vibrational states within the Q-band and its effect on the photoexcited dynamics. Here we report results of polarized two-dimensional electronic spectroscopic measurements, performed on penta-coordinated Bchl a and Chl a and their interpretation based on state-of-the-art time-dependent density functional theory calculations and vibrational mode analysis for spectral shapes. We find that the Q-band of Bchl a is comprised of two independent bands, that are assigned following the Gouterman model to Q_*x*_ and Q_*y*_ states with orthogonal transition dipole moments. However, we measure the angle to be ∼75°, a finding that is confirmed by *ab initio* calculations. The internal conversion rate constant from Q_*x*_ to Q_*y*_ is found to be 11 ps^–1^. Unlike Bchl a, the Q-band of Chl a contains three distinct peaks with different polarizations. *Ab initio* calculations trace these features back to a spectral overlap between two electronic transitions and their vibrational replicas. The smaller energy gap and the mixing of vibronic states result in faster internal conversion rate constants of 38–50 ps^–1^. We analyze the spectra of penta-coordinated Bchl a and Chl a to highlight the interplay between low-lying vibronic states and their relationship to photoinduced relaxation. Our findings shed new light on the photoexcited dynamics in photosynthetic systems where these chromophores are primary pigments.

## Abstract 2

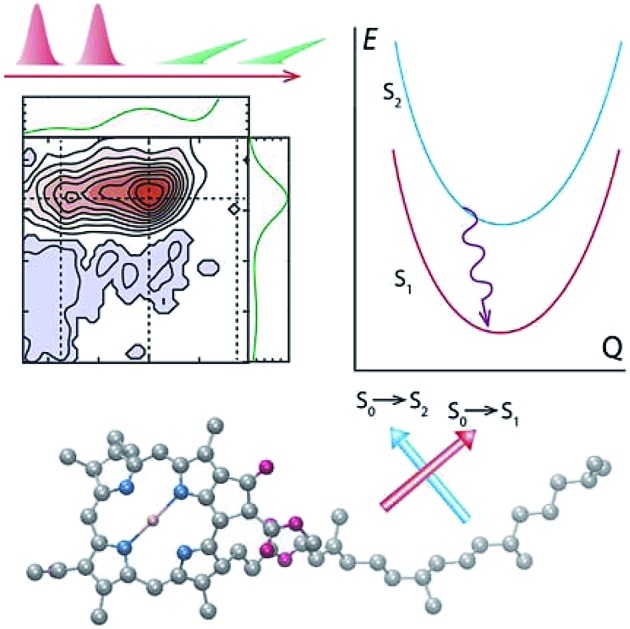
Using polarized 2D spectroscopy and state-of-the-art TDDFT calculations to uncover the vibronic structure of primary photosynthetic pigments and its effect on ultrafast photoexcited dynamics.

## Introduction

**Fig. 1:**
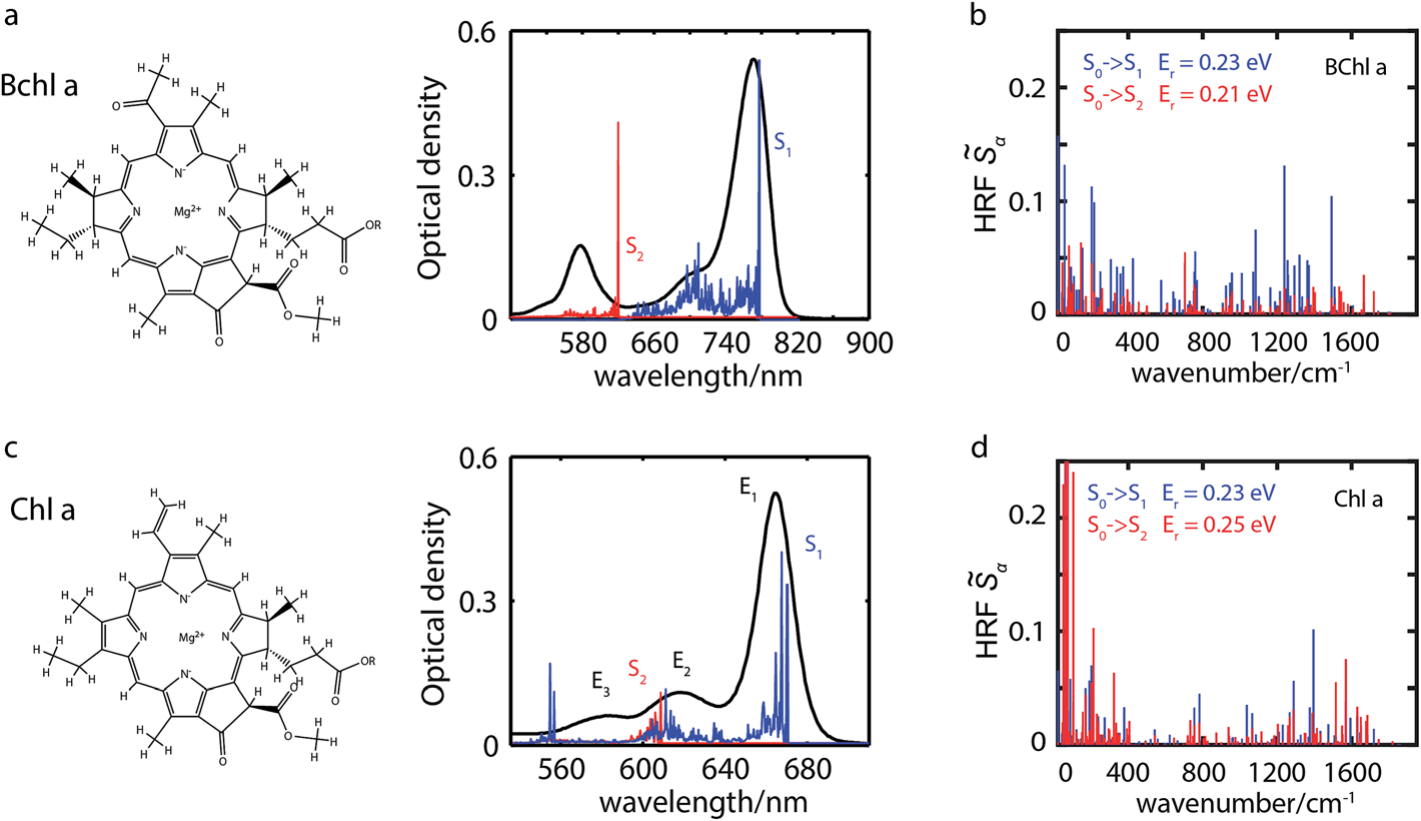
Molecular structures and absorption spectra (experiment: black lines; calculation: red and blue) of penta-coordinated Bchl a in acetone (a) and Chl a in isopropanol (c). Calculated absorption stick spectra of the vibronic S_1_ (blue) and S_2_ (red) excitations are based on Huang–Rhys factors of penta-coordinated Bchl a (b) and Chl a (d), respectively. While low-frequency modes contribute primarily to line broadening, high-frequency modes between 1200 and 1500 cm^–1^ give rise to vibrational replicas of the fundamental line. In Bchl a vibronic excited states are well-separated between the S_1_ and S_2_ excitations, whereas in Chl a a superposition of the S_2_ fundamental line with S_1_ overtones is observed.

Bacteriochlorophyll a (Bchl a) and chlorophyll a (Chl a) are primary pigments found in many photosynthetic systems.^1^–^3^ Both pigments play important roles as light-absorbers in photosynthetic antennae and participate in the initial charge-separation steps in photosynthetic reaction centers. Because of their importance, both Bchl a and Chl a have been widely studied^4^–^36^ to assess their bio-functional roles and to aid in the design of artificial light-harvesting systems. Of particular interest has been the Q-band absorption that is involved in the energy/charge-transfer processes. The Q-band absorption is described well by the four-orbital Gouterman model.^4^,^7^ In this model, following Hückel theory and configuration interaction and accounting for the symmetry of the porphyrin molecule, the lowest unoccupied orbital, e_g_ (LUMO), is twofold degenerate, and the two highest occupied molecular orbitals, a_1u_ (HOMO) and a_2u_ (HOMO–1), are nearly degenerate. Strong interactions between related transitions give rise to the lower energetic Q band and the higher energetic B band, with transition dipole moments (TDMs) within the molecular plane. Symmetry breaking through partial hydrogenation as in Chl a lifts the degeneracy resulting in a splitting of the Q band into two perpendicularly polarized transitions, the blue-shifted Q_*x*_ and the red-shifted Q_*y*_. Further hydrogenation as in Bchl a enhances these trends.^37^ See illustration of these pigments in Fig. 1.

While the relatively simple Gouterman model can explain the spectra qualitatively, a more comprehensive treatment is required for a quantitative understanding of spectroscopic studies.^9^,^12^,^25^,^29^,^38^–^42^ For example, spectroscopic measurements showed that the polarizations of the Q_*x*_ and Q_*y*_ transitions are not orthogonal.^8^,^9^,^11^,^36^,^41^,^42^ The model also fails to quantitatively describe the magnetic circular dichroism (MCD) spectrum.^12^,^29^,^39^,^40^ A number of studies^9^,^25^,^29^,^35^,^36^,^38^,^43^ have proposed that vibronic couplings are responsible for such shortcomings of the model.

To resolve these controversies, we use polarized two-dimensional electronic spectroscopy (P-2DES) to investigate the electronic structure and photoexcited dynamics underlying the Q-band of penta-coordinated Bchl a and Chl a. (P-)2DES is particularly suited for this aim and its capability has been demonstrated in many systems including photosynthetic complexes,^44^–^48^ organic photovoltaic materials^49^,^50^ and quantum dots.^51^–^53^ P-2DES has proven to be a sensitive tool for discriminating between different transitions based on their polarizations.^54^–^58^ P-2DES also has the advantage over steady-state polarization spectroscopy since it does not require that samples be studied at low temperatures or in viscous solvents. Thus, this approach enables us to study penta-coordinated Bchl a and Chl a, both of which tend to become hexa-coordinated at low temperatures.^13^,^25^,^29^,^38^,^59^ Although relatively rarely studied, penta-coordinated Bchl a and Chl a play important roles in many photosystems.^59^

As spectral features are heavily congested particularly in Chl a, we turn to *ab initio* calculations to aid in the interpretation of our experimental results. To account for solvent effects on electronically excited states which have been observed in various studies,^29^,^60^,^61^ we employ a recently developed dielectric screening approach based on screened range-separated hybrid (SRSH) functionals and the polarizable continuum model (PCM). This SRSH-PCM approach has been shown to provide quantitatively more accurate orbital energies in the condensed phase than those by simpler RSH-PCM combinations.^62^ Recent benchmarking of using SRSH-PCM in time-dependent density functional theory (TDDFT) calculations of excited states also presented success in addressing condensed phase effects,^63^ in particular for the study of related pigments.^64^

To elucidate the origin of spectral differences and to quantify the limitations of the Gouterman model, we calculate the excited states in both penta- and hexa-coordinated compounds. Through combined studies of P-2DES with TDDFT, a deeper understanding of the electronic structure and relaxation mechanisms underlying the Q band in both Bchl a and Chl a is obtained.

## Results and discussion

### Absorption spectra of Bchl a and Chl a

The experimental and simulated absorption spectra of penta-coordinated Bchl a and Chl a are displayed in Fig. 1a and c, respectively. The absorption spectrum of Bchl a exhibits two well-separated bands with peak positions at 578 nm and 770 nm. According to the Gouterman model,^4^,^65^ these two bands are assigned to the Q_*x*_ and Q_*y*_ transitions, respectively. The absorption spectrum of Chl a has a dominant peak at 665 nm and two shoulders at 620 and 588 nm. The peak at 665 nm is assigned to the Q_*y*_ transition according to previous studies.^4^,^29^ However, the assignment of the Q_*x*_ peak is controversial.^5^,^7^,^12^,^29^,^38^,^66^ It has been proposed to be either of the shorter wavelength peaks in different models.^4^,^7^,^25^,^29^,^35^ Because of the dilemma in the peak assignments of Chl a, we will use *E*_*n*_ to represent the transition from the ground electronic state to the electronic or vibronic state causing the *n*th observed spectral peak. Calculated adiabatic electronic states are labeled consecutively S_*i*_, where S_0_ represents the electronic ground state.

### Calculated electronic excitation energies

We calculate electronically excited state energies of Bchl a and Chl a with a varying number of ligands. Results obtained from the RSH functional ωB97X-D and the SRSH-PCM approach based on the ωPBE functional are listed in Table 1. The calculations for both functionals are performed using the same molecular geometries obtained from ωB97X-D-based optimization. B3LYP-based structures are discussed in Section S8 and S9 of the ESI† for comparison. In both cases, vertical excitation energies are overestimated with respect to the experimental values of the fundamental line. The overestimation might be related to the vibrational reorganization energy. The SRSH-PCM-based minimum-to-minimum energy differences between ground and excited states show excellent agreement with experiment for penta-coordinated Bchl a with 1.64 eV (S_1_) and 2.04 eV (S_2_) and reasonable agreement for Chl a with 2.00 eV (S_1_) and 2.19 eV (S_2_), respectively. Excitation energy gaps, Δ*E*, are in both cases in very good agreement with experimental values and confirm the suggested peak assignment. The *E*_2_ – *E*_1_ energy gaps of 0.54 eV in Bchl a, Fig. 1a, and of 0.14 eV in Chl a, Fig. 1c, are well reproduced by the calculated S_2_–S_1_ values of 0.40 eV and 0.17 eV, respectively. The ωB97X-D calculations, on the other hand, predict a larger energy gap in Chl a than in Bchl a. These findings illustrate the superiority of the SRSH-PCM approach over the unscreened RSH-PCM alternative. We will therefore restrict further analysis to the SRSH-PCM results.

**Table 1:** Calculated electronic excitation energies and experimental absorption energies for the tetra-, penta-, and hexa-coordinated Chl a and Bchl a, respectively. Absorption energies of the hexa-coordinated Chl a (in pyridine) are taken from ref. 25, and of Bchl a (in dimethylformamide) from ref. 59

Coordination		RSH-PCM ωB97X-D			SRSH-PCM ωPBE			Experiment	
		4	5	6	4	5	6	5	6
Chl a	S_1_ [eV]	2.12	2.11	2.11	2.15	2.13	2.14	1.86 (665 nm)	1.85 (ref. 25) (671 nm)
	S_2_ [eV]	2.57	2.52	2.45	2.36	2.30	2.23	2.00 (620 nm)	1.93 (ref. 25) (640 nm)
	Δ*E* [eV]	0.45	0.41	0.34	0.21	0.17	0.09	0.14	0.08
Bchl a	S_1_ [eV]	2.08	2.07	2.15	1.83	1.84	1.87	1.61 (770 nm)	1.61 (ref. 59) (771 nm)
	S_2_ [eV]	2.37	2.29	2.26	2.31	2.24	2.19	2.15 (578 nm)	2.03 (ref. 59) (610 nm)
	Δ*E* [eV]	0.29	0.22	0.11	0.48	0.40	0.32	0.54	0.42

**Fig. 2:**
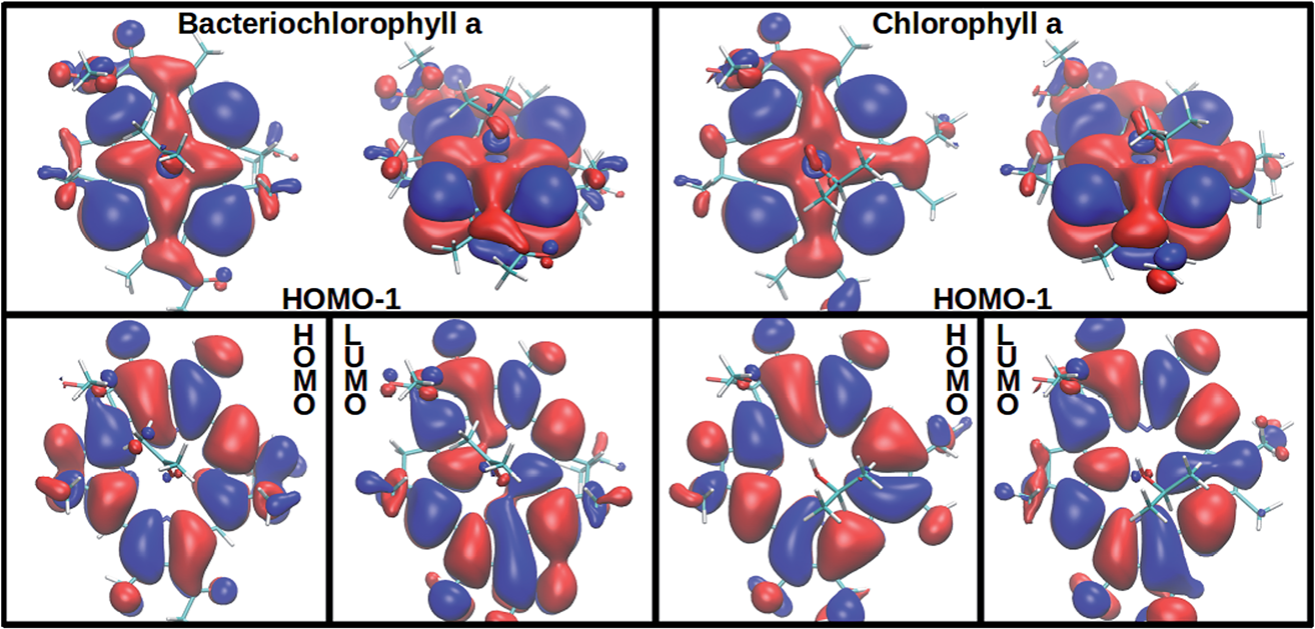
Among the three frontier orbitals forming the S_1_ (HOMO → LUMO) and the s_2_ (HOMO–1 → LUMO) state of the penta-coordinated compounds, Bchl a (left) and Chl a (right), only the HOMO–1 shows significant electron density at the central Mg ion. Its destabilization through ligation thus reduces the S_2_ excitation energy (see Table 1).

In agreement with experimentally observed trends,^60^ the increased coordination as reported in Table 1 is found to decrease the S_2_ excitation energy. This trend can be traced back to the underlying molecular orbitals. In both molecules, the S_1_ state is formed by a HOMO–LUMO transition with a coefficient of more than 0.95. The S_2_ state consists primarily of a (HOMO–1)–LUMO transition (>0.90). The dominant orbital transitions of the S_1_ and S_2_ states correspond to the ones constituting the Q_*y*_ and Q_*x*_ excitation according to the Gouterman model.^4^,^7^ We will therefore associate the S_1_ (S_2_) state with Q_*y*_ (Q_*x*_) in the following. Fig. 2 shows the three relevant orbitals of the penta-coordinated Bchl a (left) and Chl a (right). Importantly, only the HOMO–1 accumulates significant electron density in the immediate proximity of the central Mg ion. Its energy is thus significantly more destabilized by ligation than the HOMO and LUMO energies. Orbitals and orbital energies are listed in Fig. S5–S10 and Tables S2–S5 in the ESI.†

### Vibrational structure

**Fig. 3:**
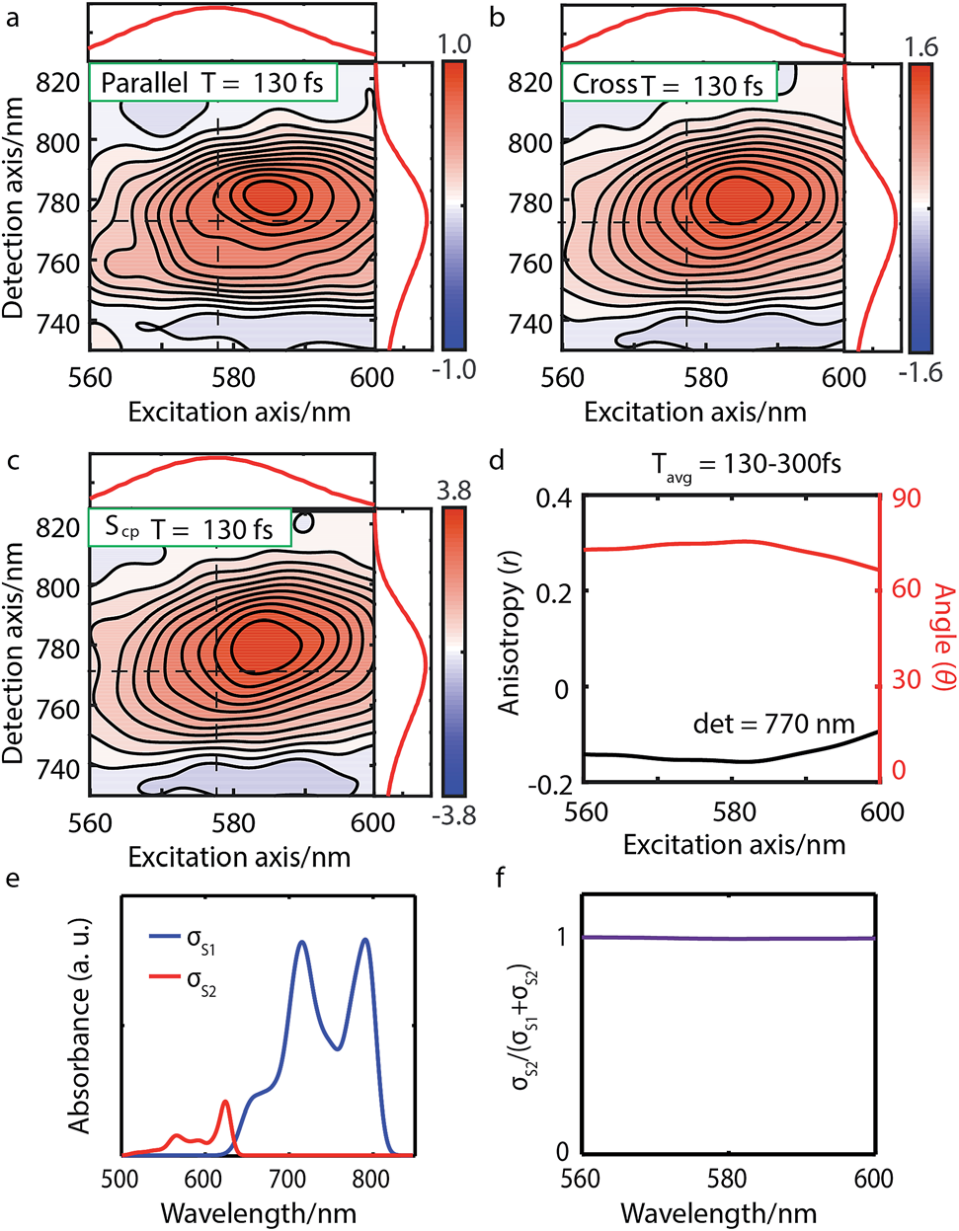
Parallel-polarized (a), cross-polarized (b), and cross-peak specific 2DES absorptive spectra (c) of Bchl a at *t* = 130 fs (contour interval = 0.1). Dashed lines and the absorption spectra alongside 2DES are used to illustrate the peak positions. (d) The spectral cut of anisotropy *r* along the excitation axis with detection wavelength at 770 nm and the calculated angle *θ* between the Q_*x*_ and Q_*y*_ TDMs. (e) Calculated linear absorption spectra *σ*_S_1__, *σ*_S_2__ based on eqn (1), broadened by a 40 meV Gaussian function. (f) The S_2_ contribution *σ*_S_2__ to the total intensity *σ*_S_1__ + *σ*_S_2__ shows no overlap between S_1_ and S_2_ excitations and thus no wavelength dependence.

**Fig. 4:**
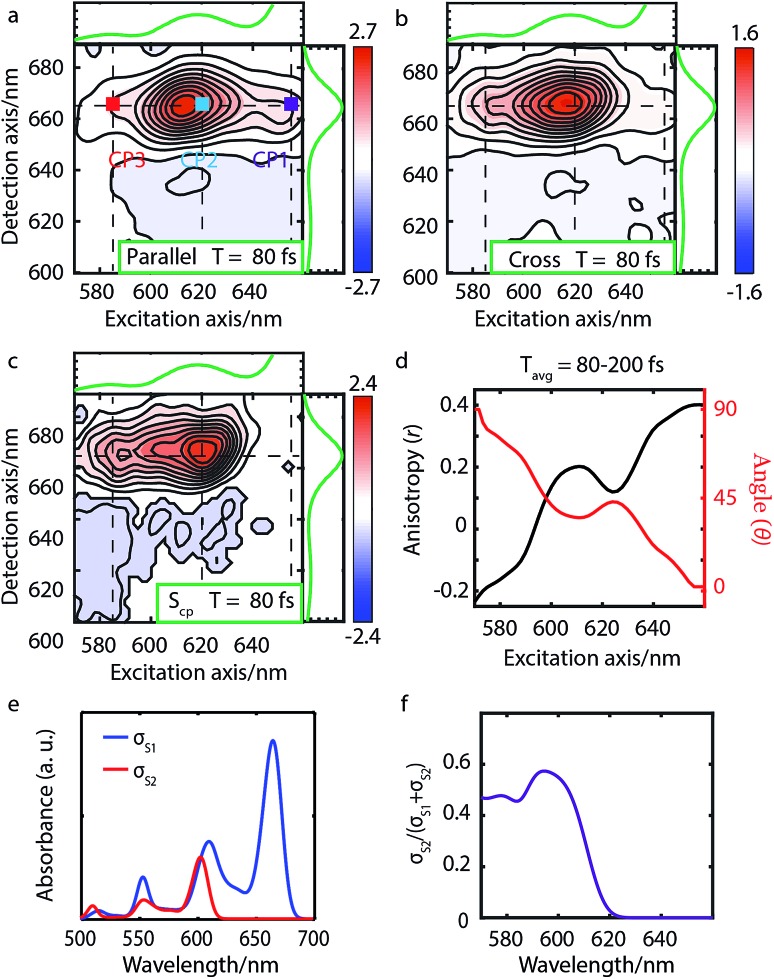
Parallel-polarized (a), cross-polarized (b) polarized and cross-peak specific (c) 2DES absorptive spectra of Chl a at 80 fs (contour interval = 0.1). Dashed lines and the absorption spectra alongside 2DES are used to illustrate the peak positions. (d) The anisotropy *r* and the angle *θ* calculated from the measured spectra *S*_p_ and *S*_c_. (e) The calculated linear absorption spectra *σ*_S_1__ and *σ*_S_2__ based on eqn (1), broadened by a 40 meV Gaussian function, show an overlap between the S_2_ fundamental line and the S_1_ vibrational replica. (f) The S_2_ contribution *σ*_S_2__ to the total intensity *σ*_S_1__ + *σ*_S_2__ shows the same wavelength dependence as the calculated angle *θ* in panel d.

To elucidate the role of nuclear degrees of freedom, vibrational normal modes are calculated at the optimized ground-state geometry of the penta-coordinated molecules. Displacement vectors to geometries of excited state minima are projected onto the set of normal modes to obtain the Huang–Rhys factors^67^,^68^ (HRFs) *S[combining tilde]*_*α*_^*i*^, which are shown in Fig. 1b and d. For the first excited Bchl a state, a particularly large HRF is found at 1239 cm^–1^, corresponding to 0.15 eV (see Fig. 1c, blue lines). This supports the interpretation of the shoulder at ∼700 nm in Fig. 1a as the first vibrational replica of the S_1_ (Q_*y*_) excitation. The same mode is activated in the S_2_ (Q_*x*_) excitation alongside higher frequency modes (red lines). However, the HRFs are about 5 times smaller and the shoulder is thus less prominent in the Q_*x*_ absorption band. In Chl a (Fig. 1d), large HRFs for both excitations, S_1_ and S_2_, are found at 1395 cm^–1^ (0.17 eV) and 1568 cm^–1^ (0.19 eV), respectively, which is close to the S_2_–S_1_ excitation energy gap. Neglecting a potential mixing of electronic states (see discussion below), these findings indicate that the first vibrational replica of the Q_*y*_ excitation, Q_*y*1_, overlaps with the Q_*x*_ fundamental line, Q_*x*0_, resulting in the *E*_2_ absorption line. Consequently, the second overtone Q_*y*2_ overlaps with the first overtone Q_*x*1_, giving rise to the third absorption line *E*_3_ (see Fig. 1c). While these assignments are energetically in excellent agreement with spectra of both compounds, Bchl a and Chl a, the relative intensities between fundamental lines and overtones are not accurately described by a single active mode. In particular for Chl a, the observed high intensity of the *E*_3_ line relative to the *E*_2_ signal cannot be explained by the slightly smaller HRF found in the S_2_ state. To investigate if this deviation could be due to the remaining modes, spectra *σ*_*i*_(*E*) (Fig. 1a and c) were calculated as follows:^69^1
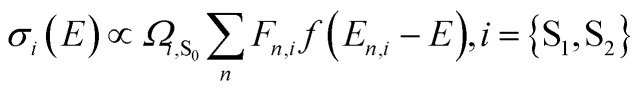
where the oscillator strengths, *Ω*_S_1_,S_0__ = 0.47, *Ω*_S_2_,S_0__ = 0.18 for Bchl a and *Ω*_S_1_,S_0__ = 0.39, *Ω*_S_2_,S_0__ = 0.07 for Chl a, are assumed to be constant within the Condon approximation. The Franck–Condon factors, 
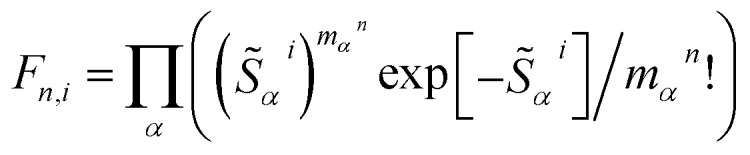
, are determined from the HRFs of all vibrational modes and their occupancy *m*_*α*_^*n*^ in the *n*th vibronic state. States with excitation energies 
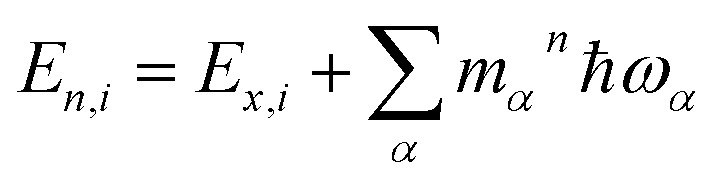
 of up to two vibrational energy quanta
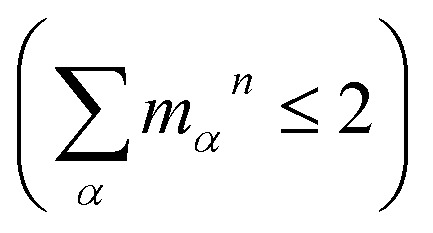
 are considered. Energy differences are calculated between the minima of the electronic ground state and the excited states: *E*_*x*,S_1__ = 1.64 eV and *E*_*x*,S_2__ = 2.04 eV in Bchl a and *E*_*x*,S_1__ = 2.00 eV and *E*_*x*,S_2__ = 2.18 eV for Chl a. The absorption spectra are reproduced using a Gaussian function (*f*(*E*)) with a full width at half maximum energy of *ε*_FWHM_ = 1 meV giving rise to the spectra in Fig. 1a and c. The spectra are normalized to the *E*_1_ peak intensity and shifted by 0.045 eV for Bchl a and by 0.150 eV for Chl a to account for the overestimation of calculated excitation energies. The relative intensities between the Q_*y*_ and Q_*x*_ fundamental lines and between these lines and higher vibrational states are in good agreement with the absorption spectra. However, if broadening effects are considered (*ε*_FWHM_ = 40 meV, see in Fig. 3e and 4e), the vibrational replicas obtain significantly higher intensities than observed in the experiment. This deviation cannot be explained by a potential overestimation of reorganization energies *E*_r_, whose correction would yield insufficient intensity in the *E*_3_ peak. We therefore expect either a violation of underlying assumptions, such as the harmonic approximation and the Condon approximation, or vibronic coupling between electronic states altering the predicted intensities.

### Polarized-2DES and anisotropy

We performed 2DES measurements with two polarization schemes – p-polarized pump, p-polarized probe (*S*_p_) and s-polarized pump, p-polarized probe (*S*_c_). The corresponding 2DES absorptive spectra of the Bchl a at 130 fs are shown in Fig. 3a and b. Both spectra exhibit one broadband cross peak with excitation wavelength at 578 nm and detection wavelength at 780 nm. The peak position is slightly shifted from the absorption spectrum owing to the amplitude modulation induced by the laser pulse spectra. The presence of the cross peak suggests that the Q_*x*_ and Q_*y*_ transitions share a common ground state.

To verify that the Q_*x*_ and Q_*y*_ transitions have different polarizations, we calculated the so-called ‘cross-peak specific spectrum’^55^,^57^ using the formula *S*_cp_ = 3*S*_c_ – *S*_p_, which removes the signals from pathways involving only parallel transition dipole moments (TDMs) and highlights ones from pathways with non-parallel TDMs. The *S*_cp_ spectrum at 130 fs is displayed in Fig. 3c. *S*_cp_ exhibits a cross peak at the same position as the parallel- and cross-polarized 2DES. This observation is qualitatively consistent with the Gouterman model^4^ which predicts that these two transitions are perpendicularly polarized. To quantitatively evaluate the polarizations of the TDMs, we calculated anisotropy (*r*) and the angle (*θ*) between the *E*_1_ and *E*_2_ transitions using the following formula:^54^,^70^
2
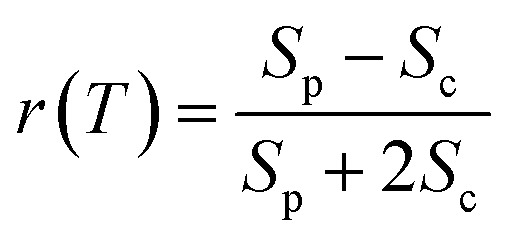

3
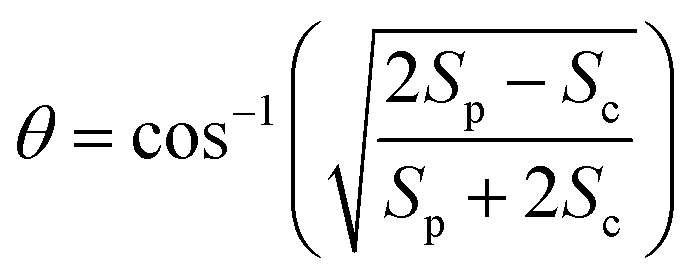



We note potential interferences of ground state bleaching (GSB), stimulated emission (SE) and excited-state absorption (ESA) may alter the interpretation of anisotropy and angle calculation. However, this is not the case in the current study for several reasons. First, after internal conversion has completed, GSB and SE signals at the cross peak come from the same transition dipole moment (*i.e.* Q_*y*_) and would have the same anisotropy under the Condon approximation. This argument is supported by the observation that the anisotropy remains almost constant from 125–500 fs (see Fig. S4a†). We also note that previous publications^10^,^71^ have claimed that ESA might be hidden underneath the GSB signals and can potentially change the anisotropy.^19^ However, two groups^18^,^72^ measured the anisotropy at the Q_*y*_ peak upon excitation of the Q_*y*_ transition and respectively reported a value of about 0.4. These results suggest that either the extinction coefficient of ESA is small or that the ESA transition has the same polarization as Q_*y*_. In both cases, eqn (3) is valid to estimate the angle between Q_*x*_ and Q_*y*_. This argument is verified in ESI Section 5† by performing the angle calculation at the condition where a weak ESA is considered. In addition, molecular rotational dynamics occurs on the picosecond timescale and can therefore be neglected in the following analysis. However, we want to stress that the TDM orientation can in principle change on shorter timescales through photo-induced nuclear reorganization. Experimental and theoretical investigations of such non-Condon effects in Bchl a and Chl a are currently underway in our groups and will be published elsewhere.

Since the anisotropic signal is relatively weak and remains unchanged after internal conversion, the spectrum averaged over *T* = 130–300 fs is used in the calculation. A spectral cut of the *θ* and *r* results along the excitation axis is shown in Fig. 3d. We find that the anisotropy shows an almost constant value of ∼–0.15 through the whole excitation band, which confirms that the Q-band is composed of only two electronic transitions. Using eqn (3) we find the angle between the Q_*x*_ and Q_*y*_ TDMs to be ∼75°. This result is in excellent agreement with the SRSH-PCM TDDFT calculations which yield an angle of 77.6° between the S_1_ and S_2_ TDMs. To the best of our knowledge, no earlier experimental studies have been reported to evaluate the angle between the Q_*x*_ and Q_*y*_ transitions for penta-coordinated Bchl a. However, some relevant studies^5^,^11^,^73^ support our finding that the Q_*x*_ and Q_*y*_ angle can deviate from the 90° angle expected from the Gouterman model. For example, the Goedheer^5^ and Ebrey^11^ groups, respectively measured polarized fluorescence excitation spectra of Bchl a with unknown coordination status in cyclohexanol and castor oil, and found the angle between Q_*x*_ and Q_*y*_ to be ∼68–72°. Christoffersen *et al.*^73^ performed semi-empirical calculations on Bchl a and reported an angle of ∼70° between the Q_*x*_ and Q_*y*_ transition. Thus, our measurement of a 75° angle between the Q_*x*_ and Q_*y*_ TDMs for penta-coordinated Bchl a and our calculated value of 77.6° are reasonably consistent with previous reports.^5^,^11^,^73^

We present the parallel-polarized, cross-polarized and cross-peak specific 2DES absorptive spectra of Chl a at 80 fs in Fig. 4a–c, respectively. The former two 2D spectra show three peaks with excitation wavelength at 650 (CP1), 620 (CP2) and 588 (CP3) nm and detection wavelength at 665 nm. However, only CP2 and CP3 remain in the *S*_cp_ spectra, suggesting that CP1 is the vibrational overtone of the *E*_1_ transition while CP2 and CP3 may originate from different electronic/vibronic transitions.

To elucidate the peak origins of CP2 and CP3, we evaluated anisotropy *r* and the angles *θ* of the excitation bands with respect to the *E*_1_ transition. Similar to Bchl a, the anisotropy remains constant after 80 fs when internal conversion is complete (see Fig. S4c†). To calculate the angles, we considered the potential interference between GSB, SE and ESA at the cross peaks. As discussed above, GSB and SE have the same anisotropy after internal conversion. Several measurements^15^,^17^,^74^ including transient absorption and Z-scan spectroscopy have shown that the extinction coefficient of ESA at the Q_*y*_ peak is less than 5%, making eqn (3) valid for estimating the angle between TDMs (see S5 and Fig. S4d†). A spectral cut along the excitation axis with detection wavelength at 665 nm is shown in Fig. 4d. As with Bchl a, we employ a time-averaged spectrum from 80–200 fs for the measurement because of the low signal amplitude. Unlike Bchl a, we find that both the anisotropy and angles exhibit a strong wavelength dependence. At CP2 and CP3 peaks, the angles of the TDMs with respect to the *E*_1_ transition are found to be ∼45° and 60°, respectively.

The wavelength-dependent anisotropy in the Q-band has also been reported in previous studies.^8^,^9^,^19^,^42^ Polarized fluorescence excitation spectra^9^,^42^ showed that for hexa-coordinated Chl a, the angle between the *E*_1_ and *E*_2_ signal is ∼56°. By using linear dichroism and polarized fluorescence spectra, Bauman *et al.*^8^ reported that the angle between the *E*_1_ and *E*_3_ polarization is ∼90° for Chl a with an unknown coordination status in a liquid crystal. Lin *et al.*^19^ measured pump-probe anisotropy of hexa-coordinated Chl a in ethanol under excitation at 580, 620, 660 nm and showed that the anisotropy values have a strong excitation wavelength dependence. All these results suggest that the polarizations in the Q-band have strong wavelength dependence. However, to the best of our knowledge, no anisotropy spectrum for the whole Q-band of penta-coordinated Chl a has been reported. Here, we present a complete anisotropic map, providing a benchmark for theoretical simulations.

To interpret the polarization behavior in the Q band of Chl a, we turn to TDDFT calculations. The calculated angles between adiabatic electronic states are 79.7° for tetra-, 75.0° for the penta-, and 73.8° for the hexa-coordinated Chl a, respectively. The dependence on the degree of axial ligation, which is not present in Bchl a, can be traced back to a stronger contribution of the LUMO+1 to the S_2_ state in Chl a (∼0.4) than in Bchl a (∼0.2), which is not considered to contribute to the Q band in the Gouterman model. However, the calculated angle of 75° for penta-coordinated Chl a deviates significantly from the *θ* values obtained from experiment (*i.e.*, 45° for CP2 and 60° for CP3). We ascribe the smaller angles to varying degrees of the overlap of S_1_ and S_2_ spectral features. Fig. 4e shows the simulated absorption spectra *σ*_*i*_ for each electronic state, *i* = 1, 2, calculated from HRFs, eqn (1), considering vibronic states of up to two vibrational quanta and a Gaussian line broadening of 40 meV. The blue curve in Fig. 4f reflects the S_2_ contribution to the total signal and is thus a measure of the spectral overlap. At wavelengths around 640 nm (CP1), only vibrational states within the S_1_ electronic state can be found which are of the same polarization as the *E*_1_ transition and therefore vanish in the *S*_cp_ spectrum, resulting in a relative angle of 0°. As intensities of S_2_ transitions appear at shorter wavelengths, *S*_cp_ increases and so does seemingly the angle *θ* evaluated by eqn (3). The wavelength dependence of the spectral overlap (panel f) strongly resembles the one of the angle *θ* (panel d). We therefore interpret *θ* not as relative TDM angle, but as a composition of 0° contributions from the Q_*y*_ transition and an unknown angle ≥60° from the Q_*x*_ contribution, resulting in an effective intermediate value. CP2 can thus be interpreted as a superposition of the Q_*x*0_ fundamental line and the Q_*y*1_ vibrational replica, whereas CP3 stems from the Q_*x*1_ and Q_*y*2_ overtones.

We note that there have been suggestions that vibronic coupling plays an important role in the electronic structure of the Q band of Chl a.^9^,^25^,^29^,^38^ Recently, such vibronic coupling was evaluated in the model calculations by Reimers *et al.*^29^ These authors simulated the absorption and MCD spectra of Chl a and found that no satisfactory fit could be obtained within the Condon approximation without including vibronic coupling. In the limit of intermediate/strong coupling, vibronic coupling mixes electronic transitions and gives rise to a set of vibronic transitions with different polarizations. This alternative picture could also qualitatively explain our experimental observations. Our TDDFT calculations are not in contradiction with the vibronic coupling model since the calculated excited states show a strong nuclear-coordinate dependence.

Our findings that the vibrational replica of the S_0_ → S_1_ transition and the S_0_ → S_2_ transition are degenerate in Chl a suggests that vibronic states can mix *via* weak electronic/vibronic coupling. However, such an effect is weakened in Bchl a because of the large energy gap. This raises interesting questions about the importance of electronic/vibronic coupling for the photoexcited dynamics. Both Bchl a and Chl a pigments play key roles in energy transfer in photosynthetic antennae and charge separation in photosynthetic reaction centers, motivating a detailed understanding of their electronic structure. The Q-bands are of particular importance; in photosynthetic antennae the Q-band states lie at the bottom of the energy funnel that feeds excitation into the reaction centers. Within the reaction centers, the excitation of the Q-band states directly precedes primary charge separation. Despite many studies,^2^,^75^,^76^ debates remain about the site energies, coupling strength among pigments and the delocalization of the excitonic states in both photosynthetic antenna and reaction centers. Previous studies suggest that^44^,^57^,^77^–^79^ excitonic delocalization may play important roles in energy/charge transfer and coherent dynamics. Our measurements that inform about the vibronic structure of Bchl a and Chl a provide important inputs for models of photosynthetic energy transfer and charge separation. In the following, we investigate internal conversion processes and study the way in which electronic/vibronic coupling affects the Q-band structure.

### Internal conversion

**Fig. 5:**
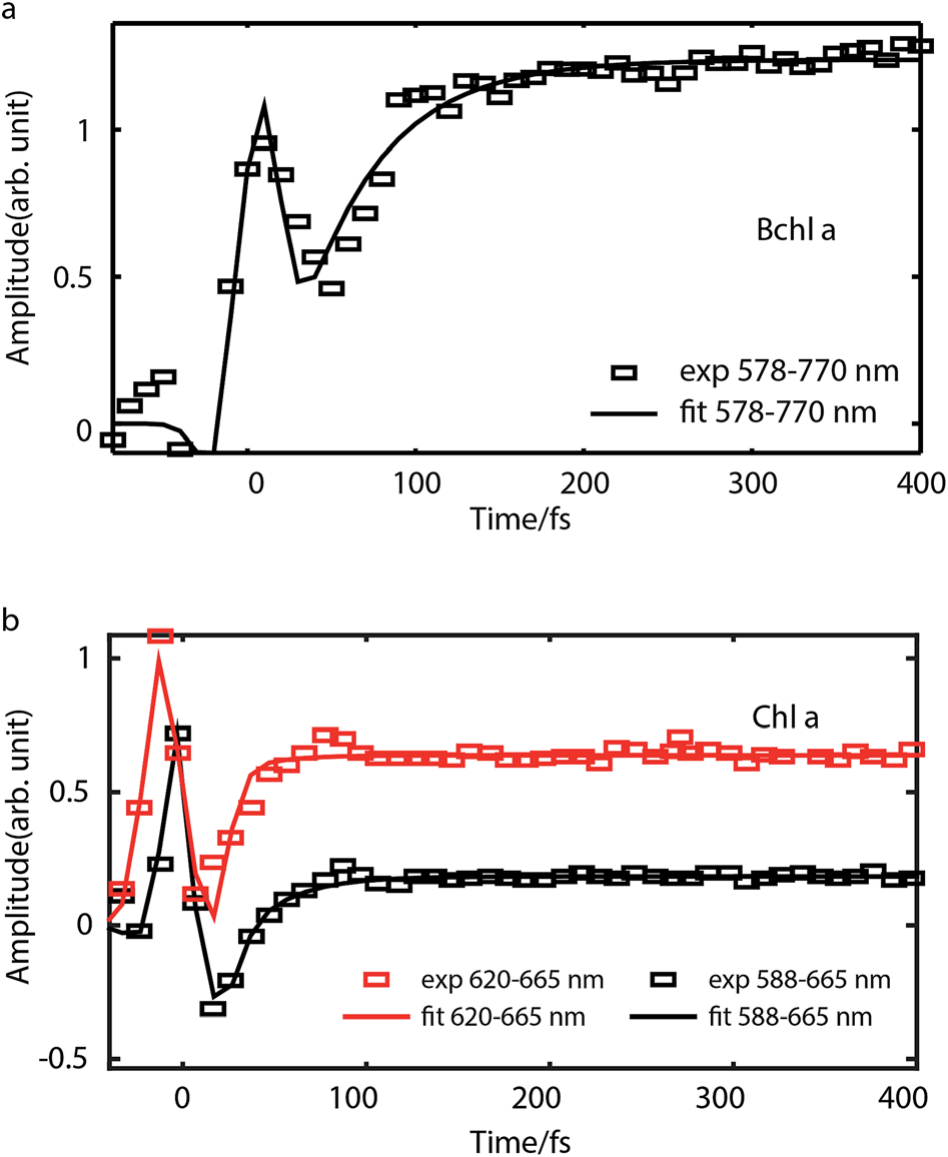
Time traces (scattered plots) and their fits (solid curves) corresponding to internal conversion of Bchl a (a) and Chl a (b) in magic-angle condition. For Bchl a, time trace of cross peak with excitation at 578 nm and detection at 770 nm is plotted. For Chl a, time traces of cross peaks with excitation at 620 nm and 588 nm are shown.

**Fig. 6:**
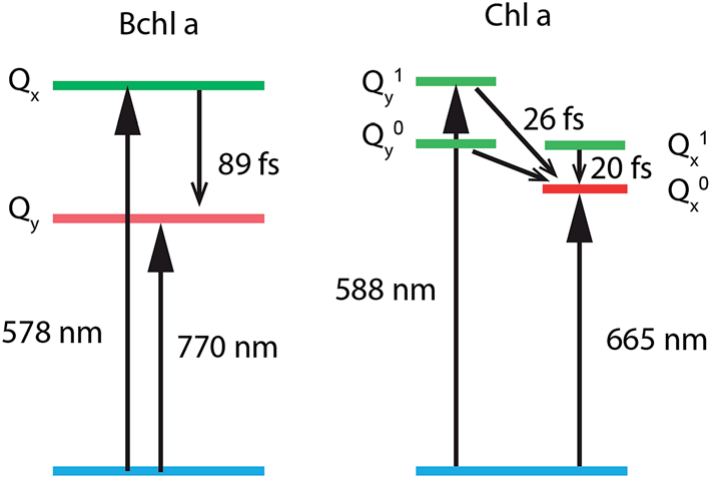
Energy-level diagrams and internal conversion timescales of relevant vibronic states of Bchl a and Chl a.

To study internal conversion, we reconstruct the signal under the magic angle condition using parallel- and cross-polarized spectra. Fig. 5a and b display time traces and the exponential fits of cross peaks for Bchl a and Chl a, respectively. These time traces showed a strong solvent response (or coherent artifact) in the first 50 fs. To account for the coherent artifact and obtain better estimates of the internal-conversion rates, we applied an additional term derived by Ernsting^80^ in the fitting function (as shown in the Section S3 of ESI†). The fitting results are summarized in the Table S1.† We find that for Bchl a, internal conversion from the Q_*x*_ state to the Q_*y*_ state has a rate of 11 ps^–1^, whereas for Chl a, internal conversions from *E*_2_ to *E*_1_ and *E*_3_ to *E*_1_, have rates of 50 ps^–1^ and 38 ps^–1^, respectively (Fig. 6).

Internal conversion in the Q-band of Bchl a and Chl a have been studied previously.^25^,^29^,^38^ Freiberg *et al.* estimated internal conversion rates from the linewidth of absorption spectra and fluorescence line narrowing and found the rates to be ∼30 ps^–1^(or (33 fs)^–1^) for hexa-coordinated Bchl a^29^,^38^ and 31–62 ps^–1^(or (16–32 fs)^–1^) for hexa-coordinated Chl a at 4 K.^25^ The faster rates obtained and the failure to distinguish the rates from two pigments in these experiments may be owing to an underestimation of inhomogeneous broadening. Pump-probe spectroscopy showed^19^,^20^,^27^,^31^,^81^ that for both Bchl a and Chl a, internal conversion occurs within 100 fs, which reaches the time-resolution limit of these measurements. Recently, high time-resolution 2DES have been performed to study the photoexcited dynamics of hexa-coordinated Chl a.^28^,^32^,^34^ However, these measurements primarily focused on the dynamics within the Q_*y*_ band. Collini *et al.*^34^ recorded rephasing spectra of hexa-coordinated Chl a using parallel-polarized pump and probe pulses with spectra spanning from 625–690 nm and observed a 170 fs component attributed to vibrational relaxation within the S_1_ band. They further suggested that internal conversion from S_2_ to the high-lying vibrational state of S_1_ can be as fast as 40 fs. Here we have employed polarization-dependent 2DES over a broad spectral range to obtain internal conversion rates of both Bchl a and Chl a. Our observation of fast internal conversion rates in Chl a is also consistent with Collini's work.

To estimate the effective electronic coupling strength, we use a fully quantum-mechanical Fermi's Golden Rule rate expression for internal conversion and substitute the rate by the inverse of the measured lifetime *τ*_IC_:^68^,^82^,^83^
4
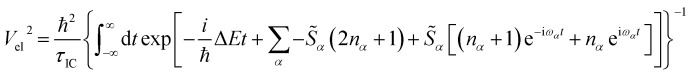
where Δ*E* is the difference between the optimized potential energies of the S_1_ and S_2_ state (Bchl a: 0.40 eV, Chl a: 0.17 eV), {*S[combining tilde]*_*α*_} are the HRFs for the S_1_ → S_2_ displacement, and *n*_*α*_ is the phonon density at room temperature for a vibrational mode with frequency *ω*_*α*_. We found the effective electronic coupling values to be 53 meV in Bchl a and 45 meV in Chl a, respectively, corresponding to the weak-to-intermediate coupling regime.^84^ However, such weak coupling is strong enough to mix the vibronic states in Chl a where the vibronic replica of Q_*y*_ is degenerate with Q_*x*_ during internal conversion. Recently, Reimers *et al.* proposed that similar mixing of Q_*x*_ and Q_*y*_ transitions can also be induced by vibronic coupling^29^ and plays an important role during internal conversion. Further experiments are required to quantitatively determine the effect of Q_*x*_ and Q_*y*_ mixing on the photoexcited dynamics.

## Experimental

### Sample preparation

Chl a from spinach and Bchl a from *Rhodopseudomonas sphaeroides*, isopropanol (≥99.99%) and acetone (≥99.99%) with HPLC Plus grade were purchased from Sigma Aldrich and used as received. The solvents were purged with N_2_ gas for 5 minutes before use. Chl a isopropanol solutions and Bchl a acetone solutions were prepared under N_2_ atmosphere and solutions were stored and sealed in a 200 μm pathlength cuvette for the spectroscopic measurements. Cuvettes were sealed with vacuum grease or epoxy. The absorption spectra of samples were measured before and immediately after ultrafast spectroscopic measurements and no photodegradation was observed.

### Spectroscopic measurements

2DES spectra were measured by using a hybrid diffractive-optics and pulse shaper setup as described previously.^47^,^85^,^86^ Briefly, a regenerative amplifier (Spectra Physics Spitfire Pro) seeded by a Ti:sapphire oscillator (MaiTai SP from Spectra Physics) is used as the laser source. The 4 mJ, 800 nm, 40 fs, 500 Hz output from the amplifier is split and feeds two home-built two-stage non-collinear optical parametric amplifiers (NOPAs)^87^ and one collinear optical parametric amplifier (OPA).^88^ One NOPA is used as the pump beam and tuned to excite the Q_*x*_ band of either Bchl a or Chl a. The other NOPA and OPA is used as the probe for Chl a and Bchl a, respectively. The pump beam is sent through a precompensating grism and then into an acousto-optic pulse shaper (Dazzler, Fastlite) where a compressed pulse pair with a programmable time delay (*t*_1_) is generated. The probe beam from the NOPA is compressed by another grism. The pump and probe NOPA are compressed to 13 and 15 fs using the SPEAR method^89^ and MIIPS,^90^ respectively. The NOPA probe pulse duration is estimated by fitting the coherent artifact from transient grating measurements of cresyl violet. The OPA probe pulse is sent to a commercial liquid crystal spatial phase modulator (femtoJock from Biophotonics solution, Inc) and compressed to 10 fs. The pump and probe pulses are directed to a diffractive-optic imaging system to generate the third-order 2DES signal, which is detected by a CCD camera (Princeton instrument). During the experiments, *t*_1_ is scanned using the Dazzler from 0 to 400 fs with time steps of 10 fs. The pump-probe delay (*T*) is controlled by an optical delay line (DDS220, Thorlabs Inc.) and scanned from –60 to 500 fs. A six phase-cycling scheme is used as described previously to remove scattering and background signals.^85^ A shutter added in the probe arm removed residual scattering from the pump. In the experiments, the pulse energy of pump and probe pulses were ∼20 nJ and 12 nJ, respectively and the beam waists for both pump and probe were ∼200 μm. To control the polarizations, a waveplate and a wiregrid polarizer (Thorlabs, Inc.) are used in the pump arm between the Dazzler and the diffractive optic. A wiregrid polarizer, a waveplate and another wiregrid polarizer are added in sequence in the probe arm. The parallel- and cross-polarized 2DES are collected by changing the polarization of the pump beam. We performed tests and verified that there was no spectral shift of the pump beams when changing the polarizations. The pump power during the parallel- and cross-polarized 2DES is adjusted to be constant at the sample position by using the combination of the waveplate and the polarizer. The data are analyzed using home-written Matlab scripts. All experiments have been performed for at least three times to ensure reproducibility.

### Calculations

Electronic structure calculations were performed with the Q-Chem software package, version 4.4,^91^ using our novel TDDFT framework combined within a PCM. Unless explicitly stated otherwise, the conductor-like polarizable continuum model (C-PCM)^92^–^94^ was employed throughout this analysis, simulating the isopropanol (static dielectric constant *ε*_0_ = 20.18, optical dielectric constant *ε*_∞_ = 1.90) and acetone (*ε*_0_ = 21.01, *ε*_∞_ = 1.85 (ref. 95)) solvents of Chl a and Bchl a, respectively. For both molecules the phytyl-containing side groups were removed to reduce computational costs, since they have no impact on spectral properties.^61^,^96^,^97^ Either zero, one, or two solvent molecules were added explicitly, giving rise to an unligated (tetra-), monoaxially ligated (penta-), or biaxially ligated (hexa-coordinated) central Mg ion, respectively.^98^,^99^ The split-valence double-zeta basis set 6-31++G(d,p)^100^ was used for all calculations.

We used the dispersion-corrected range-separated hybrid^101^ (RSH) functional ωB97X-D for geometry optimizations in the electronic ground and excited states and for normal mode calculations. Excitation energies were additionally calculated with the recently developed PCM-optimally-tuned screened range-separated hybrid approach (SRSH-PCM)^62^ using the ωPBE functional. In this approach, the exchange–correlation energy is of the following form^102^

5 *E*SRSHxc = (1 – *α*)*E*SR,GGA,x^*γ*^ + *αE*SR,F,x^*γ*^ + [1 – (*α* + *β*)]*E*LR,GGA,x^*γ*^ + (*α* + *β*)*E*LR,F,x^*γ*^ + *E*_GGA,c_

where SR and LR indicate short and long range components, mixing the exact Fock exchange (F) with the approximate generalized gradient (GGA) exchange (x) and correlation (c) contribution. The parameters *α* and *β* determine the weights of the individual components, and *γ* is the range-separation parameter.^101^ By setting *α* + *β* = 1/*ε*_0_, electron coulombic interactions are effectively screened by 1/*ε*_0_ thereby achieving consistency with the self-consistent reaction field implementing the PCM.

The functional parameters were determined as follows: first, the range separation parameter *γ* was determined in the gas phase (*i.e.* without C-PCM and with *α* + *β* = 1, where *α* = 0.2 as widely employed^102^). In the tuning process differences were minimized between the ionization potential and the HOMO energy of the neutrally charged molecule and of the anion. A similar non-empirical tuning procedure was then employed with PCM, where *α* tuning involves resetting *β* to 1/*ε*_0_ – *α* to ensure the LR screening of the exact exchange by 1/*ε*_0_. At these PCM tuning calculations *γ* was fixed at the isolated molecule value. This procedure achieves consistent treatment of the dielectric screening between the PCM and the DFT calculations and has been found to avoid the problematic collapse of the range-separation parameter in dielectric medium tuning.^103^ The SRSH-PCM was recently shown to compare well with ionization energies measured in thin-film environments.^62^ Very recently we used the same protocol to explain the fine spectral splitting of the central pigments in bacterial reaction center^64^ and to calculate solvated charge-transfer state's energies.^63^ A related approach, where the *α* and *β* tuning was performed without the C-PCM environment, was recently successfully applied to calculate spectral properties.^104^

## Conclusions

To conclude, we performed P-2DES to investigate the electronic structure of the Q-band and its internal conversion processes in penta-coordinated Bchl a and Chl a. We find that the Q-band of Bchl a is composed of distinct Q_*x*_ and Q_*y*_ transitions with an angle of ∼75° and no significant perturbation due to vibronic coupling. Excitation energy differences and relative transition dipole moments were in excellent agreement with TDDFT calculations. The same protocol failed to reproduce relative angles measured in Chl a, where the spectral signals exhibit a strong wavelength dependence. Simulated spectra based on the HRFs indicate that deviations are consistent with a spectral overlap between overtones of the Q_*x*_ and Q_*y*_ transitions. Furthermore, we also took advantage of the high time resolution of 2DES to determine the internal conversion rates in Bchl a and Chl a. We find that Bchl a has a rate of 11 ps^–1^, slower than that of Chl a which was found to be 38–50 ps^–1^. The faster internal conversion in Chl a may stem from the mixing of Q_*x*_ and Q_*y*_ vibronic states and a smaller energy gap than is found in BChl a. Our results shed light on the electronic structure of Bchl a and Chl a, which is important for improving our understanding of the energy transfer and charge separation processes in photosynthetic antennae and reaction centers.

## Conflicts of interest

There are no conflicts to declare.

## Supplementary Material

Supplementary information
