# Supplementary material for: Vibronic structure of photosynthetic pigments probed by polarized two-dimensional electronic spectroscopy and ab initio calculations
Source: Chem Sci. 2019 Jul 3;10(35):8143–53. doi: 10.1039/c9sc02329a (PMC6836992; doi:10.1039/c9sc02329a)
Supplement: Supplementary file 1 [file SC-010-C9SC02329A-s001.pdf]

Electronic Supplementary Material (ESI) for Chemical Science.
This journal is © The Royal Society of Chemistry 2019

Supporting Information
to

Vibronic structure of photosynthetic pigments probed by polarized two-dimensional electronic spectroscopy and ab initio calculations

Yin Song[a†], Alexander Schuber[b,c†‡], Elizabeth Maret[d], Ryan K. Burdick[b], Barry D. Dunietz[c], Eitan Geva[b] and Jennifer P. Ogilvie[a*]

[a] Department of Physics, University of Michigan, 450 Church St, Ann Arbor MI 48109

[b] Department of Chemistry, University of Michigan, 930 N University Ave, Ann Arbor, MI 48109

[c] Department of Chemistry and Biochemistry, Kent State University, 1175 Risman Drive, Kent, OH 44242

[d] Applied Physics Program, University of Michigan, 450 Church St, Ann Arbor MI 48109

[†] These two authors contributed equally.

[‡] Present address: Institute of Physical Chemistry, Friedrich Schiller University Jena, Helmholtzweg 4, 07743 Jena, Germany

*Correspondence: *jogilvie@umich.edu

S1. Pump and probe spectra, along with absorption spectra of Chl a and Bchl a

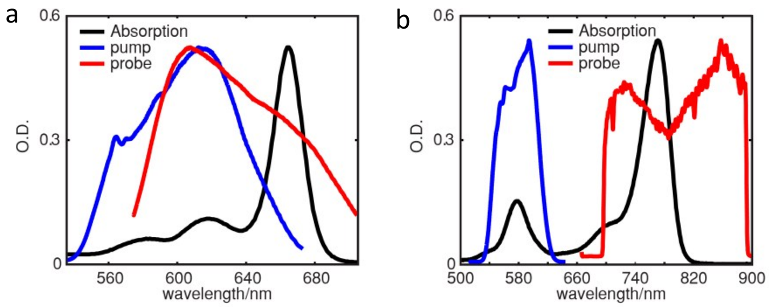

Figure S1 Absorption spectra of Chl a (a) and Bchl a (b), along with pump and probe spectra used in polarized 2DES.

S2. Chirp scan of pump pulse

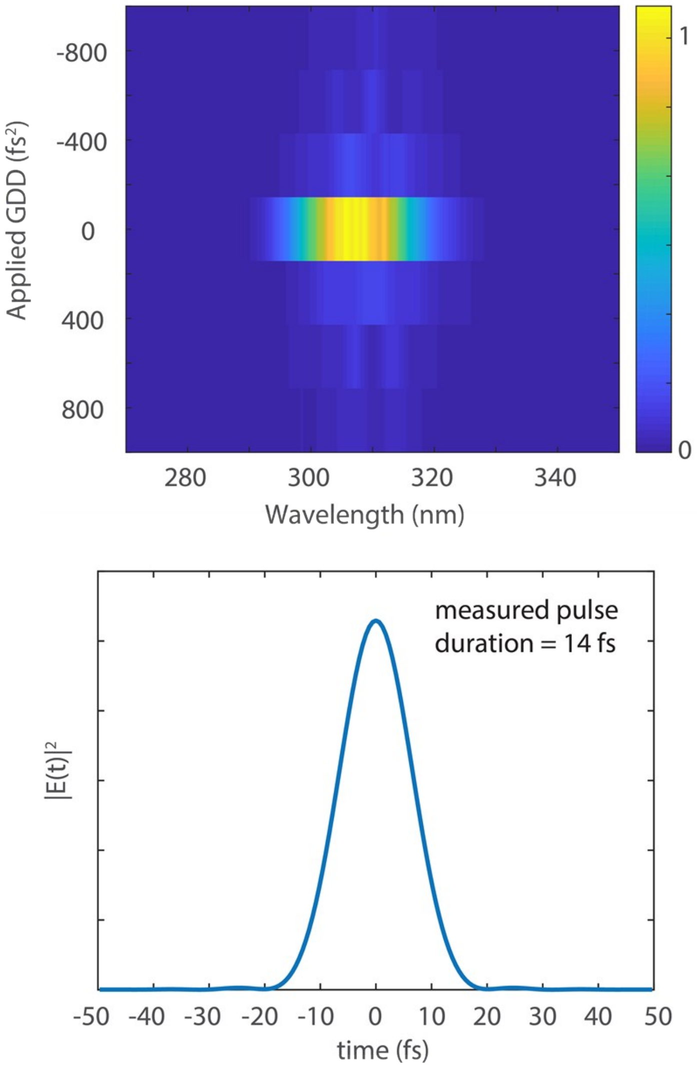

Figure S2 SHG signal during a chirp scan for the pulse compression (top) and estimated pulse duration (bottom).

S3. Fits of time traces in 2DES

Time traces of cross peaks are fit using the following formula[1]

$$y(t) = -Ke^{-\frac{t^2}{\tau_{pp}^2(1+2\varepsilon^2)}}\sin\left[\frac{2\varepsilon^2}{1+2\varepsilon^2}t(\omega_2-\omega_0)+\phi\right]+\sum_{i=1}^{2}A_i e^{-k_i t}\oplus IRF(t) \qquad \text{S1}$$

where the first term accounts for the coherent artifact and the second term is to fit the population dynamics. For the coherent artifact, we only consider the instantaneous electronic response since this

term is much stronger than the Raman scattering nearby time zero. Here $K = D_e^0 \exp[\varepsilon^4 \tau_{pp}^2 (\omega_2 - \omega_0)^2/(1+2\varepsilon^2)]$ where D_e^0 is the amplitude of the electronic response function. $\varepsilon = \tau_{prb}/\tau_{pp}$ where τ_{pp}, τ_{prb} is the pulse duration of the pump and probe, respectively. The pump duration is determined from a chirp scan. The probe pulse duration (τ_{prb}) is estimated to be around 10 fs by measuring the 2DES of crystal violet (CV) (see below). ω_0, ω_2 are the center frequency of the probe and the probing frequency, respectively. k_1 ($1/\tau_1$), k_2 ($1/\tau_2$), are internal conversion rates from E_3 or E_2 (or S_2) to E_1 (S_1) and from E_1 (S_1) to the ground electronic state. IRF is the instrument response function which is assumed to be a Gaussian function. To obtain a satisfactory fit, we need to include an empirical phase (ϕ) in the term of the sine function corresponding to the electronic response for Chl a. This term can be attributed to the high-order phase which can cause a small time zero shift (< 2fs). The fitting results are shown in Table S1.

Table S1 Fitting parameters for time traces

Peaks/nm	τ_{pp}/fs	τ_{prb}/fs	ω_2/fs^{-1}	ω_0/fs^{-1}	K	A_1	τ_1/fs	A_2	τ_2/ps	$\phi/2\pi$
Chl a 588-665nm	14	10	2.98	2.83	1.05	-1	26	0.26	>100	0.18
Chl a 620-665 nm	14	10	2.98	2.83	1.31	-0.28	20	0.59	>100	0.06
Bchl a 578-770 nm	16	14	2.44	2.39	-1.86	-0.36	89	0.96	>100	0
Crystal violet 615-665 nm	14	10	2.98	2.83	4.98	0.37	195	0.25	>100	0

S4. 2DES of crystal violet (CV) to determine the probe pulse duration

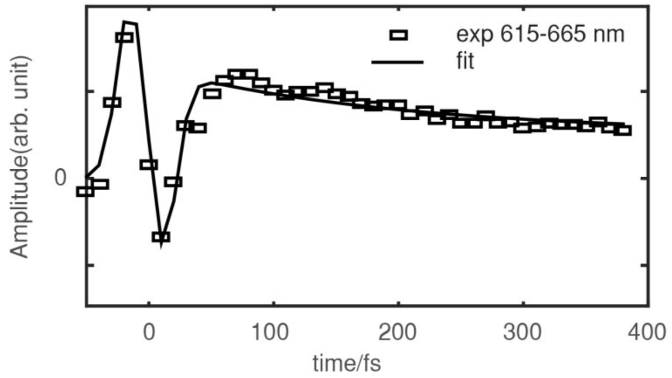

Figure S3 Experimental and fitting time traces for crystal violet in ethanol.

S5. Time traces of anisotropy and angle calculations

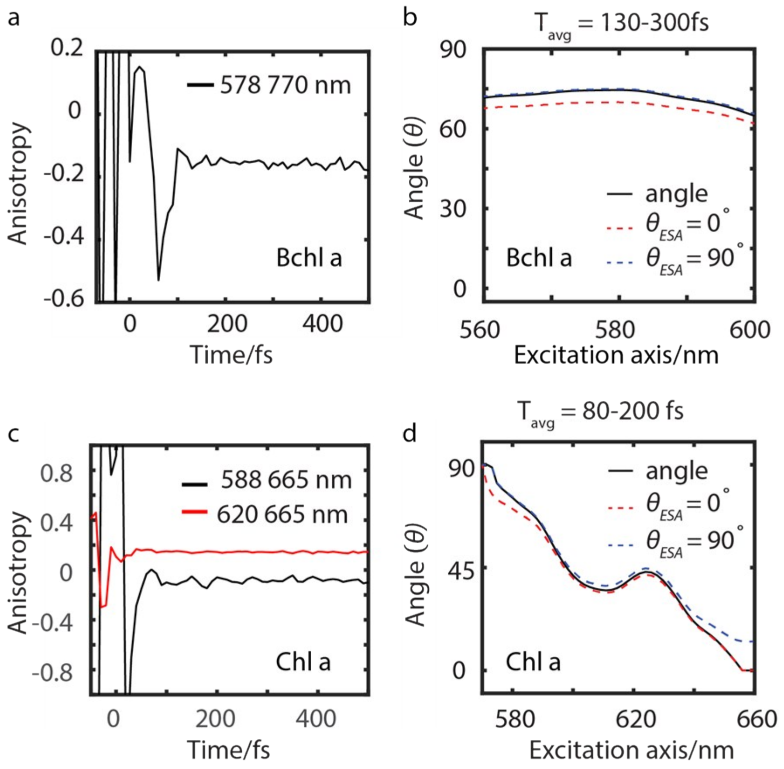

Figure S4 Time traces of anisotropy at cross peaks for Bchl a (a) and Chl a (c). The corresponding excitation and detection wavelengths are indicated respectively in the legends. The angle θ calculated from the measured spectra S_p and S_c using formula S2 is depicted in figure S4b (Bchl a) and d (Chl a). The black solid lines represent angles obtained when the excited-state absorption (ESA) amplitude equals to 0. Red and blue dashed lines show the angle obtained when the ESA amplitude is set to be 5% of GSB/SE. θ_{ESA} = 0° (90°) represents the cases where transition dipole moments corresponding to ESA and photoexcitation are parallel (perpendicular).

Figure S4a and S4c depict the dynamics of anisotropy for Bchl a and Chl a. We find that the anisotropic values for both Bchl a and Chl a remain almost unchanged after internal conversion is complete. Since the Stokes shift owing to vibrational relaxation occurs on the time scale of picoseconds, the constant anisotropic values also indicate that ground state bleaching (GSB) and stimulated emission (SE) originate from the same transition dipole moment. We note the potential interference of GSB and excited-state absorption (ESA) can alter our interpretation of anisotropy. To take ESA into account, we calculate the angle using the following formula[2]:

$$r = \frac{\eta\left(1 - 3\cos^2\theta_{ESA}\right) - \left(1 - 3\cos^2\theta_{da}\right)}{5(1-\eta)} \qquad \text{S2}$$

where r is the anisotropy, θ_{ESA} is the angle between transition dipole moments corresponding to ESA and photoexcitation, θ_{da} represents the angle between transition dipole moments for photoexcitation and detection, and η is the amplitude ratio of GSB/SE and ESA. We set η to be 0.05 in our calculations for both Bchl a and Chl a since previous studies showed that ESA is likely to be less than 5% of GSB/SE [3-5]. Figure S4b and d show the extracted angle for the limiting cases of θ_{ESA} = 0 °, 90°, indicating that a weak ESA has a small effect.

S6. Orbitals

All Orbitals shown in the following figures are calculated with the SRSH-PCM(ωPBE) approach in the 6-31++G(d,p) basis set.

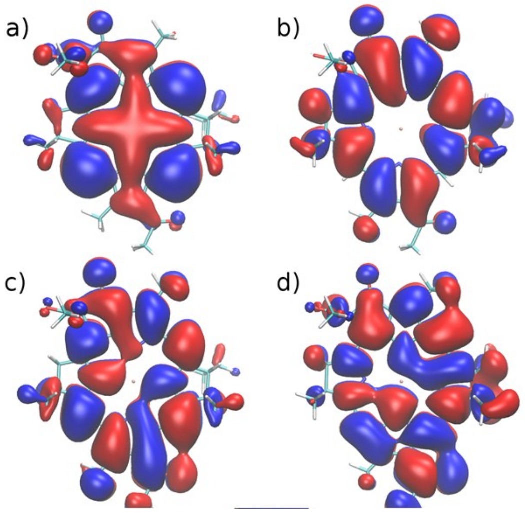

Figure S5 Frontier orbitals of the tetra-coordinated Chlorophyll a: a) HOMO-1, b) HOMO, c) LUMO, d) LUMO+1.

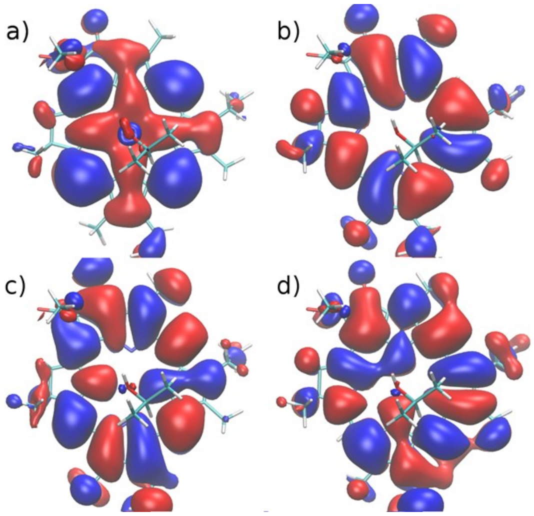

Figure S6 Frontier orbitals of the penta-coordinated Chlorophyll a: a) HOMO-1, b) HOMO, c) LUMO, d) LUMO+1. Note that the HOMO-1 orbital (a) changes upon ligation.

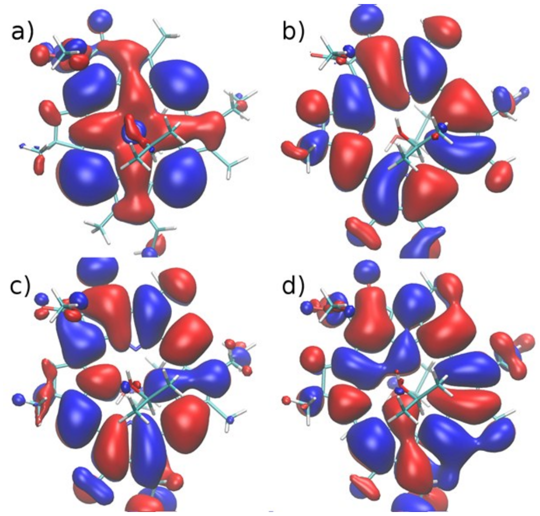

Figure S7 Frontier orbitals of the hexa-coordinated Chlorophyll a: a) HOMO-1, b) HOMO, c) LUMO, d) LUMO+1.

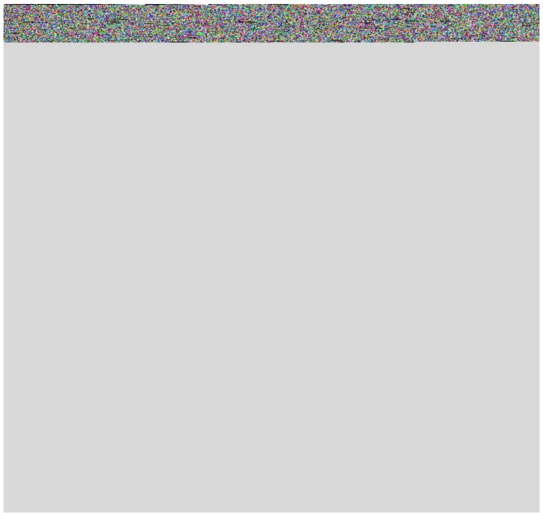

Figure S8 Frontier orbitals of the tetra-coordinated Bacteriochlorophyll a: a) HOMO-1, b) HOMO, c) LUMO, d) LUMO+1.

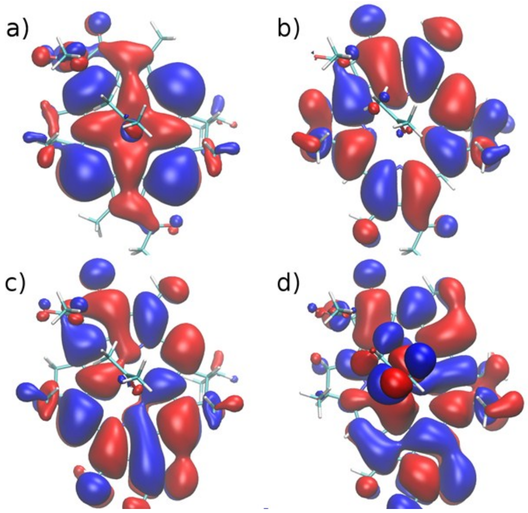

Figure S9 Frontier orbitals of the penta-coordinated Bacteriochlorophyll a: a) HOMO-1, b) HOMO, c) LUMO, d) LUMO+1.

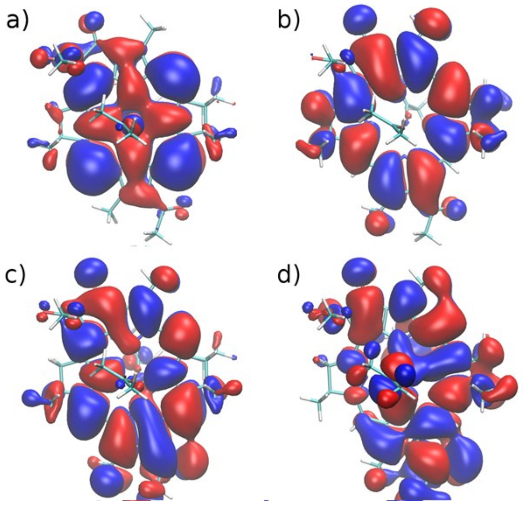

Figure S10 Frontier orbitals of the hexa-coordinated Bacteriochlorophyll a: a) HOMO-1, b) HOMO, c) LUMO, d) LUMO+1.

Table S2 Chlorophyll a orbital energies (upper rows, in Hartree) and orbital transition contributions (last two rows) are calculated within the SRSH-PCM(ωPBE) approach using the 6-31++G(d,p) basis set. The decreasing S_2 excitation energy with increasing Mg coordination can be traced back to a destabilization of the HOMO-1 orbital.

Coordination	**4**	**5**	**6**
HOMO-1	-0.199	-0.194	-0.190
HOMO	-0.188	-0.186	-0.184
LUMO	-0.110	-0.108	-0.105
LUMO+1	-0.080	-0.077	-0.075
S1	H→L: 0.95<br>H-1→L+1: 0.30	H→L: 0.95<br>H-1→L+1: 0.29	H→L: 0.94<br>H-1→L+1: 0.31
S2	H-1→L: 0.88<br>H→L+1: 0.47	H-1→L: 0.91<br>H→L+1: 0.41	H-1→L: 0.92<br>H→L+1: 0.38

Table S3 Chlorophyll a orbital energies (upper rows, in Hartree) and orbital transition contributions (last two rows) are calculated within the RSH(ωB97X-D) approach using the 6-31++G(d,p) basis set. Note that orbital energies and contributions to excited states differ from the optimally-tuned and screened approach reported in Table S2.

Coordination	**4**	**5**	**6**
HOMO-1	-0.261	-0.256	-0.251
HOMO	-0.244	-0.242	-0.240
LUMO	-0.053	-0.050	-0.049
LUMO+1	-0.017	-0.014	-0.012
S1	H→L: 0.91<br>H-1→L+1: 0.32	H→L: 0.90<br>H-1→L+1: 0.33	H→L: 0.88<br>H-1→L+1: 0.34
S2	H-1→L: 0.85<br>H→L: 0.28<br>H→L+1: 0.43	H-1→L: 0.86<br>H→L: 0.25<br>H→L+1: 0.40	H-1→L: 0.88<br>H→L: 0.28<br>H→L+1: 0.35

Table S4 Bacteriochlorophyll a orbital energies (upper rows, in Hartree) and orbital transition contributions (last two rows) are calculated within the SRSH-PCM(ωPBE) approach using the 6-31++G(d,p) basis set.

Coordination	**4**	**5**	**6**
HOMO-1	-0.201	-0.195	-0.189
HOMO	-0.179	-0.177	-0.174
LUMO	-0.116	-0.114	-0.109
LUMO+1	-0.057	-0.057	-0.051
S1	H→L: 0.99	H→L: 0.99	H→L: 0.99
S2	H-1→L: 0.95<br>H→L+1: 0.27	H-1→L: 0.96	H-1→L: 0.97

Table S5 Bacteriochlorophyll a orbital energies (upper rows, in Hartree) and orbital transition contributions (last two rows) are calculated within the RSH(ωB97X-D) approach using the 6-31++G(d,p) basis set. Note that orbital energies and contributions to excited states differ from the optimally-tuned and screened approach reported in Table S4.

Coordination	**4**	**5**	**6**
HOMO-1	-0.265	-0.259	-0.253
HOMO	-0.136	-0.233	-0.232
LUMO	-0.060	-0.056	-0.053
LUMO+1	+0.011	+0.015	+0.018
S1	H→L: 0.98	H→L: 0.98	H→L: 0.96
S2	H-1→L: 0.92<br>H→L+1: 0.30	H-1→L: 0.93<br>H→L+1: 0.29	H-1→L: 0.92<br>H→L+1: 0.24

S7. Primary Modes

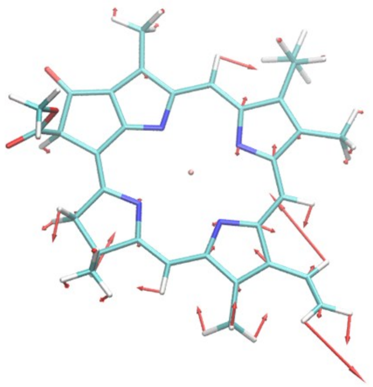

Figure S11 Illustration of the primary mode (1395 cm^{-1}) in the penta-coordinated Chl a S_1 state. Vectors were scaled and the acetone molecule was removed for clarity.

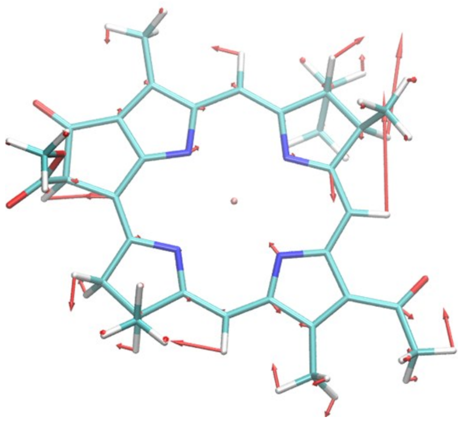

Figure S12 Illustration of the primary mode (1239 cm^{-1}) in the penta-coordinated Bchl a S_1 state. Vectors were scaled and the acetone molecule was removed for clarity.

S8. Structures

Structure optimizations of Chlorophyll a (Chl a) and Bacteriochlorophyll a (Bchl a) were performed using DFT and [TD]DFT calculations for ground state and excited state equilibrium structures, respectively. The conductor-like polarizable continuum model (C-PCM) was used in all calculations. In all structures, the phytyl-containing side group was replaced by a single hydrogen. Excitation energy differences to the full molecule—calculated for the ground state equilibrium structures of the unligated molecules only were around 0.01eV. If not specifically states otherwise, the 6-31++G(d,p) basis set was used.

Structure optimization based on the SRSH-PCM(ωPBE) functional did not converge properly and are not reported in this study. The poor convergence might originate in the structure-dependence of the tuning parameters α and β. Following the successful protocol of Ref. 6, 7, the dispersion-corrected ωB97X-D functional was used instead. Along selected degrees of freedom, single point calculations with the SRSH-PCM(ωPBE) functional were performed in the proximity of the ωB97X-D-ground state equilibrium structure. Results confirm that within the SRSH-PCM(ωPBE) approach the energy minimum is located at the same structure. The popular B3LYP functional was used for comparison as well. In comparison, ωB97X-D-structures are more compact than the B3LYP-structures, resulting in a shorter Mg-N and C-N bond-lengths, a weaker doming formation of the central Mg ion, and side groups being further bent towards the porphyrin ring. The total root means square deviations (RMSD) between these two structures of the penta-coordinated Chl a and Bchl a are 0.232 Å and 0.294 Å, respectively.
A sensitivity analysis of total energies and excitation energies with respect to these structural changes is outlined in the following section.

Table S6 Equilibrium structure of the S_0 state of tetra-coordinated Chlorophyll a in isopropanol (ε_0=20.18, ε_∞=1.90), performed with the ωB97X-D functional.

C	8.5569351007354850	-7.0311530331495522	-0.6560002085010675
C	8.7559707696380418	-5.7262046072591737	-0.8472765428407150
C	7.8038847964054492	-4.6612249767669374	-0.5382378455713233
C	6.4415896073331735	-4.6771813164561165	-0.5873680731009946
C	5.5331523194704122	-5.7930928484606410	-0.9852277060988048
C	5.9958181860452600	-3.3354525229283452	-0.2050152682230344
C	4.6575098880229850	-2.9316317263594414	-0.1336342477517114
C	4.1583704517303284	-1.6709933614476777	0.1902274310597293
N	4.8867329571894125	-0.5848966879899939	0.4846781139869104
C	4.0596848930876170	0.4927316448286043	0.7407470007895290
C	4.5433302188305369	1.7352209452799272	1.0076527360747425
C	5.9182924596320499	2.0766615241023696	1.0831955014909473
C	6.1667120964106630	3.4339251520826801	1.3738731394381734
C	4.8971129528414536	4.1188325392039218	1.5101268715479601
O	4.6489900215107962	5.2841966171169759	1.7318258970732385
C	3.7796815683603500	3.0106125750539348	1.3088660810011381
C	2.8412283886280609	3.4690694936291280	0.2145762391908333
O	2.9482735448931687	3.1871737829693796	-0.9543271879177075
O	1.8964710109155480	4.2692732691062201	0.6989976697283175
C	1.0276162538282552	4.8855681547535772	-0.2627457042092405
C	7.5537918031783464	3.5884778806788034	1.4288967002488677
C	8.3348157118328015	4.8302499120166562	1.7088290828024504
C	8.0738330038372297	2.2827044526633014	1.1632187684901329
C	9.4131361351685960	1.8733934464564943	1.1139033359205543
C	9.8845795323661001	0.5937405649952473	0.8461187352267738
N	9.0918343973505529	-0.5073771241718404	0.5799346169131074
C	9.9199601011022942	-1.5400037102505542	0.3411391064445562

C	9.4890119070176411	-2.8542123142897649	-0.0028295452063990
C	8.2009449690776925	-3.3040779339124069	-0.1461683639253589
N	7.0636381577143421	-2.5500187554811125	0.0497757758357580
Mg	7.0190932258415977	-0.5832301702735811	0.5265641467838208
N	7.0357487942415196	1.3825975343272556	0.9519766343403827
C	11.2981975590422241	-1.1253365929430157	0.4544420325419967
C	12.4872850407931200	-2.0080447516607336	0.2391396697211460
C	11.2761325393233101	0.2061834387653323	0.7792683852830846
C	12.4417429914545892	1.1310535684504621	0.9585068399230365
C	12.7757607880043640	1.8934627393006329	-0.3304566267820463
C	2.6143434836288182	0.0781640135365354	0.7002355468268243
H	2.0197451698362450	0.7257266134353422	0.0539008683792383
C	2.6650853359608599	-1.3759622072991877	0.2033787029885459
C	1.8409370088257591	-2.3382225653916993	1.0571892533775931
H	7.6436505707427509	-7.4199318118305682	-0.2193988225511169
H	9.3224947315862163	-7.7527460171613383	-0.9182017853696114
H	9.7113625717583894	-5.4025933503851329	-1.2533913822989642
H	5.2028344947493927	-6.3661448968933696	-0.1125854706565525
H	4.6410007156445001	-5.4147237527933374	-1.4879660953183336
H	6.0474579358647871	-6.4826777814297563	-1.6576518028523308
H	3.9147256529549996	-3.6859513794964811	-0.3630424397219326
H	3.2194942625521374	2.9593416575518248	2.2472709337242422
H	1.6118107741746686	5.4989161269427989	-0.9494890605515475
H	0.4723488722821486	4.1302876240881679	-0.8196352197711109
H	0.3519223360737622	5.5061835568090647	0.3193933428378036
H	8.7686693606386736	4.8012970494460676	2.7128620314403316
H	9.1536211557080787	4.9544887929322119	0.9962873656095325
H	7.6866763650348000	5.7051872551522562	1.6482784956433247
H	10.1615383270630275	2.6374781929406024	1.3005666988577202
H	10.2781270088066830	-3.5763415886450205	-0.1786880621632315
H	12.4918302180514118	-2.8460053499948335	0.9432417686374576
H	12.4900626097311775	-2.4279498149387160	-0.7709297313248727
H	13.4185618138717260	-1.4550906054040822	0.3721719550426252
H	12.2260099370765420	1.8480974614745196	1.7562301833454756
H	13.3167333822562330	0.5622064969004620	1.2843576936543746
H	13.0484686690977068	1.1952886883268858	-1.1263390451715110
H	11.9131006429265849	2.4731449103571124	-0.6702856541104260
H	13.6089541604184863	2.5823532618785872	-0.1704503052608907
H	2.1869483737566919	0.1376015261699439	1.7068970025017138
H	2.3084261843281682	-1.4277598369964906	-0.8308264939418302
H	2.2176906859997638	-2.3541202133745531	2.0838631080553274
H	0.7991520940765132	-2.0089719546464471	1.0781427313916399
H	1.8663509516665773	-3.3557169032414795	0.6613904970216266

Table S7 Equilibrium structure of the S_0 state of penta-coordinated Chlorophyll a in isopropanol (ε_0=20.18, ε_∞=1.90), performed with the ωB97X-D functional.

Mg	1.1079061872220093	0.2350565092626591	0.6885289730734077
C	-2.2553266880023219	0.9168609771698891	0.3328653767653848
C	0.3033228241278346	-3.0320893818262076	1.2690082863578911
C	4.3850807770522051	-0.6463685069023354	0.2659034881948060
C	1.8333826593597147	3.3758942146458830	-0.6665371647081452
N	-0.7469709518587698	-0.8839554525812552	0.8059274427617603
C	-2.0230987393056794	-0.3681080601678658	0.7151889662023108
C	-3.0545606276095025	-1.3998095462743889	1.0931912798857377
C	-2.2197697674871120	-2.6684147366514743	1.3448814146040868
C	-0.7928953200926271	-2.1775872080462761	1.1397256893665808
C	-2.4521168171416723	-3.2848251381815015	2.7250754934257002
N	2.1506014494348484	-1.5338658015860711	0.7050944805562537
C	1.6550633910943879	-2.7384525154342882	1.0551586472730676
C	2.7416879761070643	-3.7138525736855099	1.1792966040741171
C	3.8977060979139679	-3.0391183014950576	0.9193901799949644
C	3.5171575851126700	-1.6568586134727283	0.5991550434074773
C	2.5546037936300352	-5.1595506942177085	1.5022412351839254
C	5.2753602242794013	-3.5258223877423225	0.9102140912304619
C	5.7552986768002601	-4.5324439436930621	1.6426708803353998
N	2.8112749082661068	1.1959601897971051	-0.0893441863507866
C	4.0585949204394645	0.6988018697928871	-0.0739085766173712
C	5.0189695737852853	1.7070919366067396	-0.4587406323565323
C	4.2998862507015270	2.8470105278838624	-0.7097761508732463
C	2.9090121824585355	2.5142610812665063	-0.4866927318969681
C	6.4966393420793871	1.4956323433443390	-0.5662488836176434
C	4.8158309460224409	4.1698766133181904	-1.1885955797069372
C	4.7484202639284705	4.3067891265750555	-2.7141381238533824
N	0.0637470521368350	1.8197150900285282	-0.0518074595481577
C	0.4760382998654442	3.0704857064752011	-0.4948368964164124
C	-0.6565645694653695	3.9108079389201826	-0.7428609800988738
C	-1.7600188725450738	3.1144364258635724	-0.4333591272355460
C	-1.2557612927989605	1.8619582197892564	-0.0211603565561804
C	-0.6407731254033117	5.3162848065568467	-1.2477409184786115
C	-3.2059961346987706	3.0739056161256477	-0.3586605627420256
O	-4.0367071439228708	3.9224470231886057	-0.6137119016972534
C	-3.5867665128128823	1.6370311349865234	0.1560503524991629
C	-4.3655502816166969	1.7247865009784544	1.4524036205373507
O	-5.4242315278650235	1.1832916349282736	1.6567563771923224
O	-3.7182100392803994	2.4515860195428587	2.3617352295930134
C	-4.3147343932909328	2.5193792407944513	3.6640503392341768
H	0.0711290361322088	-4.0528461953597059	1.5487829662050934
H	5.4398298487997216	-0.8950515601010485	0.2439667187521952
H	2.0694929476023223	4.3831834198647153	-0.9968575643659208
H	-3.5899617407059035	-1.0880510090140463	1.9956192347643300
H	-2.4308618858595308	-3.4242848016231608	0.5821031244667300
H	-2.2596622711236694	-2.5454262386559616	3.5080217288750730
H	-1.8002649907579975	-4.1442219088932859	2.8974365012678871
H	-3.4877026031247378	-3.6223572211326447	2.8134916130352874
H	3.3606008013549014	-5.7594185918005243	1.0740155462248893
H	1.6059725705483650	-5.5334191570707141	1.1118972098964865
H	2.5564909445064408	-5.3276955797356456	2.5843013560846351
H	5.1390618169027178	-5.0872890747677779	2.3406239686138783

H	6.7968392469915706	-4.8251137810836537	1.5709378549910391
H	6.9164130616009301	1.1336357570512117	0.3770716361530230
H	7.0088368256095004	2.4219877898630906	-0.8312166367128663
H	6.7344512018153413	0.7513612540369666	-1.3325371750229063
H	4.2511436069618398	4.9833104392254537	-0.7226589029405289
H	5.8513989620935147	4.2876615434376522	-0.8577552802341717
H	3.7145652840396393	4.2551354273823216	-3.0663117706191940
H	5.3081599543583744	3.5015044943666553	-3.1980095673160021
H	5.1685267439235618	5.2611732304872465	-3.0420758967922383
H	-0.0048418255408174	5.9528368761518777	-0.6270366512292819
H	-1.6491517724286111	5.7301078180549494	-1.2517572066469491
H	-0.2486715160017774	5.3574245887386036	-2.2682267360721813
H	-4.2363977278514957	1.1546866531999453	-0.5779809540320474
H	-3.6637903805463266	3.1588907254750249	4.2539349328370202
H	-4.3621824208461941	1.5209416797395827	4.1006984471440893
H	-5.3168530908221401	2.9447570323745045	3.6069437624518539
H	5.9608131527630084	-2.9910245025413458	0.2575219048508284
O	1.3216848988076149	0.7023255107297885	2.7187874091381845
C	2.6344272783957949	0.7629702174262479	3.3249194340594674
C	2.5794704535425779	0.1878598273574608	4.7280164998951086
C	3.1665778131802282	2.1844124146704536	3.2815480718692771
H	0.7218006841004053	1.2928699911925881	3.1843828869801749
H	3.5827515008862592	0.1664107060336665	5.1599347680962433
H	2.1833865323384631	-0.8295627821827296	4.7126955955515584
H	1.9425072292340084	0.8059438974077063	5.3685067462996088
H	4.1853853627820676	2.2127575281844369	3.6751518989890690
H	3.1759517002015514	2.5616929379229925	2.2569159718140601
H	2.5426978199025521	2.8406632112601837	3.8969897346721614
H	3.2471344555537778	0.1209602090164827	2.6899461488771808
H	-3.8006020859397958	-1.5278506625729427	0.3058274101323859

Table S8 Equilibrium structure of the S_1 state of penta-coordinated Chlorophyll a in isopropanol (ε_0=20.18, ε_∞=1.90), performed with the ωB97X-D functional.

Mg	1.1108969478342563	0.2254935062068482	0.6870609085257126
C	-2.2568452649172688	0.9234903895047095	0.3146048984521623
C	0.2944870920868819	-3.0284159923865488	1.3233528052408701
C	4.3934233832386580	-0.6321116365337236	0.2483042217333977
C	1.8348568726032464	3.3764091229188442	-0.6756168729855760
N	-0.7370471100922333	-0.8709477885725623	0.8104271588708157
C	-2.0118722350460558	-0.3624213963965111	0.7116942144336182
C	-3.0437847642213467	-1.3815226947408750	1.1031040118593514
C	-2.2218054345825342	-2.6560375186496787	1.3467205156191089
C	-0.7917972576378755	-2.1743278215392401	1.1661269491928281
C	-2.4874119471695817	-3.3039136916361063	2.7066265642766840
N	2.1634423834093495	-1.5460491788534052	0.7256703393280620
C	1.6624414879023000	-2.7618507539470061	1.1115973990717807
C	2.7336581478785180	-3.7155487802542839	1.2462880477786253
C	3.8993657237648076	-3.0378159648285838	0.9352204518408759
C	3.5080105517286868	-1.6781488082009450	0.6057456090666846
C	2.5862205256719104	-5.1538765804767888	1.6223287815951750
C	5.2713922676626472	-3.5211692043498655	0.8920941137640753
C	5.7672339631537852	-4.6080585674637478	1.4941931637173842
N	2.8000281857862408	1.1881880626720744	-0.1021549662810842
C	4.0689442616661902	0.6714369721030391	-0.0789102753382922
C	5.0324820737709359	1.7056669697110334	-0.4610205998088390

C	4.3151157263036417	2.8319566254075763	-0.7081661822071146
C	2.9067602023989076	2.4865146888145211	-0.4918288817606714
C	6.5075232765880981	1.4962106720122443	-0.5561838576476582
C	4.8108544007115279	4.1691385934429270	-1.1645339794295158
C	4.6956803922510009	4.3535094054395049	-2.6821747138704382
N	0.0673557756047538	1.8157843473129676	-0.0614265375102956
C	0.4826066411786532	3.0626720683226596	-0.5068565052615916
C	-0.6644900346662933	3.9125093475200239	-0.7622063141294790
C	-1.7628532222004778	3.1210279115880213	-0.4552302776919674
C	-1.2558838936254919	1.8596101095835451	-0.0361340232753709
C	-0.6291873566717097	5.3132184547910137	-1.2677295654929965
C	-3.2079377013583334	3.0755944088850993	-0.3847975549455966
O	-4.0392321288665949	3.9255818492226933	-0.6453722317969797
C	-3.5870757219495597	1.6412433796051367	0.1320459859274505
C	-4.3692996327582172	1.7325547157403987	1.4266114225833100
O	-5.4260797065574131	1.1871499109468444	1.6307706503055619
O	-3.7275891859859627	2.4659496657353057	2.3347355654324740
C	-4.3262870609381894	2.5336460840397756	3.6360052072036337
H	0.0456294349540084	-4.0395299161256890	1.6236217329676728
H	5.4474209230709070	-0.8817622134305680	0.2241949713077474
H	2.0787537955507895	4.3797583581928921	-1.0061559253003698
H	-3.5590772188352600	-1.0573193131943324	2.0134995455358471
H	-2.4274989268239673	-3.3932421457109627	0.5633726802294913
H	-2.2990318701340757	-2.5867052664737780	3.5106533284587638
H	-1.8517289475405527	-4.1770583053284787	2.8683071822578294
H	-3.5287311756976800	-3.6297367897934403	2.7673152433971144
H	3.2210431353853539	-5.7852924776423160	0.9936825172909322
H	1.5572687999712214	-5.5014755126779269	1.5192913085415480
H	2.8902003557300939	-5.3290383880436005	2.6603811953170289
H	5.1643004766624570	-5.2484226276092354	2.1260081263863615
H	6.8125097537369648	-4.8733523792014211	1.3816194430662470
H	6.9148380132888034	1.1325005597082796	0.3918179233696853
H	7.0225969596631961	2.4210083774370283	-0.8191644136680813
H	6.7452730892322590	0.7473312950959233	-1.3178537022597545
H	4.2540081419614042	4.9620573970711677	-0.6549004116648967
H	5.8548859188313811	4.2832223784331678	-0.8608614278742781
H	3.6549665540503495	4.2892276702812406	-3.0113630678253047
H	5.2633680856325515	3.5808769438780388	-3.2078360408837563
H	5.0850830831410523	5.3281502285405118	-2.9877021523328406
H	0.0087692900917246	5.9449165347030464	-0.6420099152586460
H	-1.6330446170223789	5.7377557787771183	-1.2840323114594561
H	-0.2221812783204833	5.3505253946149036	-2.2839829676626593
H	-4.2350569262842850	1.1561441270094825	-0.6018759703700414
H	-3.6785988508256584	3.1767882914458139	4.2255270047698268
H	-4.3706367085420661	1.5359388757767449	4.0746848979017365
H	-5.3299256625050813	2.9551955962547058	3.5767441300830081
H	5.9574222998111734	-2.9203690331992056	0.2993860476782444
O	1.3310846980314008	0.7034794881197616	2.7155277803126858
C	2.6413345134740944	0.7624172787184942	3.3261574200245621
C	2.5813248573332035	0.1838006828459054	4.7275270651190695
C	3.1736863873843766	2.1838695641802635	3.2885303578439471
H	0.7289146444863132	1.2913778958291544	3.1814269802434820
H	3.5830700598227798	0.1611640256911969	5.1629131248092595
H	2.1851388340283306	-0.8335096157860020	4.7080809625752620
H	1.9421109764343727	0.8004061126966916	5.3672043599242292
H	4.1910820301120824	2.2114470119442702	3.6857561520689566
H	3.1870453376751722	2.5632586138371503	2.2649725460125603

H	2.5474941344951008	2.8391775739219818	3.9025266463044570
H	3.2566370576999706	0.1221611315899579	2.6919840113436280
H	-3.8059600831612390	-1.4974040850748327	0.3289979750325870

Table S9 Equilibrium structure of the S_2 state of penta-coordinated Chlorophyll a in isopropanol (ε_0=20.18, ε_∞=1.90), performed with the ωB97X-D functional.

Mg	1.1195468105453317	0.2345371173487271	0.6421067532807770
C	-2.2446416809669114	0.9356866804700883	0.3157082840809963
C	0.2927771747561974	-3.0094649914239486	1.3041132847528740
C	4.3974075198448670	-0.6355919852290990	0.2296740539049177
C	1.8559699858823500	3.3837198793614833	-0.6825425945715453
N	-0.7592368841286220	-0.8733573884560564	0.7913471944472060
C	-2.0137750745266421	-0.3680350501007580	0.7097248978518758
C	-3.0573008730419309	-1.3806391149541963	1.1050397451777769
C	-2.2334154770040229	-2.6550519336700082	1.3730827071484808
C	-0.8025444846848263	-2.1774260632815761	1.1610092953088313
C	-2.4772759317830659	-3.2517680312545449	2.7592156226501094
N	2.1679336770327242	-1.5467811575280723	0.6932070431312842
C	1.6752163726260125	-2.7496550348582804	1.0916493101349447
C	2.7357230659050309	-3.7011442667316539	1.2470516743212456
C	3.9086399880086988	-3.0281035196314567	0.9324585349244453
C	3.5245176559208491	-1.6794654524899792	0.5848379612692680
C	2.5897951560937482	-5.1333533328355108	1.6491189741399119
C	5.2802717489937345	-3.5187416831596212	0.9014079399743721
C	5.7712093192581042	-4.6008297284031094	1.5140084229407174
N	2.8098902245537758	1.1892218343870862	-0.1258711184885122
C	4.0691753807899840	0.6851001881564044	-0.1013874400462367
C	5.0340974017874442	1.7097031885503169	-0.4711142357694112
C	4.3194749929272573	2.8447315605820682	-0.7149748514086314
C	2.9127298658263463	2.5080341557369863	-0.5080968294909149
C	6.5112569264249771	1.4998479511649063	-0.5638616857278131
C	4.8302617807646477	4.1776046001860498	-1.1679635570505309
C	4.7412059564283755	4.3537983407721361	-2.6884194271311745
N	0.0694150179945349	1.8410061928101171	-0.0753698617750653
C	0.4733463823976027	3.0870785185382030	-0.5158744126211409
C	-0.6467735036005519	3.9294298104410174	-0.7600246830126812
C	-1.7649313838063532	3.1331758080400367	-0.4479582579713766
C	-1.2595053504169780	1.8840578706104498	-0.0410196545079311
C	-0.6180964170196378	5.3365353362632320	-1.2635132809713749
C	-3.2022339424271471	3.0878608632032587	-0.3695426518044987
O	-4.0481713811195332	3.9312721966245343	-0.6120048006989141
C	-3.5734327672766986	1.6437969418612537	0.1380564015526548
C	-4.3653814403373739	1.7177469250752333	1.4292068276682932
O	-5.4497484274108414	1.2186830062525489	1.5990530186177445
O	-3.7010566842087398	2.3903096918269791	2.3663855836490808
C	-4.3148562982018879	2.4556162415157012	3.6611967871248408
H	0.0539250706361325	-4.0221447714503658	1.6119172836299400
H	5.4537602935491156	-0.8781372375164767	0.2101063387771073
H	2.1025797004229605	4.3914236464683523	-1.0019315943989500
H	-3.5845967590341705	-1.0490099264823698	2.0054521713322515
H	-2.4579142536004914	-3.4151776913716185	0.6184979538575642
H	-2.2818075547227088	-2.5051802120287752	3.5344936960394562
H	-1.8319303202919754	-4.1141446013056573	2.9434801426067820
H	-3.5152265588989620	-3.5826821326864171	2.8481111234476830
H	3.2181403247003488	-5.7743690009348283	1.0243297852395208

H	1.5604173378323449	-5.4836446017899139	1.5628615415547280
H	2.9048905879970333	-5.2871392604704921	2.6866637090572576
H	5.1651183554383087	-5.2371080908859922	2.1467890113741017
H	6.8166178481366249	-4.8681716328409887	1.4080197345192091
H	6.9191108086019604	1.1370025486708553	0.3842780111136508
H	7.0264385868348542	2.4253513032809213	-0.8246920041327593
H	6.7530218422188568	0.7532909248939214	-1.3264662189859455
H	4.2704056678911639	4.9771389700061537	-0.6727376349476265
H	5.8699734944153565	4.2872413109109599	-0.8480632558055765
H	3.7043065612778960	4.2980265570965557	-3.0304948381272898
H	5.3073055973908643	3.5709841023185716	-3.2006639834772215
H	5.1454031029838259	5.3222191664586882	-2.9944399806042385
H	0.0155815248230240	5.9729327003139501	-0.6387484994699366
H	-1.6241843341991795	5.7569529173164709	-1.2716333423345554
H	-0.2217140149595845	5.3833931220072255	-2.2830144209332599
H	-4.2092829353045831	1.1498453205844563	-0.6009079628542606
H	-3.6653178629504182	3.0855551243248276	4.2626483883720443
H	-4.3757057477085475	1.4545846221478844	4.0904048659290000
H	-5.3120873974097620	2.8903308752429351	3.5936749925899996
H	5.9703673110190270	-2.9243910849141375	0.3068952494495810
O	1.2959696568797483	0.6658016985609880	2.6801389604699279
C	2.6015387179578320	0.7319642086896236	3.3021387731513028
C	2.5337265405656213	0.1549576357802261	4.7036862533551442
C	3.1293607803775130	2.1548331307347302	3.2637598281078350
H	0.6868455742750367	1.2507298266025999	3.1408261596556302
H	3.5337943187888095	0.1326795598104979	5.1428776523669271
H	2.1377549368183471	-0.8623996262420371	4.6835143823747307
H	1.8921951207542413	0.7722909201100784	5.3402804560853419
H	4.1470183295485894	2.1848108549887089	3.6601420812215051
H	3.1409137892265258	2.5330568594609506	2.2395936202860929
H	2.5020399566284688	2.8086670925498529	3.8781004908746839
H	3.2231651898518203	0.0919737867813049	2.6739962033356925
H	-3.8073135915623677	-1.5114050715629397	0.3219539008919643

Table S10 Equilibrium structure of the S_0 state of hexa-coordinated Chlorophyll a in isopropanol (ε_0=20.18, ε_∞=1.90), performed with the ωB97X-D functional.

Mg	1.1860804039600747	0.1618544308638185	0.4166459277705012
C	-2.1417842802978577	0.9375442072541624	0.3299888860173441
C	0.4026447758500608	-3.0007700518271010	1.3881661074432863
C	4.4848947884040458	-0.6677661715214896	0.2582379023568369
C	1.9618900415936207	3.3923029416650912	-0.6340318194078877
N	-0.6563846342974088	-0.8766985815504907	0.8306150821361568
C	-1.9233823000145007	-0.3482915583516216	0.7288758785518098
C	-2.9603573558169844	-1.3662148902656928	1.1360084305639135
C	-2.1325121079231781	-2.6386288926739709	1.4036166298562540
C	-0.7004231209617584	-2.1537225920446414	1.2115414100494513
C	-2.3923015331690793	-3.2569072406777013	2.7771512895457664
N	2.2620817903000052	-1.5451247421366774	0.7616390830043454
C	1.7618560071054989	-2.7221040133337637	1.1796122943578720
C	2.8528780556329338	-3.6883128889925954	1.3567323080633509
C	4.0023584010484523	-3.0404330901114456	1.0122354922970895
C	3.6220672217730234	-1.6683174690578642	0.6445478837715818
C	2.6849547301794021	-5.0825732829988652	1.8621251582440248
C	5.3770463097913135	-3.5381776219299219	1.0085151214020178
C	5.7475292765689598	-4.7855062142793940	0.7154946631857361

N	2.9264661272949968	1.1902164724781032	-0.1104558488066143
C	4.1668666531625176	0.6824897835465409	-0.0837791235153916
C	5.1376921634654087	1.6930690389108913	-0.4454526678249162
C	4.4282383101002729	2.8416610842061076	-0.6822341454342875
C	3.0311805297664396	2.5152835466047687	-0.4743716113023583
C	6.6156370355472092	1.4761643316415958	-0.5376049887041209
C	4.9542136073576515	4.1686578932190645	-1.1374790937747374
C	4.8858525473887378	4.3283541520223014	-2.6607836548617096
N	0.1766560820506048	1.8542445254831257	-0.0398952618059603
C	0.5969653812464899	3.1028700901968564	-0.4647589897814541
C	-0.5346391719013360	3.9491824741714736	-0.7081087468668606
C	-1.6414606023546667	3.1498768493174203	-0.4124954918752630
C	-1.1399119885556570	1.8904435653820966	-0.0124971916793054
C	-0.5150314706577189	5.3591263825462674	-1.2005764315733478
C	-3.0870994052371619	3.1063589519483106	-0.3501508862776786
O	-3.9186246924390242	3.9552625737349163	-0.6035305222115526
C	-3.4712654341295575	1.6621916278783055	0.1448866616695795
C	-4.2724533018912716	1.7388513328844200	1.4277371323794361
O	-5.3434973557087462	1.2113466322890463	1.6030461453538722
O	-3.6322139275858518	2.4406440931939533	2.3615096738898078
C	-4.2525744133066707	2.4993669192134331	3.6527764262789697
H	0.1673361978353962	-4.0116892036666085	1.7010689901537777
H	5.5367991537920842	-0.9275477920417138	0.2147474716789704
H	2.2105796728875404	4.4021572749467994	-0.9474500158596249
H	-3.4806761599749194	-1.0353097585255790	2.0405529588304949
H	-2.3353583717210946	-3.3921095286350322	0.6355864992063649
H	-2.2063304988040331	-2.5216739061229667	3.5655497371098535
H	-1.7502929673531802	-4.1223381264083567	2.9569780240704535
H	-3.4313860916848649	-3.5876269955535083	2.8490278145128238
H	3.6170806093112882	-5.4509878187033136	2.2952964661182311
H	2.4061695571565935	-5.7720158529813386	1.0582145444883890
H	1.9040722956357483	-5.1350256197607358	2.6244749784215977
H	5.0266634142675795	-5.5438627185503950	0.4293054833539097
H	6.7903153902110081	-5.0811568888346201	0.7391104649967634
H	7.0238768169399970	1.1096942600980189	0.4091982084861859
H	7.1350264686751528	2.4010762913584274	-0.7938084970268552
H	6.8591627371710846	0.7328746101127389	-1.3030868257685937
H	4.3944672701535925	4.9781300754737607	-0.6588287919934319
H	5.9906693830020563	4.2750131675490390	-0.8054322718031950
H	3.8516045696254833	4.2803324007502583	-3.0124560252303576
H	5.4458650285753798	3.5303921303776020	-3.1564062107845925
H	5.3052357008475539	5.2875058653420366	-2.9757595162974009
H	0.1212477265805424	5.9899812323932151	-0.5743224479406636
H	-1.5224116460905959	5.7755322023890354	-1.2037131852865310
H	-0.1209120614792860	5.4074068231309989	-2.2200815309147122
H	-4.1108247461642913	1.1898945854718534	-0.6045864983714885
H	-3.6109425133365818	3.1338666790497465	4.2579982885960970
H	-4.3082728618781765	1.4979354513587708	4.0816722979132072
H	-5.2530265014062918	2.9265167021828704	3.5814646172588822
H	6.1479553161779625	-2.8096538348851259	1.2495877487257474
O	1.3334754145636112	0.6764665427402718	2.5446930158326877
C	2.6035149019923018	0.7498069988554072	3.2193833962783538
C	2.4917469579509364	0.1682813277456356	4.6182028640904553
C	3.1323081100828380	2.1740132699136772	3.2101410693463164
H	0.7364890186788915	1.3358441663819467	2.9100600515974544
H	3.4786280074276639	0.1401162489347189	5.0872815927846142
H	2.0938407458152946	-0.8483189216577005	4.5827683104353802

H	1.8335230183747120	0.7832547650460203	5.2401115856039526
H	4.1413463679097910	2.2027812923792989	3.6285980282300714
H	3.1647913620378381	2.5626718644208126	2.1905938530027340
H	2.4919156199902854	2.8222751696751298	3.8178007450425691
H	3.2541831893065649	0.1154984588471707	2.6169022540150610
O	0.8521317546092811	-0.7432666047482817	-1.6765179874028355
C	1.8393392369939536	-0.5911027230741125	-2.7142866900985854
C	1.7288814779093902	-1.7278434381722483	-3.7162779890927986
C	1.6103149861964001	0.7672500821842241	-3.3465926136838453
H	2.8309994174578943	-0.6126767324749626	-2.2476939938175318
H	2.4810572159353690	-1.6108202946103467	-4.5001045876199335
H	0.7366736060854442	-1.7299192072250487	-4.1766235622129466
H	1.8905820848496573	-2.6951073776089869	-3.2307226977719044
H	0.6183674712682820	0.8035841057431677	-3.8063854132633335
H	2.3629563092724033	0.9551914912973144	-4.1158735373794570
H	1.6799422468167589	1.5538749563633303	-2.5944007262032045
H	0.8480971468654384	-1.6665792093022311	-1.4050003514182334
H	-3.7173217016832720	-1.5034225408685946	0.3608314975749463

Table S11 Equilibrium structure of the S_0 state of tetra-coordinated Bacteriochlorophyll a in acetone (ε_0=21.01, ε_∞=1.85), performed with the ωB97X-D functional.

Mg	0.9030203986397485	0.3759519754533999	0.2580544251891094
C	-2.5230795908249029	0.5707723350813076	0.3756152609897896
C	0.6951320633474237	-2.9401691343399348	0.9852311346310093
C	4.2881231262203059	0.1799976034114572	0.1946028030023485
C	1.0974958172844049	3.7686755494817592	-0.4844867084548707
N	-0.7186590806927976	-0.9945729749779683	0.6049891537869745
C	-2.0807701276366868	-0.6888566728211485	0.5856273871490337
C	-2.9040735645368239	-1.9330465663828664	0.8091601110366075
C	-1.8520525190497261	-3.0026223432985413	1.1511952975971915
C	-0.5455992192339688	-2.2731146214107341	0.8879467697310355
C	-1.9372325373291568	-3.5031777419189498	2.5957090177865969
H	-3.4628825384131687	-2.1831621574961826	-0.0969913479779718
N	2.2568336612573709	-1.1213914317765759	0.5178841452070293
C	1.9607971065815792	-2.4135831122667599	0.8227126158604795
C	3.2075633728769253	-3.1842096198510852	0.9731545556226568
C	4.2304023842791176	-2.3089273021097614	0.7416966722902436
C	3.6165751206740389	-0.9965984129031047	0.4551198917826464
C	3.2304261319165892	-4.6324541158265378	1.3390414059157494
C	5.6936248875724749	-2.5733111221119183	0.7615541395317081
O	6.4921096100419922	-1.7278536397145665	0.3970349558351045
C	6.2007193021158420	-3.9112626897438125	1.2349428079260663
N	2.4510982323912573	1.7600644656385094	-0.1051283323217351
C	3.7359607107111481	1.4575764132668716	-0.0575813593679410
C	4.6212409511954959	2.6700814517799847	-0.2636744612422006
C	3.6232706930214089	3.7267068145136415	-0.7709988456046294
C	2.2818220122754806	3.1092916058118272	-0.4223744976679772
C	5.3063127784376016	3.0657541599238183	1.0488009656794006
C	3.7448901416441007	4.0115986993102863	-2.2791255088109152
C	3.5263299039562015	2.7928918321697909	-3.1740018955462057
N	-0.3949773752015334	1.8903061011676174	0.0193808962746523
C	-0.2165054593027488	3.2324624174261856	-0.2721231606217078
C	-1.4532883430832413	3.9010164085928678	-0.3466582731348574
C	-2.4189520005860734	2.8997469111839966	-0.0879608771949272
C	-1.7101200668425556	1.7136415444187940	0.1230060076337167

C	-1.6743296185340555	5.3533573644041299	-0.6365459928749782
C	-3.8283097042747785	2.6200182489734845	0.0292532645398115
O	-4.8003375249666691	3.3471780036154857	-0.0656043739715124
C	-3.9557225289456421	1.0788953356543041	0.3317769216083224
C	-4.7102262622292645	0.8626073773385511	1.6259382293301397
O	-5.7302259349752598	0.2273119660988034	1.7355085669108090
O	-4.1032477841761388	1.4694355365392098	2.6439841851992889
C	-4.7197374678358406	1.3477394851796904	3.9319950933473180
H	0.6258922346645386	-3.9916687420380419	1.2298564235094374
H	5.3662410510618974	0.1247693455234977	0.1821058270294057
H	1.1491424911159438	4.8193223855393006	-0.7557151359730988
H	-3.6342252385960121	-1.7996047383298215	1.6111286000629506
H	-1.9260967266751152	-3.8573435927148014	0.4732644256047268
H	-1.8170276707676243	-2.6740795251371234	3.2994610820465700
H	-1.1657136876234793	-4.2471794429394514	2.8064512147819594
H	-2.9122140287823206	-3.9638489835575097	2.7716441586350493
H	3.8526585656513204	-5.2138419156034779	0.6587592985755251
H	2.2313444833741594	-5.0622717204310836	1.3243360353238967
H	3.6329592103015131	-4.7621854534265005	2.3472026566455080
H	5.7839470995019360	-4.1851757620040333	2.2053037174597065
H	7.2869470980357081	-3.8632059364954952	1.2968426524230374
H	5.9237157739524440	-4.6896395377474285	0.5189627478655111
H	5.3897185620199632	2.4493254606100128	-1.0102168481004390
H	3.7577501776943008	4.6715958771675332	-0.2372120061633906
H	4.5608050503757065	3.2717388221799175	1.8225974763666366
H	5.9052585470058299	3.9678812961942334	0.8998396373491002
H	5.9668312697596573	2.2712207261629582	1.4047766146308502
H	3.0156871523967244	4.7865143546446296	-2.5384648422192559
H	4.7381583213401521	4.4350219588368285	-2.4653559366727706
H	2.5305903111224466	2.3650614243759138	-3.0249127362353811
H	4.2650158082182479	2.0099679513171167	-2.9795625638435967
H	3.6099239323511205	3.0757993802806736	-4.2261528471104937
H	-1.1070136977747258	5.9881227761100009	0.0504245321577664
H	-2.7307442720713091	5.6102852752627523	-0.5462089715978039
H	-1.3516717978217319	5.6148314757653921	-1.6497966545139950
H	-4.5398576726250433	0.6114140017175457	-0.4645176614674810
H	-4.1100984245267078	1.9523498225700822	4.5980994327713143
H	-4.7202479930050103	0.3069864928742748	4.2586857709812227
H	-5.7413150874440380	1.7276805757066778	3.9029828528750907

Table S12 Equilibrium structure of the S_0 state of penta-coordinated Bacteriochlorophyll a in acetone (ε_0=21.01, ε_∞=1.85), performed with the ωB97X-D functional.

Mg	0.8893502150209797	0.2970541700720906	0.2592741110779854
C	-2.5592591422043585	0.4224230338941795	0.0899796299253974
C	0.6568364887885245	-3.0334244890024880	0.9491144352973799
C	4.2360925218538439	-0.0303475914434182	-0.2463197652017859
C	1.0518880995665176	3.5230947574147740	-1.0924907764502918
N	-0.7535904507857863	-1.1115378961731990	0.4524741656364079
C	-2.1152416512668260	-0.8139953913336420	0.4099975665375503
C	-2.9315849330084487	-2.0459444925768837	0.7162282848875058
C	-1.8799560991299245	-3.0264247500667341	1.2590837653503046
C	-0.5788364921293139	-2.3603084458687009	0.8475633500474499
C	-1.9338471401864004	-3.1589892456473749	2.7860209992262206
N	2.2104514068140495	-1.2839461776296504	0.2477878452885209
C	1.9170218053401160	-2.5453911845861894	0.6537826522401612

C	3.1592128785722142	-3.3302502175616429	0.7738481697103260
C	4.1791565949779725	-2.4856739238529313	0.4384432288402749
C	3.5645868880996296	-1.1826984494579651	0.1084264167960141
C	3.1809292623976844	-4.7610789224810581	1.2043073357834633
C	5.6369551455779190	-2.7699056650378648	0.3985707611750759
O	6.4262946504569420	-1.9606521340680425	-0.0578769828968185
C	6.1538727277635319	-4.0831306034316386	0.9287755893015842
N	2.4074545919691661	1.5539566989328180	-0.5684403038935650
C	3.6876170472870551	1.2352027156317271	-0.5637416801037041
C	4.5768167927282155	2.4181917831211850	-0.8898240474370618
C	3.5704757063000128	3.4409561885182613	-1.4472181139978300
C	2.2375485781787909	2.8678859381702866	-0.9980158693625109
C	5.2868416499340531	2.9096285330090561	0.3766975407132288
C	3.6318067298700627	3.5993136237343930	-2.9771623257343225
C	3.3665913917511241	2.3114771791738717	-3.7551641235395894
N	-0.4327969486432877	1.7019751970330022	-0.3899965738096681
C	-0.2583670620248637	3.0066540586232380	-0.8149262556187945
C	-1.4970965697338112	3.6627518132641788	-0.9642457893855501
C	-2.4613261824857777	2.6916636109167680	-0.6068189518261049
C	-1.7468386333155479	1.5335768511498773	-0.2766823077507317
C	-1.7134069409381196	5.0725970417919815	-1.4209894623072914
C	-3.8699483902270271	2.4226121355784689	-0.4582575937587632
O	-4.8454040760642876	3.1302817076571068	-0.6380586489602954
C	-3.9906652529195141	0.9326048405090557	0.0319495341904111
C	-4.6718156253546832	0.8695024315522184	1.3828070774881962
O	-5.6248813559805155	0.1751012295121242	1.6414710653559568
O	-4.0796576609920763	1.6673334287067871	2.2698227999679044
C	-4.6096779934069154	1.6474290232699935	3.6006775157929529
H	0.5910213436076561	-4.0550558679620439	1.2991469519542997
H	5.3123517867695620	-0.0997517075077829	-0.2996609223597459
H	1.0959871011271263	4.5444636674902466	-1.4594174676145228
H	-3.7323266452924613	-1.8518429637270108	1.4320107554836699
H	-1.9618997206822100	-4.0170716727369955	0.8069467839042217
H	-1.8449860289413047	-2.1720339332649106	3.2511523371202107
H	-1.1252889728734479	-3.7930289154818673	3.1580936343621162
H	-2.8858705449052930	-3.5968119812812338	3.0956794205611016
H	3.8067487268755769	-5.3705961395230286	0.5533103595844578
H	2.1814380249915599	-5.1910814610269469	1.1989552964412407
H	3.5761237047295449	-4.8465645432663775	2.2204934039050666
H	5.7449044935312976	-4.3181322724582882	1.9122395953855482
H	7.2405747147857360	-4.0301246047045352	0.9773254960762964
H	5.8730904029734221	-4.8903886679931885	0.2464325525909745
H	5.3292612419923877	2.1414561704444250	-1.6340245770189283
H	3.7311577211697928	4.4250054461023671	-0.9982476111391451
H	4.5542543078787112	3.1688659235529757	1.1472011488233327
H	5.8831660621058388	3.7983610896615199	0.1542515763223301
H	5.9534465631318803	2.1422634980708697	0.7787870935498366
H	2.8962903550693562	4.3573176691465818	-3.2677246904081940
H	4.6172513238683548	3.9965353680188049	-3.2454806510402197
H	2.3819628427829631	1.9028976436237930	-3.5093014834708027
H	4.1141488143008438	1.5447664443022320	-3.5310498142345863
H	3.3945668463932206	2.4964728032095174	-4.8315525515248767
H	-1.1428558365098707	5.7783731219738268	-0.8102450168132407
H	-2.7681023538767775	5.3447998255133715	-1.3625839834874462
H	-1.3873942151664382	5.2052317136494768	-2.4578056937707551
H	-4.6142255095756539	0.3681181294462150	-0.6645213685524068
H	-3.9610099078939558	2.2935909862674437	4.1863244975564813

H	-4.5947611922674305	0.6324061223969868	3.9989916383569448
H	-5.6309426092844452	2.0300154569162476	3.6049747585523169
O	1.1968517640846303	0.9053102876843349	2.2490851456316321
C	0.5237617853802136	1.3582463975396504	3.1699471356952396
C	1.1795833030016838	2.0001014491525018	4.3461791991652436
C	-0.9705186583170945	1.3179119269265029	3.1282332733356415
H	0.8216118242521000	3.0329904286835574	4.4096982993037575
H	2.2643416278763664	1.9777253773242025	4.2614021369642865
H	0.8513815261491741	1.5003531761375959	5.2620938179105474
H	-1.4152264578780747	1.5645202862734788	4.0911916593913116
H	-1.3124356761011093	0.3399287515346239	2.7855407235172596
H	-1.3049832045152470	2.0495845152111380	2.3853238059791155
H	-3.3913932441983179	-2.4208473573383231	-0.2032909305851895

Table S13 Equilibrium structure of the S_1 state of penta-coordinated Bacteriochlorophyll a in acetone (ε_0=21.01, ε_∞=1.85), performed with the ωB97X-D functional.

Mg	0.9100809407622080	0.2843647473841249	0.3049024026290740
C	-2.5502737146587493	0.4316534297452682	0.0879018467276911
C	0.6467959472129587	-3.0474702397904894	0.9580721751395536
C	4.2577906544278052	-0.0040894689068952	-0.2412981681966607
C	1.0284463400476760	3.5088163825608576	-1.0937067030520391
N	-0.7384543524410015	-1.0899970422543619	0.4843669220281779
C	-2.0760044672991418	-0.8139950210735527	0.4237643024429348
C	-2.9049630257551420	-2.0319477298820390	0.7278436552937951
C	-1.8721301528227323	-3.0198605203691264	1.2867743288428057
C	-0.5596544366164057	-2.3791232910069668	0.8774319714394947
C	-1.9560923570360804	-3.1404595257505727	2.8141248304008717
N	2.2383523139078871	-1.2929198398008430	0.2747198900222717
C	1.9458557983774232	-2.5741329384767080	0.6613212994536014
C	3.1525218283049150	-3.3476478158580369	0.7560925987948283
C	4.1962461683206040	-2.4840111324277316	0.4184964653670872
C	3.5804350018542390	-1.1973845460678791	0.1210100301538645
C	3.1858574694008404	-4.7863920832794893	1.1661318656929687
C	5.6399542292239699	-2.7547577986301595	0.3782785497069860
O	6.4521269804153416	-1.9186303714293358	-0.0041902992478752
C	6.1562679781534158	-4.1053035708333896	0.8216488837966237
N	2.3952576808783954	1.5595125905132072	-0.5239109835023735
C	3.7202719616566147	1.2303419934732651	-0.5295607351594608
C	4.5768822796785908	2.4355562341136316	-0.8558838662685936
C	3.5487878511436111	3.4287608434772334	-1.4215286859353002
C	2.2355028942539588	2.8279623961196561	-0.9721145897567749
C	5.2694848030741692	2.9671942547882106	0.4048876835366105
C	3.6015123811108523	3.5835507348459106	-2.9549046533559600
C	3.3513240192729477	2.2896901553937057	-3.7269192074588942
N	-0.4214225717475314	1.6866253936386946	-0.3775310803282198
C	-0.2436568408622141	2.9793137719509559	-0.8258788556690372
C	-1.5214716676861142	3.6414644689389291	-1.0010721757715344
C	-2.4624630053502536	2.6874232728431378	-0.6409657019539794
C	-1.7379057990525717	1.5171643396701084	-0.2787040717247403
C	-1.7090935299947683	5.0370108127748798	-1.4878093655473272
C	-3.8778017151765729	2.4062091161574091	-0.4972860973934454
O	-4.8483111522189724	3.1109817235585426	-0.6994757227401823
C	-3.9859219229650864	0.9272701315035361	0.0194602430228924
C	-4.6636487358727408	0.8896307219103810	1.3750074591876988
O	-5.6066508193595901	0.1889912546820704	1.6514199738325879

O	-4.0768872074422182	1.7133141745707450	2.2413423052371617
C	-4.5984433834401504	1.7112268357146065	3.5763798037840817
H	0.5766059048828704	-4.0712000445840113	1.3046192987471874
H	5.3338045919043058	-0.0789126515114279	-0.2965832681325831
H	1.0784520155178972	4.5245447266703529	-1.4699845219216729
H	-3.7163337586897431	-1.8187144957033632	1.4264247327609947
H	-1.9664836691986443	-4.0126585457914032	0.8420809276717198
H	-1.8577324853453621	-2.1522120508650002	3.2745002463656410
H	-1.1632490270352316	-3.7850586385502019	3.2004824117885682
H	-2.9201187079778417	-3.5612883235203703	3.1113731471791248
H	3.7988766452890781	-5.3907999705160696	0.4963197376539690
H	2.1857945858559749	-5.2176264896582660	1.1711041376826934
H	3.5984755569287277	-4.8986590725419781	2.1742846932374937
H	5.7628047829172049	-4.3979780359963412	1.7960323544344410
H	7.2440769184356073	-4.0593856687134942	0.8611177175512552
H	5.8651624320228786	-4.8736351303628256	0.0999148828777898
H	5.3379866494210724	2.1764863052300094	-1.5964828451720245
H	3.6702807920686809	4.4221063206788322	-0.9798495157954910
H	4.5285334611851633	3.2071651179153307	1.1735983585440364
H	5.8333094129743834	3.8748488290950176	0.1733048152388010
H	5.9610010258037205	2.2249876709655423	0.8110333092262560
H	2.8602175803828325	4.3340949798154726	-3.2479039005691424
H	4.5832611024327470	3.9896852823644435	-3.2201796562529630
H	2.3629109160858190	1.8798005457185569	-3.4994450785871876
H	4.0955421426013565	1.5256588339315778	-3.4838544375015279
H	3.3993516110480244	2.4685824333083994	-4.8035534077940412
H	-1.1330245569146364	5.7416572545754940	-0.8798278821068428
H	-2.7592013566545379	5.3280396929110516	-1.4593819672942983
H	-1.3500630539682161	5.1360934375334617	-2.5182578604465307
H	-4.6104050720239256	0.3482540130521090	-0.6643868768089166
H	-3.9470742242833623	2.3655927747870629	4.1496732979513444
H	-4.5797853334005589	0.7011988112994967	3.9867100141822038
H	-5.6201508353227885	2.0925164615764333	3.5812443095496431
O	1.2008003053543563	0.9037878757208141	2.2941582993110070
C	0.5210344367016427	1.3529695121289447	3.2115772537781573
C	1.1677841036095813	1.9928512252796184	4.3938150730043830
C	-0.9735544628386448	1.3132996787760769	3.1580666826730179
H	0.8089716722157847	3.0255886775160561	4.4563276899538042
H	2.2530718356781962	1.9711154390984285	4.3165766538655328
H	0.8331007262095366	1.4917795152239102	5.3065966038755441
H	-1.4252962039413561	1.5479186214224079	4.1207654971621359
H	-1.3118498371879030	0.3391397570978394	2.8019506572853796
H	-1.3031612640263528	2.0552988032082715	2.4235374636827043
H	-3.3575720193980998	-2.3989903240778294	-0.1991575683227703

Table S14 Equilibrium structure of the S_2 state of penta-coordinated Bacteriochlorophyll a in acetone (ε_0=21.01, ε_∞=1.85), performed with the ωB97X-D functional.

Mg	0.8865302642507131	0.2882858392955784	0.2481097128848868
C	-2.5649556497025543	0.4187546242233065	0.0760216714898373
C	0.6383552107121874	-3.0334295102045652	0.9677217127845598
C	4.2378600415437315	-0.0160615971395875	-0.2407046462436827
C	1.0431239258771521	3.5165867821876171	-1.1004856109603960
N	-0.7667862801887589	-1.1077854111812444	0.4617852457344724
C	-2.1099787628950093	-0.8247331015397799	0.4105813927783161
C	-2.9336861704750961	-2.0506740768715188	0.7202016050955080

C	-1.8909533253881272	-3.0215967746262575	1.2975802987636971
C	-0.5841892071664567	-2.3730635128076818	0.8720803206467155
C	-1.9646699712152633	-3.1096959807495264	2.8261964103812409
N	2.2271610837100253	-1.3011543463532917	0.2621484032836239
C	1.9401714970574471	-2.5754993245746420	0.6658530573441609
C	3.1440577954788358	-3.3454064835015558	0.7797483883267053
C	4.1888386560642914	-2.4825795523907770	0.4414645954477052
C	3.5770540951076089	-1.2050622315657800	0.1236331208919740
C	3.1766338962875786	-4.7790370538865439	1.2089106505645981
C	5.6344228497458815	-2.7466731348237867	0.4162965590047548
O	6.4420821696155652	-1.8983401012192127	0.0539191730177479
C	6.1516387918102522	-4.0993016841496521	0.8478163487431822
N	2.4016216210645234	1.5562917915491474	-0.5662409411022244
C	3.6981166873516260	1.2383846906661795	-0.5519806170182973
C	4.5774220864737982	2.4327772571078721	-0.8656679671361051
C	3.5645890021069087	3.4438175631609083	-1.4340315310794927
C	2.2344082032788335	2.8564526474923482	-0.9955991381758675
C	5.2678019492331272	2.9384268784558047	0.4056422439055019
C	3.6359844188804189	3.6031755586655287	-2.9633650646150556
C	3.3865789919635017	2.3138692444619555	-3.7439351731460575
N	-0.4427453285943140	1.6991644859753485	-0.4019895068768047
C	-0.2727319687421792	3.0035974263940433	-0.8283269505533674
C	-1.5093005130675554	3.6604458519205276	-0.9818853967107549
C	-2.4771137740972051	2.6871782597819300	-0.6250278754104255
C	-1.7585513844285612	1.5305615719935635	-0.2926984709510427
C	-1.7185777239735880	5.0698334023969531	-1.4410427404024710
C	-3.8815642149312919	2.4137528966013031	-0.4765180200077989
O	-4.8638279387199805	3.1156701576177128	-0.6536914629758954
C	-3.9931391811277566	0.9228996288895228	0.0170028399851418
C	-4.6718111712642543	0.8611828400215654	1.3711962416377736
O	-5.6359663041932597	0.1820868855856533	1.6265520219182767
O	-4.0638898011414106	1.6446184639699661	2.2597200790498562
C	-4.5924878209263662	1.6278980490461892	3.5917257935658387
H	0.5696553796382468	-4.0519040127658661	1.3321063256087178
H	5.3160323829427876	-0.0858535527751363	-0.2868122812889115
H	1.0920020030846480	4.5372809735061237	-1.4684167122664871
H	-3.7478330327400329	-1.8455469756651324	1.4176057857782989
H	-1.9792284221690106	-4.0226840864035269	0.8709148770745725
H	-1.8901483255695968	-2.1088353090830516	3.2634777004677726
H	-1.1543152886766426	-3.7236128704367131	3.2272469925923515
H	-2.9165664981680615	-3.5468898979304062	3.1385667519313865
H	3.7931837746891355	-5.3891454146812761	0.5485346297652058
H	2.1775445798026087	-5.2126068306071360	1.2128902366910719
H	3.5830904854787300	-4.8758368858874439	2.2203290341870856
H	5.7584698940790462	-4.3981385956368655	1.8205604429340485
H	7.2393444110667415	-4.0539551287679423	0.8872621379752355
H	5.8589836937309512	-4.8624627915707244	0.1212453650054600
H	5.3396863405217632	2.1679673951235721	-1.6039328827143571
H	3.7075974699947278	4.4300915206741962	-0.9838230622014454
H	4.5243018216326059	3.1862968945015773	1.1694772368347675
H	5.8519460199555118	3.8367250229628085	0.1878966743562651
H	5.9423991078008616	2.1816758191986172	0.8146882959671575
H	2.8984760299644408	4.3568181496062142	-3.2603318350279640
H	4.6210238256224851	4.0073147775727378	-3.2220406241400736
H	2.4008775571069907	1.9000169512620060	-3.5117744201866747
H	4.1349567199455093	1.5508978537453204	-3.5101305298898127
H	3.4271051920071880	2.4992372416752602	-4.8198693378197870

H	-1.1467348566560749	5.7746600043713361	-0.8298362174095091
H	-2.7721739912161634	5.3466908540749269	-1.3855144295983142
H	-1.3893974275736516	5.2006384032124897	-2.4774409825368577
H	-4.6143051918866806	0.3501698335113696	-0.6753938628191921
H	-3.9406291312951431	2.2721598802346015	4.1757945130864718
H	-4.5801391414607915	0.6128211829698123	3.9900725351990860
H	-5.6123355722579999	2.0139248062615405	3.5968759725465334
O	1.2050722120579085	0.8899811428118902	2.2291455318380620
C	0.5424625855706257	1.3497735824916632	3.1546658105213865
C	1.2113529062883790	1.9848381809645717	4.3264724373000583
C	-0.9523213923703187	1.3266935413941903	3.1210869926316254
H	0.8621564020173009	3.0206580652450277	4.3923042144805118
H	2.2953542627118373	1.9534023998584060	4.2354882231028270
H	0.8836861335341029	1.4875816485731970	5.2439137392178186
H	-1.3887840165829075	1.5782572082272854	4.0864342519195889
H	-1.3055969402446155	0.3518030825129967	2.7815743334736189
H	-1.2827729792881799	2.0626786026467236	2.3807349268268250
H	-3.3763457254639975	-2.4353515878545418	-0.2037855612947864

Table S15 Equilibrium structure of the S_0 state of hexa-coordinated Bacteriochlorophyll a in acetone (ε_0=21.01, ε_∞=1.85), performed with the ωB97X-D functional.

Mg	-0.7133911507892439	0.2764649153438034	-0.3443966840835552
C	2.6993002320930004	0.1400576311036327	-0.4651145688658559
C	-0.8242729042305281	-3.1092852851374020	-0.7881653546612197
C	-4.0952259906967381	0.3852938099760382	-0.1683167541705188
C	-0.5840835945530557	3.6976212637764467	0.3287998388956808
N	0.7684131360401234	-1.2753892486078613	-0.5907205438794747
C	2.1467455775365760	-1.0887154405306525	-0.5870410445174181
C	2.8581184158342916	-2.4133232379959564	-0.7173559155432712
C	1.7131961746700290	-3.4041302287455446	-0.9994503765506952
C	0.4747996334477563	-2.5492188738189228	-0.7707501726733368
C	1.7412576738974896	-3.9776435331907591	-2.4186252827499861
N	-2.2085051075536168	-1.1299471424314875	-0.4448020491586885
C	-2.0384614255213824	-2.4584335878586421	-0.6350614774301041
C	-3.3572278948403067	-3.1204309083947983	-0.6755783421987596
C	-4.2838486453462812	-2.1345978302189135	-0.5111376128424904
C	-3.5393434803660129	-0.8662994193629748	-0.3501229712707066
C	-3.5456322993557370	-4.5944999123167465	-0.8357709930544917
C	-5.7602862268568842	-2.2629991433058185	-0.4506547280513100
O	-6.4394811152175766	-1.4292972534544821	0.1226340009372549
C	-6.4213705796901968	-3.4393156769739930	-1.1242972782785035
N	-2.1292667235648763	1.8215466455508731	0.0194438325579289
C	-3.4300743410867960	1.6284526462142266	0.0039002204706408
C	-4.2058948274529655	2.9202796768908028	0.1663898343685289
C	-3.1103052429386309	3.9130375508771196	0.6033003815529056
C	-1.8300342411111818	3.1541061596063891	0.2860573595114758
C	-4.8836736050755025	3.3101954388106232	-1.1512971146932673
C	-3.1946940853944730	4.3015766586383712	2.0894262263005836
C	-3.0381464825228566	3.1301983990665119	3.0568536355227338
N	0.7233999473583317	1.7036306449943814	-0.2335540906341389
C	0.6777120491076021	3.0375080740229596	0.1151763317298241
C	1.9786604372703887	3.5632831724789833	0.2613441579216201
C	2.8376064034620394	2.4702607360005229	-0.0070482998022870
C	2.0068389972505334	1.3778611069563076	-0.2884462165829625
C	2.3648598994959436	4.9606250743531186	0.6382849325365286

C	4.2064437810198632	2.0286168520691832	-0.0382043174035829
O	5.2502520172943461	2.6281864794734160	0.1573564850118781
C	4.1746307820939226	0.4960964234590062	-0.4013603390893116
C	4.8929291242117419	0.2624023451665052	-1.7129329307725518
O	5.8604706541003520	-0.4442937234898963	-1.8585766727983630
O	4.3184693259966167	0.9400706544850072	-2.7063875469537071
C	4.9005499254138511	0.8035476683773829	-4.0074648777975961
H	-0.8537659936261204	-4.1814916557552770	-0.9366674083998752
H	-5.1748471246509631	0.4241506235146966	-0.1241725111532192
H	-0.5360705989824051	4.7530628682324423	0.5838801292423449
H	3.6056232776326569	-2.4008778358942724	-1.5148509375442085
H	1.7195743967859876	-4.2294745910391356	-0.2820896150894652
H	1.6854930892387081	-3.1762195750879649	-3.1617144608891188
H	0.9061221112513477	-4.6615047309428901	-2.5889838973257127
H	2.6701670672134350	-4.5297365879573270	-2.5828735192601910
H	-4.2652217606878500	-4.9785222313693636	-0.1110990095892572
H	-2.6101422101446010	-5.1328213765699466	-0.6903082868845669
H	-3.9205486190075112	-4.8369419006587266	-1.8342050359863713
H	-5.9734793291832897	-3.6538300998062283	-2.0962926593828390
H	-7.4846216431664763	-3.2266070613748257	-1.2322031433053702
H	-6.3068353365389802	-4.3309608959953296	-0.5014871861542921
H	-4.9739393298966590	2.8030931755872515	0.9372100035246179
H	-3.1676277165240729	4.8303454005133482	0.0103926965065790
H	-4.1393068575810146	3.4090676893428014	-1.9476072408764014
H	-5.3949122812509431	4.2703096379180341	-1.0436143183786837
H	-5.6183832842644703	2.5598583310275562	-1.4538953543907454
H	-2.4201014548860265	5.0474018864107002	2.2967136537395341
H	-4.1594564959640987	4.7940989825516294	2.2565591012138273
H	-2.0625888612853571	2.6516485913164449	2.9334737518344487
H	-3.8081537256241456	2.3688957925468350	2.9025031769847716
H	-3.1081329488346876	3.4758872024106000	4.0909549054633176
H	2.0574472696061807	5.6870032905974952	-0.1211212203391039
H	3.4484959593390943	5.0332851420739688	0.7451864706944821
H	1.9087406666279152	5.2683672829937667	1.5839946474593611
H	4.7180673135881213	-0.0630913978322301	0.3634057616253636
H	4.2868674069887645	1.4173955743156259	-4.6614886552299835
H	4.8775983323969720	-0.2373219170213432	-4.3325840358296306
H	5.9288419391992937	1.1668980614001370	-4.0012431146194087
O	-0.9940875409939672	0.5961780615657412	-2.5133725406322722
C	-0.4014294237590681	0.5507430266672998	-3.5810840731333298
C	-1.1144300353598569	0.8511569937481072	-4.8619580615155886
C	1.0518085118562368	0.1840176902062968	-3.6489762776862191
H	-0.6882872756229731	1.7659149368776486	-5.2877761677951201
H	-2.1832795863379255	0.9800629465319792	-4.6972078644360877
H	-0.9278158090013755	0.0524350555510526	-5.5847531138503053
H	1.4431138181840184	0.2245679570248092	-4.6645134535499517
H	1.1592722390999217	-0.8308166912886501	-3.2571178350510066
H	1.6343182736119004	0.8375847243681019	-2.9964712697985103
O	-0.7189693889726664	-0.0092265228844991	1.9067444487547787
C	-0.1361890905776872	0.3430271235884216	2.9200136265776386
C	-0.8067061515827630	0.2787784497544609	4.2582446527978002
C	1.2678267378416963	0.8723669166336983	2.8831108362313809
H	-1.8265091436240786	-0.0937715538179548	4.1762098218442194
H	-0.8032710757516058	1.2793492366332344	4.7018035265545057
H	-0.2178693812792909	-0.3631626659034577	4.9198136987171086
H	1.2384684873297018	1.8933422820550525	2.4874448411777559
H	1.7137933729713950	0.8925204965636785	3.8770405178479352

H	1.8747506986547817	0.2735356332611321	2.2026278450482160
H	3.3821842781145013	-2.6532713534414185	0.2124514654273967

Table S16 Equilibrium structure of the S_0 state of tetra-coordinated Chlorophyll a in isopropanol (ε_0=20.18, ε_∞=1.90), performed with the B3LYP functional.

Mg	1.0924672852841342	0.1265996819802883	0.2915031013015920
C	-2.2567911995970320	0.9509455865617548	0.3282763007161803
C	0.2969586015809870	-3.0471405347999374	1.2487649167713537
C	4.4068415828668854	-0.6556087190320166	0.2240276864061476
C	1.8511302445668669	3.3862858818932424	-0.7288457033851796
N	-0.7512342525362373	-0.8915470954582235	0.7347562226081961
C	-2.0227300659399878	-0.3633683731075779	0.6727149438758634
C	-3.0663835210834263	-1.4027272089271849	1.0213265062786567
C	-2.2331273156948894	-2.6623894152915133	1.3606626014003873
C	-0.7997499924915286	-2.1953216004332585	1.1044050442341378
C	-2.4580150494855282	-3.1750569961219237	2.7932164099619894
N	2.1662983781033978	-1.5594995000996843	0.6688311590305026
C	1.6659383545501087	-2.7692934344533442	1.0551571144925578
C	2.7516019501218310	-3.7247660180092699	1.2399663403770813
C	3.9236060234724150	-3.0468749965872099	0.9545303962246028
C	3.5378153787158340	-1.6810163160674436	0.5859845618378092
C	2.5830466355932598	-5.1593045417413936	1.6386809913340350
C	5.3034451248281060	-3.5249192409414558	0.9650372407438891
C	5.8030584985683014	-4.5604853703033941	1.6661581109366508
N	2.8211089828194629	1.1838252843557049	-0.1738192105820388
C	4.0860170296514671	0.6767260695478732	-0.1342801024138089
C	5.0525801312942606	1.6944149925679068	-0.5122740649428917
C	4.3311913664179755	2.8385263228934252	-0.7841939563763302
C	2.9294783772802808	2.5063046796288058	-0.5674327567291990
C	6.5385630345007026	1.4923058565383929	-0.5794850357185375
C	4.8539233011383933	4.1693234041759233	-1.2525043485568095
C	4.8521631146261699	4.3238714083873866	-2.7878234446260852
N	0.0707530751741821	1.8311492428287148	-0.1019911810012545
C	0.4899139346496105	3.0948242394245531	-0.5260415760298556
C	-0.6469472361622444	3.9666669236304442	-0.7116212968704728
C	-1.7582508584453758	3.1736078105504220	-0.3815355112808984
C	-1.2587542584062956	1.8925045446108395	-0.0190450930132732
C	-0.6316297576022148	5.3893832201870184	-1.1749749916786429
C	-3.2006548332080418	3.1453958255486643	-0.2863220772802685
O	-4.0366148768161683	4.0205069043395101	-0.5058711898868863
C	-3.5937958426496142	1.6761898658649830	0.1796984473300428
C	-4.4682534618505105	1.7691974938417643	1.4206116450386281
O	-5.6813499522239566	1.6360100475074049	1.4111649677337119

O	-3.7615720693881700	2.0607947749247777	2.5222416015281972
C	-4.5063226000311953	2.2277566284559174	3.7537917264849567
H	0.0579134280167264	-4.0607121183496959	1.5521885366502290
H	5.4639475593175248	-0.8980768522623381	0.2128677608742582
H	2.0935285839337525	4.3957210112682432	-1.0448429764867393
H	-3.6782169747994491	-1.0797530867172185	1.8716311437310735
H	-2.4660960444581614	-3.4758285205394981	0.6631480802743711
H	-2.2317926082063888	-2.3918828885778338	3.5257434363605098
H	-1.8283391166648808	-4.0432000275272699	3.0124065857047473
H	-3.5033776150138620	-3.4741611753187418	2.9253566546398724
H	3.3202701296122474	-5.7942952834903876	1.1365474273981029
H	1.5875488479597109	-5.5336963054023478	1.3863316755571264
H	2.7218229248886159	-5.2987149724663931	2.7187374149609913
H	5.2048099937914420	-5.1556406336720171	2.3475993863537998
H	6.8542192762151686	-4.8205869725050396	1.5822877137287406
H	6.9392985839434242	1.1295920602401586	0.3747378830788808
H	7.0539186606168434	2.4246891040372796	-0.8235366134331248
H	6.8114405216351823	0.7533807059764355	-1.3430094252539659
H	4.2588212391444653	4.9774361592760021	-0.8113257727354134
H	5.8759011646578978	4.3068649866657962	-0.8826433846320222
H	3.8396454576009842	4.2217649147694081	-3.1923222693701554
H	5.4814378949307025	3.5605532095905579	-3.2579627490061718
H	5.2367108917574310	5.3084214130369531	-3.0763543595189411
H	0.3802721422710295	5.8000840653527854	-1.2027237121853231
H	-1.2402937660848539	6.0201697463395361	-0.5180645028312515
H	-1.0560619807345308	5.4759115271035608	-2.1832667285607537
H	-4.2232678393380736	1.2403554537807693	-0.6040887628931263
H	-3.7564015371321546	2.4402279341208626	4.5140347394181415
H	-5.0477952220671458	1.3100487063608692	3.9916920498882242
H	-5.2055811679512809	3.0609232828626252	3.6579817538292212
H	5.9979462347170678	-2.9656734585743751	0.3401753553245581
H	-3.7496386488423901	-1.5649429816573488	0.1803919967439799

Table S17 Equilibrium structure of the S_0 state of penta-coordinated Chlorophyll a in isopropanol (ε_0=20.18, ε_∞=1.90), performed with the B3LYP functional.

Mg	1.2348504976861669	0.2613053576062905	0.7594403695936406
C	-2.1675901838349194	0.9580384960386676	0.4328319283579375
C	0.3996805727012511	-3.0364043252397575	1.2823935420437076
C	4.4876315472346642	-0.6564583280640992	0.1473743276024607
C	1.9174597563822262	3.3859663276064143	-0.7283188504787313
N	-0.6546575886734005	-0.8618190917101834	0.8840757042404049
C	-1.9289255164496453	-0.3519504955911570	0.7922020979117399
C	-2.9713213445167033	-1.4110461981397111	1.0878425799870211
C	-2.1297591114453729	-2.6623795582376069	1.4369834795835719

C	-0.6990047797023842	-2.1780190881531700	1.1957849065531341
C	-2.3552452342180330	-3.1695561279904969	2.8719903268544384
N	2.2621406692283133	-1.5363633445725215	0.7014045486737114
C	1.7613007988998430	-2.7598946470106411	1.0389849332041876
C	2.8353274938736770	-3.7464032555558218	1.0985045478128215
C	4.0021081007539561	-3.0723258729611445	0.7872544096372299
C	3.6206731564702648	-1.6798789956249700	0.5235221437825156
C	2.6594540828767599	-5.2031738749808181	1.4032172959507392
C	5.3691795145994679	-3.5755303486674701	0.6843777126909176
C	5.8887917797444711	-4.6565596033660261	1.2970542352353640
N	2.9104397503945250	1.2120154370880101	-0.1140806408652936
C	4.1644761326203641	0.6844502959149199	-0.1744971759388869
C	5.1135928586165331	1.6765184216674014	-0.6573611286463020
C	4.3878445426179349	2.8226005454061385	-0.9039746720000539
C	3.0026067874518048	2.5144459263163412	-0.5684410241507393
C	6.5844120680228668	1.4467599544848488	-0.8511971127508740
C	4.8889865160640440	4.1280880460898057	-1.4601177160352945
C	4.8198837032388013	4.2097426566418346	-2.9993158492086249
N	0.1583316593886071	1.8554173011324877	0.0176498450045555
C	0.5614463085030099	3.0931267352425182	-0.4892986168655469
C	-0.5846710201881413	3.9334849646335921	-0.7505337361392245
C	-1.6886168033886817	3.1457725700096053	-0.3866231624006777
C	-1.1726490722092093	1.8986917652180524	0.0685615975513294
C	-0.5842741012428603	5.3178053414501232	-1.3198707714560218
C	-3.1306580790762450	3.0960496571329807	-0.3253681322683601
O	-3.9780486973959905	3.9403792197692931	-0.6189118172046880
C	-3.5115311431740239	1.6555475536367426	0.2209807032396742
C	-4.3959637968280072	1.8002535224437159	1.4504595357284529
O	-5.5854628317085835	1.5278720509952048	1.4752662089390818
O	-3.7279951613870743	2.3002373229532154	2.5011168475180723
C	-4.4863113136049870	2.5257723686405225	3.7143319532447605
H	0.1645750743751866	-4.0628167193155447	1.5435690901707331
H	5.5363561771723901	-0.9187236190746160	0.0565897533023107
H	2.1457965757026467	4.3775237979783821	-1.1056940424106432
H	-3.6234684080828261	-1.1115477153984894	1.9165471075542935
H	-2.3508072918951246	-3.4819557308580311	0.7433016793896645
H	-2.1406792386881301	-2.3800519051009599	3.6014255915986033
H	-1.7153681171302613	-4.0286994974842454	3.0980550691469140
H	-3.3975558035941336	-3.4802155218012443	3.0027431639718078
H	3.3635582552156125	-5.8113737373907206	0.8257671973004497
H	1.6473919308920095	-5.5442813184064450	1.1692590862179468
H	2.8416872578896499	-5.4236181381699273	2.4633867765837811
H	5.3200061166396422	-5.2763183564546301	1.9819234764553706
H	6.9270240917000914	-4.9311770737872864	1.1346385628571438
H	7.0567181251124360	1.0649807367555975	0.0618335885830920
H	7.0951710803982229	2.3726815006951547	-1.1281515335171424
H	6.7785030209949291	0.7126609506738933	-1.6432503394599960
H	4.3160641930360244	4.9588234274190954	-1.0318045630649857
H	5.9274923403782473	4.2798287904411989	-1.1442518715391812
H	3.7901833475222557	4.0952461012856043	-3.3545552570165391
H	5.4245090740478012	3.4208970586245289	-3.4595747913415051
H	5.1953843201314163	5.1769447394842691	-3.3518594234383259
H	0.4203801072790782	5.7457407993670637	-1.3557404665848249
H	-1.2196847980576231	5.9844582768752721	-0.7266830549542148
H	-0.9847893610491865	5.3209041565346356	-2.3416012574057445
H	-4.1267167061282422	1.1605650857978336	-0.5384959919158976
H	-3.7697210588062409	2.9281228700255499	4.4286136389167332

H	-4.9041213123774936	1.5842782972439875	4.0764009115738968
H	-5.2878808932886310	3.2430872705715532	3.5267080683533716
H	6.0368600253991529	-2.9958728613806813	0.0486772538042679
O	1.2992565265281053	0.7494742064079871	2.8161668725078481
C	2.4018686415163093	0.8614561078354378	3.7702175402608131
C	2.0514119399693720	0.1106845784822929	5.0497664113296485
C	2.7423883408487635	2.3288167801114956	4.0043979654827666
H	0.5094775938822853	1.1732834792309093	3.1869752211119144
H	2.8979583348186386	0.1370637923085479	5.7437517607742041
H	1.8112788683561563	-0.9348755070794632	4.8356986814195588
H	1.1900929251095804	0.5740911341651872	5.5462284775589747
H	3.6012761600217509	2.4095113571398534	4.6787809559828828
H	2.9913545592326649	2.8261066508577395	3.0626357290212241
H	1.8966280811460015	2.8526137885247853	4.4660727398404072
H	3.2322896190797521	0.3633590748711734	3.2630194911510042
H	-3.6179922336233701	-1.5721757902599538	0.2177753558953973

Table S18 Equilibrium structure of the S_1 state of penta-coordinated Chlorophyll a in isopropanol (ε_0=20.18, ε_∞=1.90), performed with the B3LYP functional.

Mg	1.1108969478342563	0.2254935062068482	0.6870609085257126
C	-2.2568452649172688	0.9234903895047095	0.3146048984521623
C	0.2944870920868819	-3.0284159923865488	1.3233528052408701
C	4.3934233832386580	-0.6321116365337236	0.2483042217333977
C	1.8348568726032464	3.3764091229188442	-0.6756168729855760
N	-0.7370471100922333	-0.8709477885725623	0.8104271588708157
C	-2.0118722350460558	-0.3624213963965111	0.7116942144336182
C	-3.0437847642213467	-1.3815226947408750	1.1031040118593514
C	-2.2218054345825342	-2.6560375186496787	1.3467205156191089
C	-0.7917972576378755	-2.1743278215392401	1.1661269491928281
C	-2.4874119471695817	-3.3039136916361063	2.7066265642766840
N	2.1634423834093495	-1.5460491788534052	0.7256703393280620
C	1.6624414879023000	-2.7618507539470061	1.1115973990717807
C	2.7336581478785180	-3.7155487802542839	1.2462880477786253
C	3.8993657237648076	-3.0378159648285838	0.9352204518408759
C	3.5080105517286868	-1.6781488082009450	0.6057456090666846
C	2.5862205256719104	-5.1538765804767888	1.6223287815951750
C	5.2713922676626472	-3.5211692043498655	0.8920941137640753
C	5.7672339631537852	-4.6080585674637478	1.4941931637173842
N	2.8000281857862408	1.1881880626720744	-0.1021549662810842
C	4.0689442616661902	0.6714369721030391	-0.0789102753382922
C	5.0324820737709359	1.7056669697110334	-0.4610205998088390
C	4.3151157263036417	2.8319566254075763	-0.7081661822071146
C	2.9067602023989076	2.4865146888145211	-0.4918288817606714
C	6.5075232765880981	1.4962106720122443	-0.5561838576476582
C	4.8108544007115279	4.1691385934429270	-1.1645339794295158
C	4.6956803922510009	4.3535094054395049	-2.6821747138704382
N	0.0673557756047538	1.8157843473129676	-0.0614265375102956
C	0.4826066411786532	3.0626720683226596	-0.5068565052615916
C	-0.6644900346662933	3.9125093475200239	-0.7622063141294790
C	-1.7628532222004778	3.1210279115880213	-0.4552302776919674
C	-1.2558838936254919	1.8596101095835451	-0.0361340232753709
C	-0.6291873566717097	5.3132184547910137	-1.2677295654929965
C	-3.2079377013583334	3.0755944088850993	-0.3847975549455966
O	-4.0392321288665949	3.9255818492226933	-0.6453722317969797
C	-3.5870757219495597	1.6412433796051367	0.1320459859274505

C	-4.3692996327582172	1.7325547157403987	1.4266114225833100
O	-5.4260797065574131	1.1871499109468444	1.6307706503055619
O	-3.7275891859859627	2.4659496657353057	2.3347355654324740
C	-4.3262870609381894	2.5336460840397756	3.6360052072036337
H	0.0456294349540084	-4.0395299161256890	1.6236217329676728
H	5.4474209230709070	-0.8817622134305680	0.2241949713077474
H	2.0787537955507895	4.3797583581928921	-1.0061559253003698
H	-3.5590772188352600	-1.0573193131943324	2.0134995455358471
H	-2.4274989268239673	-3.3932421457109627	0.5633726802294913
H	-2.2990318701340757	-2.5867052664737780	3.5106533284587638
H	-1.8517289475405527	-4.1770583053284787	2.8683071822578294
H	-3.5287311756976800	-3.6297367897934403	2.7673152433971144
H	3.2210431353853539	-5.7852924776423160	0.9936825172909322
H	1.5572687999712214	-5.5014755126779269	1.5192913085415480
H	2.8902003557300939	-5.3290383880436005	2.6603811953170289
H	5.1643004766624570	-5.2484226276092354	2.1260081263863615
H	6.8125097537369648	-4.8733523792014211	1.3816194430662470
H	6.9148380132888034	1.1325005597082796	0.3918179233696853
H	7.0225969596631961	2.4210083774370283	-0.8191644136680813
H	6.7452730892322590	0.7473312950959233	-1.3178537022597545
H	4.2540081419614042	4.9620573970711677	-0.6549004116648967
H	5.8548859188313811	4.2832223784331678	-0.8608614278742781
H	3.6549665540503495	4.2892276702812406	-3.0113630678253047
H	5.2633680856325515	3.5808769438780388	-3.2078360408837563
H	5.0850830831410523	5.3281502285405118	-2.9877021523328406
H	0.0087692900917246	5.9449165347030464	-0.6420099152586460
H	-1.6330446170223789	5.7377557787771183	-1.2840323114594561
H	-0.2221812783204833	5.3505253946149036	-2.2839829676626593
H	-4.2350569262842850	1.1561441270094825	-0.6018759703700414
H	-3.6785988508256584	3.1767882914458139	4.2255270047698268
H	-4.3706367085420661	1.5359388757767449	4.0746848979017365
H	-5.3299256625050813	2.9551955962547058	3.5767441300830081
H	5.9574222998111734	-2.9203690331992056	0.2993860476782444
O	1.3310846980314008	0.7034794881197616	2.7155277803126858
C	2.6413345134740944	0.7624172787184942	3.3261574200245621
C	2.5813248573332035	0.1838006828459054	4.7275270651190695
C	3.1736863873843766	2.1838695641802635	3.2885303578439471
H	0.7289146444863132	1.2913778958291544	3.1814269802434820
H	3.5830700598227798	0.1611640256911969	5.1629131248092595
H	2.1851388340283306	-0.8335096157860020	4.7080809625752620
H	1.9421109764343727	0.8004061126966916	5.3672043599242292
H	4.1910820301120824	2.2114470119442702	3.6857561520689566
H	3.1870453376751722	2.5632586138371503	2.2649725460125603
H	2.5474941344951008	2.8391775739219818	3.9025266463044570
H	3.2566370576999706	0.1221611315899579	2.6919840113436280
H	-3.8059600831612390	-1.4974040850748327	0.3289979750325870

Table S19 Equilibrium structure of the S_2 state of penta-coordinated Chlorophyll a in isopropanol (ε_0=20.18, ε_∞=1.90), performed with the B3LYP functional.

Mg	1.2365576660054092	0.2594818129284902	0.7251019357602936
C	-2.1646513352072216	0.9705747513070864	0.4125276719648137
C	0.3857456419339823	-3.0309497704666630	1.2844028016527795
C	4.4948519820826016	-0.6574313958430886	0.1547694497623158
C	1.9219516495666318	3.3834495521992682	-0.7490026015780115
N	-0.6610466762733640	-0.8552057943019351	0.8645277338923537

C	-1.9252019972946766	-0.3491241626768332	0.7776253342358358
C	-2.9744738330139970	-1.3954018365686838	1.0864550005751190
C	-2.1409695999386544	-2.6481271345856290	1.4512572955785654
C	-0.7084058276974472	-2.1784654289320580	1.1949842741265799
C	-2.3641205098907081	-3.1296378952316055	2.8953577703923741
N	2.2692503119981247	-1.5505847558947539	0.6958939238883898
C	1.7697413283620171	-2.7746709505041696	1.0449007016697980
C	2.8263076342117155	-3.7533502152354505	1.1241138031473874
C	4.0098692211951166	-3.0777564938586845	0.8111219103757017
C	3.6300957453553364	-1.6997812822500942	0.5340161601725104
C	2.6535070502096039	-5.2074402589192124	1.4459444112821660
C	5.3754934104072731	-3.5751354023624851	0.7310816219316933
C	5.8783448819013460	-4.7184819383968231	1.2432047644694140
N	2.9108081850984777	1.2046685693036798	-0.1288335123435403
C	4.1743495511808959	0.6774875115433298	-0.1740259923095250
C	5.1229733486244768	1.6689619851760640	-0.6477243219380222
C	4.3935353433829016	2.8140350642373848	-0.9067756530506168
C	3.0063291848730969	2.5019979570535984	-0.5829808725232022
C	6.5970271431392433	1.4484186817207194	-0.8254540958845576
C	4.8967061473877358	4.1190792535878993	-1.4600300960610668
C	4.8333170401284420	4.2015991227761651	-2.9997230518444886
N	0.1539394220676915	1.8666690531572505	-0.0211079500037525
C	0.5557572584248545	3.1118294400203066	-0.5179611633306267
C	-0.5839357812765429	3.9616442515559753	-0.7581059156852836
C	-1.7011693607435692	3.1732541164425174	-0.3913365173698836
C	-1.1772218504617087	1.9196339012628914	0.0426308741218720
C	-0.5719947471642256	5.3522709094858847	-1.3120965161252349
C	-3.1310069655473449	3.1217898371530195	-0.3225901651162504
O	-3.9987132991817385	3.9677175584913078	-0.6024559812195310
C	-3.5009343868247593	1.6700690945112076	0.2167118661647940
C	-4.3773144419207224	1.8079649937387083	1.4511671981678800
O	-5.5927125377700149	1.6988387366906830	1.4401787139399180
O	-3.6707795475683720	2.1189692021907685	2.5495528389583737
C	-4.4208956958140231	2.3404753948732986	3.7686775212743409
H	0.1450552194453450	-4.0536398905808460	1.5571431779635285
H	5.5464626557828209	-0.9130585897956461	0.0754598603909132
H	2.1630795399445968	4.3724854686193266	-1.1262117517917551
H	-3.6233575913582072	-1.0785342153033277	1.9112251675243408
H	-2.3746000131289819	-3.4757297484184360	0.7716880379756450
H	-2.1419179802746529	-2.3297293406951334	3.6109099685097386
H	-1.7278501591994480	-3.9887084386641467	3.1319851171367894
H	-3.4074901691254635	-3.4330538224717335	3.0349354970171349
H	3.2839136103923598	-5.8296190836547384	0.8009792829647886
H	1.6185880915846329	-5.5327266588245640	1.3113120365256761
H	2.9359462182619160	-5.4354195876424720	2.4825551726662707
H	5.2877170358770806	-5.4211802933902193	1.8201656657876886
H	6.9267018815721757	-4.9632724307598046	1.0997398538549588
H	7.0580764768260744	1.0514315966370573	0.0866431958856207
H	7.1076153698160951	2.3813629272008150	-1.0776433869209787
H	6.8047856038200711	0.7306874859942861	-1.6289671644366279
H	4.3223615759292162	4.9498202203399897	-1.0333869376923825
H	5.9340704915242037	4.2706254918747959	-1.1404988918871544
H	3.8050175261009977	4.0859613586133596	-3.3586736780305504
H	5.4407100798747079	3.4138103866617029	-3.4581177158054075
H	5.2089168637866701	5.1695638321339858	-3.3499756280229804
H	0.4300189859104479	5.7897024588107611	-1.2931293175858263
H	-1.2381300380790203	6.0102082680010174	-0.7425336535561686

H	-0.9195323398905761	5.3736724395854667	-2.3542924149135982
H	-4.1158446108515303	1.1717614814050947	-0.5417302039053028
H	-3.6723786999394723	2.5628100902465927	4.5275877136374403
H	-4.9824855115257192	1.4420631717202343	4.0325429976534926
H	-5.1026534155482590	3.1838761993874454	3.6412815921153330
H	6.0737258186306402	-2.9319302051458109	0.1975403639824751
O	1.2755117464214965	0.7417132129935461	2.7766156753264331
C	2.3725906974269053	0.9003707039488923	3.7321219121601197
C	2.0374345322473673	0.1613178420528920	5.0222403267879976
C	2.6712820131218087	2.3803899796860617	3.9425794791832054
H	0.4676792898895860	1.1298017127900928	3.1476586681535270
H	2.8810490400178028	0.2216923452798508	5.7174767908067547
H	1.8255981945644235	-0.8934104847239265	4.8240061493226509
H	1.1628913149559010	0.6100567443851956	5.5089785371790372
H	3.5198176343866687	2.4955595287594985	4.6250204970804942
H	2.9184933509143840	2.8672412892511496	2.9949822432172200
H	1.8066093441783120	2.8895997346151190	4.3850190414581967
H	3.2177975169482171	0.4166486581161906	3.2356856636025650
H	-3.6220979451793802	-1.5614778744187887	0.2180398875573459

Table S20 Equilibrium structure of the S_0 state of hexa-coordinated Chlorophyll a in isopropanol (ε_0=20.18, ε_∞=1.90), performed with the B3LYP functional.

Mg	1.3016914530038681	0.1457401030364174	0.4901487584021509
C	-2.0466564874745607	0.9717521082393766	0.3707185722362779
C	0.4536634531074044	-3.0551834593823379	1.3290153432038381
C	4.6003935164932441	-0.7202916342883665	0.3027832479261172
C	2.1006452322996729	3.3624600003663496	-0.6506136486809322
N	-0.5785207365776980	-0.8881002310663613	0.8224894438826637
C	-1.8377129019469645	-0.3473426973842998	0.7282870567014794
C	-2.8995307998895958	-1.3781474255598876	1.0568199051151901
C	-2.0827376355234164	-2.6509903959601360	1.3969711704413885
C	-0.6388602694370407	-2.1903805538788910	1.1772009009013287
C	-2.3425783398452782	-3.1831979792270930	2.8161262349378986
N	2.3547305429009282	-1.6102886298805914	0.7399436705492105
C	1.8321143268771722	-2.8048126103827098	1.1375817237880956
C	2.9063520874042048	-3.7755327452090923	1.3299308043775102
C	4.0889942288741761	-3.1174923628787639	1.0329280774848555
C	3.7214331285907889	-1.7416658518835693	0.6714784783292883
C	2.7285383923527924	-5.1884914698201863	1.7962899599025153
C	5.4642380733556797	-3.6032956807001271	1.0765891751775165
C	5.8917722986269796	-4.8814535720884269	1.0653682048920223
N	3.0504418429297533	1.1571578755517684	-0.0685502258559061
C	4.2998212049364781	0.6212965936940591	-0.0530478111958161
C	5.2818337398614048	1.6192701468120847	-0.4608265980846064
C	4.5770605964916111	2.7733662625137638	-0.7310251251686101
C	3.1701074454352511	2.4676718780216009	-0.4835090615618485
C	6.7623299119658249	1.3886784791773616	-0.5583740204358330
C	5.1151168996582852	4.0893376774461192	-1.2240785782725299
C	5.0851534922544692	4.2329393031242546	-2.7602358234417079
N	0.2921453727075745	1.8445923140733007	-0.0219150049914463
C	0.7295908045518736	3.0942797335052101	-0.4516624335691897
C	-0.3989542176495231	3.9744031898151753	-0.6676342697086262
C	-1.5226751392936499	3.1916559751906157	-0.3486477017645940
C	-1.0345145144117074	1.9090662237720553	0.0357504025659591
C	-0.3650093557754301	5.3973117365409493	-1.1318941101802864

C	-2.9648895041363135	3.1717093075372733	-0.2762718671730010
O	-3.7953508524684554	4.0508544808588844	-0.5094303035161888
C	-3.3756328576998591	1.7063905193032807	0.1876430767341092
C	-4.2777752135145066	1.8063205558359179	1.4077008300649378
O	-5.4877812710215794	1.6470329736564910	1.3781530048774657
O	-3.6006588697053354	2.1329717110523259	2.5187342617225603
C	-4.3747146794240237	2.3069841604077990	3.7305928279300193
H	0.1975144460296445	-4.0675870259457643	1.6233143329569535
H	5.6536728209455758	-0.9789924759016428	0.2726454822050184
H	2.3585007005248806	4.3625451298389430	-0.9847762962554670
H	-3.5169175784675879	-1.0551303740399660	1.9035007078719803
H	-2.3089794688909100	-3.4517620183329325	0.6824541708893670
H	-2.1194692346243738	-2.4151184739535125	3.5655326650348060
H	-1.7287007162305126	-4.0632664930846403	3.0335044462777492
H	-3.3939388126652146	-3.4707953728703242	2.9253689239427785
H	3.5042268898579114	-5.4602851799847256	2.5210938455768246
H	2.8017992853836033	-5.9044735692274752	0.9673461195651771
H	1.7569752340352800	-5.3382789938940345	2.2731516229861741
H	5.2223134209390736	-5.7315051324439761	0.9941301840004729
H	6.9548692967810748	-5.0999035101272812	1.1087426013493773
H	7.1716192499021023	0.9951089470607107	0.3798807637241824
H	7.2927595084773484	2.3162820127078709	-0.7889055518311925
H	7.0087087003317938	0.6629888449603755	-1.3436357181510650
H	4.5446450377640648	4.9128517074125719	-0.7784269539805101
H	6.1473909154560475	4.2115175809781649	-0.8772996549318960
H	4.0623874095582391	4.1530239566498484	-3.1432843516883842
H	5.6856737510576263	3.4510910192773263	-3.2376089404259956
H	5.4873438650386523	5.2057205082828482	-3.0646951171485863
H	0.6484329024987607	5.7201546998436541	-1.3824896107418343
H	-0.7523938098251700	6.0720475631931548	-0.3586297172047201
H	-0.9952378604707368	5.5347807959934183	-2.0184136456637183
H	-3.9908358530993202	1.2701949770843763	-0.6074393479354140
H	-3.6477701516314469	2.5635190048119241	4.4995486819201220
H	-4.8907776268612846	1.3783644757418192	3.9826412813340726
H	-5.0989603419096747	3.1135371150629578	3.5996161001384013
H	6.2294185902874224	-2.8296946676194019	1.1159250451260900
O	1.3656257227859758	0.6327611999731522	2.6365738388731521
C	2.5165247532733228	0.9265128776429983	3.4787121103704366
C	2.3324287972882303	0.2859385490268515	4.8513011935875081
C	2.7550963799966928	2.4319991435440107	3.5487910216109486
H	0.5877037939948371	1.0937028409820118	2.9864227315086076
H	3.2285069672638058	0.4377128719675935	5.4625279000315468
H	2.1558944589040472	-0.7898000409791301	4.7574909140134354
H	1.4813338729615773	0.7364326912658774	5.3764005446842189
H	3.6585385133388599	2.6461720805632707	4.1298763693613614
H	2.8808685032530250	2.8521600773270772	2.5468586739211618
H	1.9102808614596922	2.9334648377120676	4.0371893060806023
H	3.3464215650718363	0.4455599451744828	2.9534883479179479
O	1.1238732455035667	-0.8261955747566455	-1.8310062473658275
C	1.6021691643377647	-0.3992356942851916	-3.1322096906897365
C	1.7088795991279853	-1.5942317313806931	-4.0768936338969466
C	0.6392403531673448	0.6670740900121580	-3.6354873211491427
H	2.5982135012333676	0.0463668756392344	-2.9994909745556066
H	2.1067723336296309	-1.2762627490711425	-5.0467671952759119
H	0.7240693786804735	-2.0479243205862239	-4.2367353378273647
H	2.3829172203059867	-2.3573882644951154	-3.6703778015573065
H	-0.3602264397953688	0.2426926885598854	-3.7850872947111416

H	0.9911848920983687	1.0687316437085996	-4.5911535206274472
H	0.5668670015643757	1.4902500613308465	-2.9193695904379524
H	1.6771221668897516	-1.5642139681872094	-1.5298449630387043
H	-3.5765815764129081	-1.5288111951042820	0.2087600046877954

Table S21 Equilibrium structure of the S_0 state of tetra-coordinated Bacteriochlorophyll a in acetone (ε_0=21.01, ε_∞=1.85), performed with the B3LYP functional.

Mg	-0.4840410587533359	-0.0291961648519482	-0.4217987208677256
C	2.8777911229653577	0.5460662655867033	-1.0130472007336948
C	-1.0656928343563474	3.3186778556120546	-0.8006098317305799
C	-3.7713058648444804	-0.6251865870647493	0.1289546488367551
C	0.1807210758989194	-3.4458981310920174	-0.1733327065721875
N	0.7664869952497567	1.6935395242394367	-0.8392767954339071
C	2.1201329937764481	1.7007260252403023	-1.0637932454041901
C	2.6154613925171071	3.0927351860339352	-1.3985757547004640
C	1.3808998594728679	3.9850628516983253	-1.1298339380326856
C	0.2719590100358638	2.9543579108837101	-0.9187583591278721
C	1.5547140965799084	4.9030383098900510	0.0927796052773124
N	-2.1481162058327361	1.1495201024009147	-0.3640994041245580
C	-2.1907199312464338	2.5093856758600559	-0.5535893122702078
C	-3.5523130621722756	2.9808634432473324	-0.4376739635191550
C	-4.3357024840330487	1.8583281340278444	-0.1593939686518544
C	-3.4254307523924168	0.7151717038065384	-0.1246313576700407
C	-3.9570442857948822	4.4160249478290821	-0.6104106857285045
C	-5.7941077780420729	1.7845062589253773	0.0700902461001725
O	-6.3769906691890146	0.7035937821992944	0.1982018582055492
C	-6.6161936347715731	3.0578393361421186	0.1630696240360217
N	-1.6193162442031164	-1.7801393262102700	-0.0663847059809081
C	-2.9546385552685339	-1.7552553470627681	0.1574453135358139
C	-3.4954525535397969	-3.1444924271332142	0.4747418848059836
C	-2.2830736015954360	-4.0629619895386808	0.1683535156541285
C	-1.1446782319328437	-3.0659731897435019	-0.0264266394401548
C	-3.9845538227511486	-3.2572640363311218	1.9294059038197959
C	-2.4807178262161140	-4.9986132834712818	-1.0480963713168512
C	-2.7125623687575531	-4.2945237478731952	-2.3898715110439901
N	1.1440240358152611	-1.2250599766388703	-0.5322581625732959
C	1.2889351260928039	-2.6018938318509193	-0.4149221881550888
C	2.6685336135987208	-2.9828646564623442	-0.5935746514559803
C	3.3438617185982111	-1.7775027472151783	-0.8220368317584820
C	2.3728394542441356	-0.7450092813152120	-0.7733018369865503
C	3.2294210814258908	-4.3714685439680574	-0.5484183096193985
C	4.6335256395630671	-1.1773951558019102	-1.1088070852156251
O	5.7407693391151051	-1.6856240451406483	-1.2384604714747969
C	4.3802765351241630	0.3823907999710516	-1.2446502721165169
C	5.2599612075817745	1.1415315597167004	-0.2608331498488348
O	6.1102602282258465	1.9525122021141421	-0.5745615569485738
O	4.9893326645722684	0.7956669529673722	1.0090403257887428
C	5.7746214496708124	1.4503923384932769	2.0289645747991156
H	-1.2549498796596270	4.3811284102593895	-0.9081673840899982
H	-4.8231133540690747	-0.7964977376556770	0.3160339756263879
H	0.3853781615044709	-4.5110980363038617	-0.1063825046771771
H	3.4828112490734351	3.3827209561850484	-0.7955557786325586
H	1.1484766077980544	4.6077344710713604	-2.0010962723897299
H	1.8019723441170152	4.3190021752240382	0.9873687243697484
H	0.6401560511981498	5.4701482974147160	0.2968187105464207

H	2.3674145015654720	5.6164598492910072	-0.0841538070920797
H	-4.7734479983668816	4.5232948389870904	-1.3313194125658392
H	-3.1260776276656479	5.0247001653776922	-0.9721523287873388
H	-4.2989676875891947	4.8586947998201948	0.3328549459577574
H	-6.2012419611775798	3.7585613998892433	0.8944558734468701
H	-7.6336168637911923	2.7862807284191224	0.4527479800480239
H	-6.6490510727573522	3.5734795385222249	-0.8031577041861895
H	-4.3389667203705082	-3.3742260212445623	-0.1880897797512897
H	-2.0671228868537348	-4.7016385708230395	1.0342292238019481
H	-3.1727473398893205	-3.0392486811493962	2.6337393508136349
H	-4.3436486956424698	-4.2735766355165330	2.1288593081629621
H	-4.8054637356920828	-2.5594914839293619	2.1277901833762933
H	-1.5997830616093960	-5.6483378800484676	-1.1314535684235969
H	-3.3304458039928257	-5.6585917064793918	-0.8265817851765095
H	-1.8617043119176833	-3.6567051129595729	-2.6570241354535558
H	-3.6125895139866540	-3.6687732256313801	-2.3759593052099608
H	-2.8383287256433176	-5.0324412945719077	-3.1902188686194144
H	2.9970800810126197	-4.8693517638683748	0.4012297193482874
H	4.3163025322572546	-4.3505062183129981	-0.6650033068220058
H	2.8151813088855850	-4.9999350369248141	-1.3475135566021064
H	4.6917188322635557	0.7014869875542151	-2.2453444248414418
H	5.4134558087773312	1.0481218548565743	2.9748639908486516
H	5.6223615502146407	2.5314609436363562	1.9875369423311362
H	6.8346745707025995	1.2229855926540436	1.8938638091609390
H	2.9326385069860184	3.1357553863295933	-2.4483320851520998

Table S22 Equilibrium structure of the S_0 state of penta-coordinated Bacteriochlorophyll a in acetone (ε_0=21.01, ε_∞=1.85), performed with the B3LYP functional.

Mg	0.8892083791335895	0.3724992044008220	0.4215894740031140
C	-2.5831881723151238	0.4871208443514161	0.1565579120310360
C	0.6116498852244610	-3.0081627969479392	1.0571501257474909
C	4.2323717901107907	0.0036016688656280	-0.2032600145419914
C	1.0228566252606548	3.5166489272220995	-1.1847347723267689
N	-0.7871344554066968	-1.0456628138877295	0.6326244955895431
C	-2.1263602179695686	-0.7574016173026751	0.5435484180878790
C	-2.9795288166707619	-1.9664897359496059	0.8681908600414978
C	-1.9414167976565349	-3.0465295384616904	1.2584639927979793
C	-0.6129382969875876	-2.3431575468617254	0.9755579447547680
C	-2.0718321443140622	-3.5115313424586043	2.7196254475381383
N	2.1986965944849985	-1.2407877549873385	0.3912935773722515
C	1.9046884650140794	-2.5250718392561398	0.7760435139482988
C	3.1192325610706813	-3.3156800832394286	0.8442827023949320
C	4.1574781728535708	-2.4639460178506765	0.4717511705600596
C	3.5489639188271758	-1.1625034024725460	0.1853105654786830
C	3.1677392164551925	-4.7708055254779698	1.2095859716595603
C	5.5949446418020976	-2.7752816709675354	0.3571842144489190
O	6.3491340922020596	-2.0926734787244827	-0.3504810018422973
C	6.1792773409511517	-3.9436117024319910	1.1270561958666840
N	2.4031283984130152	1.5974854525329187	-0.5283365271443391
C	3.7107571206473371	1.2577143104266595	-0.5387800004588075
C	4.5966399752780989	2.4173720175785380	-0.9780550956507305
C	3.5652871230188170	3.4554884318970824	-1.4914123462838886
C	2.2342959583676270	2.8595474152305655	-1.0375744319943072
C	5.4710197885302323	2.9502582379959046	0.1722464087081303
C	3.6326955467460764	3.7228375674520855	-3.0149311614567580

C	3.3498198750519887	2.5090744791020323	-3.9101883253792478
N	-0.4447567388126933	1.7467707340340175	-0.3329537251307503
C	-0.2686821556965319	3.0104387791874294	-0.8887434525979792
C	-1.5380550794786183	3.6403645369175059	-1.1447368822675856
C	-2.4926192406325010	2.6938651484902532	-0.7279244204514190
C	-1.7696065506520946	1.5679605218878570	-0.2485874369149327
C	-1.7730237091610277	4.9851404367727081	-1.7626372230640155
C	-3.9029542951297573	2.4047243521002239	-0.6421886960879744
O	-4.8857217618729614	3.0862069766206237	-0.9412908958016281
C	-4.0279056203557762	0.9314260340314642	-0.0621676344141379
C	-4.9392087646029283	0.9404044584029343	1.1542979319289137
O	-6.0961355979181775	0.5506582512326216	1.1417707133786950
O	-4.3471955687815615	1.4671941903834609	2.2365570171914517
C	-5.1507516769194233	1.5706522537154448	3.4371871963576348
H	0.5437328587446809	-4.0505741922406555	1.3483668445572796
H	5.3059091115433032	-0.0991668470206645	-0.2977875029020495
H	1.0702303952742844	4.5121694144389091	-1.6154176311502160
H	-3.6796411368371298	-1.7570314958850830	1.6852714323914793
H	-2.0307933716601538	-3.9235839023436019	0.6071190488754538
H	-1.9752917301493811	-2.6633500089109647	3.4071908722972895
H	-1.3040437224885897	-4.2488102513155477	2.9749413893427672
H	-3.0525981259932156	-3.9719582411111318	2.8815734545713552
H	3.7977914902977354	-5.3393128172495432	0.5201481624244021
H	2.1736427204061872	-5.2205297766828753	1.1911620790930444
H	3.5751458192794172	-4.9178988370975505	2.2172582994682215
H	5.7426966691567385	-4.0479757700695291	2.1231952292444580
H	7.2594625306205005	-3.8031653394735274	1.2040505892485136
H	5.9939090689020720	-4.8772622583926619	0.5831325606280597
H	5.2618567590415113	2.0919830904845753	-1.7866415686696455
H	3.7208962118745639	4.4190418721411673	-0.9897288803022568
H	4.8521881936537667	3.2802415939552327	1.0145725864613735
H	6.0628814826530233	3.8059795765660387	-0.1714404231205112
H	6.1621505295760928	2.1823707487442556	0.5353077269877418
H	2.9206681822683920	4.5214869055948794	-3.2583245743734168
H	4.6309023275638248	4.1213774464477373	-3.2390668455410161
H	2.3455072136206234	2.1092942372194687	-3.7306564969228662
H	4.0693711250406999	1.6996539632073770	-3.7440903703639590
H	3.4117617847207837	2.7918074036187650	-4.9665887649439258
H	-0.8919257671609635	5.6275781592038259	-1.6842237473209944
H	-2.6123575874614251	5.4961617467296993	-1.2801221387509638
H	-2.0218730254552981	4.8945914413680782	-2.8284338170843504
H	-4.5403042342247799	0.3226863046699127	-0.8157230697870068
H	-4.4936986875399949	2.0141371473176113	4.1836072540738440
H	-5.4801167419805124	0.5791700833605002	3.7543238639145855
H	-6.0157997947854858	2.2116871555587356	3.2553651248428421
O	1.2831861573793275	1.0144596125695429	2.3749278399643838
C	0.8288581805669256	1.3609844262859392	3.4721047269483454
C	1.7590112172909171	1.7743369699038438	4.5750620166028142
C	-0.6455206075705486	1.3892058769202651	3.7485340882316689
H	1.4112150927802274	2.7046747866793859	5.0360410286342079
H	2.7787977256773781	1.8859628387622882	4.2046248453811739
H	1.7353996777885017	1.0048396555590853	5.3575040722084335
H	-0.8568399583831704	0.9250219325347969	4.7174992181766520
H	-1.2126946857351282	0.9000838250753035	2.9567045915365768
H	-0.9592949611150670	2.4382610049836848	3.8265667962310750
H	-3.5805881892898155	-2.2609378456621139	0.0001782818190042

Table S23 Equilibrium structure of the S_1 state of penta-coordinated Bacteriochlorophyll a in acetone (ε_0=21.01, ε_∞=1.85), performed with the B3LYP functional.

Mg	0.8938259587	0.3762083249	0.441931239
C	-2.5881007756	0.491860802	0.1690146391
C	0.6224494593	-3.005772583	1.0651737015
C	4.2492978343	0.0207939385	-0.1770510232
C	1.0111044249	3.5134613564	-1.1983246364
N	-0.7714074114	-1.0278735775	0.6491441128
C	-2.1183350754	-0.7481354016	0.559011231
C	-2.9638296852	-1.9533009899	0.8947880356
C	-1.9264604702	-3.0429660507	1.2444901493
C	-0.5999125384	-2.3353724291	0.9819617256
C	-2.0588113134	-3.5692888365	2.6856961068
N	2.2172557024	-1.2321900817	0.4148371982
C	1.9226376963	-2.5249522982	0.7937260658
C	3.1319872719	-3.3156235388	0.862050859
C	4.1820584032	-2.4579302711	0.5054147448
C	3.5655833611	-1.1526994295	0.2189507832
C	3.1672209411	-4.7736202856	1.2147227442
C	5.6160251555	-2.7512097553	0.4201735054
O	6.412607962	-1.9769747855	-0.1457008271
C	6.1747084899	-4.0178734502	1.0440284809
N	2.3942105767	1.597300366	-0.5076581593
C	3.7156573456	1.2634019964	-0.5181204734
C	4.5877001052	2.4242017953	-0.9701891067
C	3.550802597	3.4476312245	-1.4928356643
C	2.2274117217	2.8492754198	-1.0380435213
C	5.4651567639	2.9792872257	0.1697831749
C	3.6224493413	3.7096980228	-3.0197255189
C	3.3468089045	2.4913168431	-3.9104431187
N	-0.4474210992	1.7467308725	-0.3274580088
C	-0.2729619295	3.0058232612	-0.8977931588
C	-1.5532490641	3.6362888246	-1.1604961302
C	-2.5029019469	2.694833086	-0.7366612409
C	-1.7716148373	1.56890685	-0.2445193432
C	-1.7790410184	4.9727099606	-1.7933306605
C	-3.9122861751	2.4010310934	-0.6498580158
O	-4.8986444663	3.0816250228	-0.9543999063
C	-4.0312195279	0.9341962737	-0.0550865354
C	-4.9418096249	0.9553429267	1.1616909026
O	-6.1003029638	0.5696468462	1.1516651438
O	-4.3497654464	1.490535781	2.2403532691
C	-5.1552672697	1.6075199122	3.4381974567
H	0.547792604	-4.0493217282	1.3494893484
H	5.3226533168	-0.0780851708	-0.2662159283
H	1.0612302398	4.5027223226	-1.6417820392
H	-3.6383120183	-1.742096435	1.7335492844
H	-2.0156416077	-3.8943228547	0.5585488771
H	-1.9543140329	-2.7512260144	3.4073315737
H	-1.2975427239	-4.3232784318	2.9073699401
H	-3.0434881075	-4.0277582509	2.8273121291
H	3.7811527826	-5.346485546	0.5136290876
H	2.166941622	-5.2100752754	1.2035548752
H	3.5837708816	-4.941322303	2.2164010346
H	5.749874393	-4.2192499012	2.0305237608
H	7.2585733502	-3.9118377247	1.1246379487

H	5.9607205121	-4.8841431771	0.4073721735
H	5.2536861589	2.091534449	-1.77498073
H	3.6911001384	4.4175706431	-0.9980820707
H	4.8484398066	3.3137403855	1.0115780803
H	6.0459152783	3.8354883609	-0.190291046
H	6.1646607686	2.2211648164	0.5356970752
H	2.9100673732	4.5057655587	-3.2679608345
H	4.6209457059	4.1095329075	-3.2382459839
H	2.3389441356	2.095623679	-3.7422340461
H	4.0615123449	1.6804045717	-3.7314605082
H	3.4234328464	2.7691316082	-4.9671175285
H	-0.9208080473	5.6369669501	-1.6572363069
H	-2.6634298985	5.4611802384	-1.3719448793
H	-1.950385417	4.8778591577	-2.8753064041
H	-4.5426194763	0.3163939961	-0.8020553852
H	-4.4975282949	2.053526048	4.1825442319
H	-5.4905900176	0.6203928463	3.7627262385
H	-6.0166885827	2.2515700852	3.2497155179
O	1.2812129621	1.0243804825	2.3955586469
C	0.8232456487	1.3766548895	3.489321345
C	1.7500511831	1.7872782276	4.5962199971
C	-0.6524622202	1.4146197357	3.758058426
H	1.4037815161	2.7190306507	5.055432137
H	2.7718325396	1.8949056305	4.2300859467
H	1.7204465712	1.0179849177	5.3786789526
H	-0.8713379194	0.9556786111	4.7278359063
H	-1.2182389924	0.9253226166	2.9653923953
H	-0.9604664517	2.4658032858	3.8298615732
H	-3.5970882433	-2.2330191207	0.0443569664

Table S24 Equilibrium structure of the S_2 state of penta-coordinated Bacteriochlorophyll a in acetone (ε_0=21.01, ε_∞=1.85), performed with the B3LYP functional.

Mg	0.8841784033	0.3859758502	0.4320762103
C	-2.5920102085	0.4901605676	0.1381718793
C	0.6116421946	-2.9805266509	1.1152231747
C	4.2379513599	0.0344934713	-0.1473740624
C	1.0126062131	3.5213125314	-1.1894651159
N	-0.7928498845	-1.024802228	0.6527945286
C	-2.1254891442	-0.7546283678	0.5447598983
C	-2.9758730762	-1.9655547526	0.8653886918
C	-1.9380988823	-3.0238276493	1.3129861632
C	-0.6098592273	-2.3244654044	1.0204376062
C	-2.0849263348	-3.4256020506	2.7916894903
N	2.2194584676	-1.2291269472	0.44760428
C	1.9309748964	-2.5179912946	0.8430725218
C	3.1271250859	-3.3013783737	0.9234065384
C	4.1883052441	-2.4386915867	0.5565931929
C	3.5730154914	-1.1480690682	0.2660951327
C	3.1656930941	-4.7502309026	1.3167135164
C	5.6170781179	-2.7286090991	0.4588410714
O	6.4436051935	-1.8647698681	0.0955645862
C	6.1463231323	-4.1096954735	0.8034746108
N	2.3970332983	1.6095246772	-0.5142997412
C	3.7093006075	1.2797140486	-0.5054586919
C	4.5910560812	2.4407143048	-0.9478419392

C	3.5570528693	3.4686810496	-1.4766530248
C	2.2270227776	2.8665187043	-1.0307106834
C	5.457354008	2.9891979664	0.2012577164
C	3.6360774121	3.7278922316	-3.0011520487
C	3.3730714437	2.5065997462	-3.8922625743
N	-0.4616998985	1.7672488703	-0.3287080722
C	-0.2923417322	3.0264920049	-0.8995935662
C	-1.5546383784	3.6484629099	-1.1761827248
C	-2.5184107608	2.6959455818	-0.7619227975
C	-1.7883657987	1.5822664246	-0.2620656179
C	-1.7833263326	4.9858563882	-1.8123180758
C	-3.9179851923	2.3896404668	-0.7053721307
O	-4.9154822864	3.0524582631	-1.0294143801
C	-4.0302225837	0.9166201527	-0.1173786601
C	-4.9693812902	0.9163500593	1.0773430316
O	-6.1252344138	0.5270322592	1.0349479598
O	-4.4036657552	1.4418765564	2.1745137381
C	-5.2382970396	1.5471369728	3.3539172143
H	0.5346923549	-4.0201509693	1.4162028008
H	5.3138333077	-0.0599756305	-0.2219327426
H	1.0661998094	4.512813366	-1.6296045024
H	-3.7083331689	-1.7482487745	1.6510087257
H	-2.0167847746	-3.9284907646	0.69982145
H	-2.0083169841	-2.5476427405	3.4431166374
H	-1.311489223	-4.1412864009	3.0886070199
H	-3.0620926749	-3.8917106691	2.9595860796
H	3.5665817114	-5.3809098702	0.515167952
H	2.1685705088	-5.1258763529	1.5525424256
H	3.7916122735	-4.9181858926	2.1997663196
H	5.919050344	-4.3784926789	1.8400753799
H	7.2290859815	-4.1078778434	0.6645811788
H	5.7054625313	-4.8791466061	0.1621340262
H	5.2615934632	2.1114463446	-1.7503410287
H	3.7005813842	4.4359768412	-0.9787503696
H	4.8335195525	3.321827785	1.0387503984
H	6.0442246976	3.8458385495	-0.1485953322
H	6.1527282986	2.2292757884	0.5726770761
H	2.920086065	4.519262063	-3.2561978588
H	4.6330467504	4.1338919726	-3.2167033117
H	2.3693081251	2.1004840653	-3.7238405396
H	4.0962293991	1.7033611491	-3.7124223136
H	3.4465438216	2.7839828718	-4.9492782447
H	-0.9146742982	5.6415795432	-1.7019000255
H	-2.6462853594	5.4900553901	-1.3651225582
H	-1.9885458216	4.8925612046	-2.8879188747
H	-4.5079867551	0.2877739255	-0.8777796578
H	-4.597653204	1.9822813643	4.1192527079
H	-5.5844043306	0.5573091811	3.6579919181
H	-6.0925141621	2.1964852104	3.1516172344
O	1.2865469655	1.0472115555	2.3641782275
C	0.8748598671	1.3498957477	3.4909580907
C	1.8362901841	1.8224350952	4.5412793422
C	-0.5788069763	1.2703823521	3.8524729791
H	1.4594417529	2.7361779162	5.0132191097
H	2.8278131891	1.9888581334	4.1186620429
H	1.8937208677	1.0606856631	5.3291169587
H	-0.7013593557	0.8326340156	4.848167108

H	-1.1451839236	0.7067942283	3.1112697741
H	-0.9713098534	2.2944391293	3.9060394737
H	-3.5389895063	-2.284077601	-0.0195939246

Table S25 Equilibrium structure of the S_0 state of hexa-coordinated Bacteriochlorophyll a in acetone (ε_0=21.01, ε_∞=1.85), performed with the B3LYP functional.

Mg	-0.7079370529354372	0.3011415770182183	-0.3086237150697099
C	2.7419865437567923	0.2209810400572333	-0.4150308773683744
C	-0.7648616964459752	-3.0803258834721445	-0.7863747486720922
C	-4.0952310046520193	0.4301865011493793	-0.1983937241170201
C	-0.5703731784098477	3.7541247690483810	0.2986205288389626
N	0.8211776890999717	-1.2244313718854249	-0.5790962414584199
C	2.1752944488498276	-1.0345478123856997	-0.5736615933411083
C	2.9134987538920796	-2.3512073714513342	-0.7322013451299226
C	1.7773945459939955	-3.3716345686845641	-0.9946483525403783
C	0.5177042955983504	-2.5294367889073652	-0.7694589008528537
C	1.8244664972836373	-3.9823474054837282	-2.4054945443029752
N	-2.2048485786972241	-1.1075102784859174	-0.4667571579062401
C	-2.0226485565173391	-2.4508726630356077	-0.6418796422903267
C	-3.3090406568779454	-3.1193145942519358	-0.6677359468691995
C	-4.2736201517218522	-2.1204393836719437	-0.5054938182303340
C	-3.5405176538964178	-0.8561577104002285	-0.3760129239266972
C	-3.4813734649784682	-4.6020108816164687	-0.8268837944802276
C	-5.7416472095518847	-2.2541911335741651	-0.4645049515605505
O	-6.4775102015752442	-1.2908631337425862	-0.2230741409824753
C	-6.3952478852752481	-3.6003635968294221	-0.7289948025206907
N	-2.1247238511836382	1.8773843333059066	0.0051733681189912
C	-3.4577395058910145	1.6651971650522575	-0.0370259449047768
C	-4.2342444722285100	2.9729554484038623	0.0989100310429087
C	-3.1234482794150118	3.9964011380948756	0.4596643130949369
C	-1.8414326628771174	3.1934101071519008	0.2393565777128487
C	-4.9870393501957038	3.3347037626008982	-1.1935984196401215
C	-3.2382362795718835	4.5900531028712281	1.8836459832348447
C	-3.1181942221480603	3.5798860690886447	3.0297341165859226
N	0.7378749451151085	1.7357355244535251	-0.1340071075733731
C	0.6725886574757526	3.0917916530279914	0.1335683777232191
C	2.0015887138612456	3.6526026286351074	0.2220807742792026
C	2.8606787194273764	2.5674033962488969	-0.0025162979452641
C	2.0357253054040521	1.4285491828287080	-0.2131443468390524
C	2.3603275418423650	5.0820941831958173	0.4939617783733121
C	4.2451340882705662	2.1476297745814485	-0.0720744247428062
O	5.2856938078389497	2.7858619423991842	0.0513374111022862
C	4.2265518049028525	0.5861819741781797	-0.3571680433400746
C	5.0100823630626170	0.2830514982387440	-1.6258131282885084
O	6.0025619718439875	-0.4181147251388634	-1.6775795233752424
O	4.4798812110860817	0.9021426553452747	-2.6961254543680204
C	5.1634842689580429	0.6989963486938592	-3.9512773141300133
H	-0.7878654538542672	-4.1546650164760797	-0.9376275890198414
H	-5.1771336611733210	0.4549552429522974	-0.1750387728572581
H	-0.5280918451656513	4.8222031080356764	0.4978856239057113
H	3.6417287129731255	-2.3180122636206049	-1.5508485197244362
H	1.8102213833367962	-4.1877434505435023	-0.2630569108391443
H	1.7810566535198775	-3.1972402135664675	-3.1703548407153948
H	0.9843739630389134	-4.6658663327314880	-2.5704761680128003
H	2.7535894904154663	-4.5462295053767265	-2.5481766803777255

H	-4.0690413774043108	-5.0322106146364423	-0.0088063049327280
H	-2.5190691754992236	-5.1176623390053484	-0.8401134781231332
H	-3.9987439435443726	-4.8550534489148482	-1.7600005057773516
H	-6.0778872527156667	-4.0248558290890575	-1.6869330276528340
H	-7.4779579630412369	-3.4568998693332236	-0.7358269713421445
H	-6.1383901225822335	-4.3251920222637397	0.0516310274084144
H	-4.9692513273061332	2.8877602705252339	0.9091744719428131
H	-3.1491614600541413	4.8397240317504213	-0.2424714626195134
H	-4.2937712643247599	3.4211469968867405	-2.0391342735164617
H	-5.5031010777948648	4.2952947908503889	-1.0785894191951970
H	-5.7348563033181410	2.5741641970622684	-1.4438241420705260
H	-2.4634791579563506	5.3595393333309680	1.9995358746991616
H	-4.2022009181964144	5.1126440140451095	1.9526527454245839
H	-2.1446691956893065	3.0763332694036905	3.0134798143273245
H	-3.8961310491376788	2.8086767007974185	2.9809560621546032
H	-3.2159798294102693	4.0847476190784366	3.9975588205725878
H	2.0172098563649588	5.7457186994917997	-0.3109897632980678
H	3.4443125245746322	5.1957162860679054	0.5828152269711987
H	1.9014013341486311	5.4461702845199182	1.4217766198272694
H	4.7575173669317099	0.0780377591446924	0.4556129194349632
H	4.5993244500782975	1.2738599050335653	-4.6849805548579173
H	5.1667027150014739	-0.3614412817702280	-4.2146011178755343
H	6.1913352383527140	1.0634974241825954	-3.8848020534776553
O	-0.9250211189200099	0.6539593579780343	-2.5266080531871693
C	-0.4741273262323850	0.6503134610387955	-3.6710354936617366
C	-1.3450298517152457	1.0245429941683928	-4.8411450953069037
C	0.9504062202746704	0.2553625730915909	-3.9642399782937932
H	-0.9678270580140411	1.9555252335359370	-5.2838376793862727
H	-2.3822482842922823	1.1601624718112920	-4.5284449324671971
H	-1.2811492086663867	0.2564951685025030	-5.6214063198102817
H	1.3375731499988124	0.7843606303661229	-4.8399060649402994
H	0.9623149420073506	-0.8185048326582599	-4.1978978265812623
H	1.5915555732710003	0.4261463536265639	-3.0977951591569641
O	-0.7674867941676108	-0.0518498883362272	1.9448188491840432
C	-0.1914391601401267	-0.0295886816461524	3.0313758402213749
C	-0.9593352381907522	-0.2226489391888103	4.3127101256020675
C	1.2929609651221905	0.2084430952864456	3.1435716693967639
H	-2.0338488232985723	-0.2573416523532592	4.1227597597576660
H	-0.7232511953447038	0.5828299664029190	5.0183477867807129
H	-0.6366199199693283	-1.1597905862082465	4.7842312456684661
H	1.4522835137876753	1.2844005777419498	3.2991359332249681
H	1.7159984655426737	-0.3139427378569504	4.0065028675267289
H	1.8052498016885050	-0.0846601006742881	2.2257476717650584
H	3.4762997741723938	-2.5862286821118579	0.1796661619405177

S9. Comparison of total energies and excitation energies between wB97X-D-optimized and B3LYP-optimized structures

All energy calculations were performed with [TD]DFT using the 6-31++G(d,p) basis set and the C-PCM. Either the range-separated hybrid functional ωB97XD (in the following ''RSH" abbreviated) or the SRSH-PCM approach with the ωPBE functional (in short "SRSH") were used. Tuning parameters (α, β, γ) were determined for penta-coordinated Bchl a in ωB97X-D structures (0.266, -0.218, 0.129) and in B3LYP

structure (0.256, -0.208, 0.122) and for penta-coordinated Chl a in ωB97X-D structures (0.269, -0.219, 0.128) and in B3LYP structure (0.251, -0.201, 0.120).

Table S26 Excitation energies (in eV) based on B3LYP-structures (for experimental references, see main manuscript). The corresponding values based on ωB97X-D structures are listed in Table 1 of the main manuscript. Using B3LYP structures, all excitation energy calculations (RSH and SRSH) show seemingly better agreement with experimental values (see discussion in Table S28). In particular, the Bchl a S_1 excitation energies dropped by approximately 0.5eV correcting the erratic S_1-S_2 energy gap behavior seen with ωB97X-D-based structures. Bchl a RSH-excitation energies are in perfect agreement with experiments. Chl a RSH-energies are improved compared to ωB97X-D-based structures but are still overestimated and show a largely increased S_1-S_2 energy gap. SRSH-excitation energies are generally lower, correcting for the overestimation reported for ωB97X-D structures in the main manuscript. SRSH shows consistently good agreement with experiments and particularly in the predicted S_1-S_2 energy gap.

**B3LYP-structures**		RSH ωB97X-D			SRSH-PCM ωPBE			Experiment	
coordination		4	5	6	4	5	6	5	6
Chl a	S_1	2.01	2.00	1.99	2.06	2.06	2.05	1.86	1.85
	S_2	2.43	2.38	2.32	2.25	2.21	2.15	2.00	1.93
	ΔE	0.42	0.38	0.33	0.19	0.15	0.10	0.14	0.08
Bchl a	S_1	1.55	1.61	1.57	1.73	1.75	1.75	1.61	1.61
	S_2	2.14	2.12	2.02	2.18	2.14	2.04	2.15	2.03
	ΔE	0.59	0.51	0.45	0.45	0.39	0.29	0.53	0.42

Table S27 Total SCF ground state energies (in Hartree). Expectedly, using the RSH functional ωB97X-D, the ωB97X-D - optimized structures are more stable than B3LYP-optimized structures. Yet, it is noteworthy that the latter yield better agreement with experimental values in terms of excitation energies (see previous Table). Within the SRSH approach, B3LYP-optimized structures were found to be more stable in the case of Chl a, whereas for Bchl a ωB97X-D-structures are more stable. These findings indicate that mixing functionals for optimization and energy calculation might be problematic. For consistency, we decided to report only ωB97X-D-structures in the main manuscript, despite slightly better experimental agreement (Table S26) when using B3LYP-based structures.

Functional for energy calculation		RSH ωB97X-D		SRSH-PCM ωPBE	
	Coordination	ωB97X-D-based structure	B3LYP-based structure	ωB97X-D-based structure	B3LYP-based structure
Chl a	4	-1881,9654	-1881,9636	-1880,4630	-1880,4581
	5	-2076,3254	-2076,3189	-2074,6339	-2074,6289
	6	-2270,6716	-2270,6635	-2268,7896	-2268,7843
Bchl a	4	-1958,4022	-1958,3969	-1956,8296	-1956,8311
	5	-2151,5509	-2151,5442	-2149,7965	-2149,8006
	6	-2344,6887	-2344,6795	-2342,7508	-2342,7546

Table S28 Energy differences (in eV) between the ground state energy at the S_0 equilibrium geometry and the S_n (n=1-2) total energy at the respective excited state optimized geometry of penta-coordinated compounds. The minimum-to-minimum energy difference corresponds approximately to the fundamental line originating from a transition between the lowest vibrational state of the origin and the target electronic state. Interestingly, despite large differences in the structures and in the vertical excitation energies, the minimum-to-minimum energy differences are only marginally affected by the optimization protocol employed and are in good agreement with the experiment.
We therefore interpret the overestimation of vertical excitation energies observed for ωB97X-D-based structures (calculated with either approach RSH and SRSH) as a consequence of an overestimation in the coordinate shift between the equilibrium structures of ground and excited states. This can result in an overestimated reorganization energy and thus increased Huang-Rhys factors.

		RSH ωB97X-D		SRSH-PCM ωPBE	
	Transition	ωB97X-D-based structure	B3LYP-based structure	ωB97X-D-based structure	B3LYP-based structure
Chl a	$S_0 \rightarrow S_1$	1.96	1.97	2.00	2.02
	$S_0 \rightarrow S_2$	2.34	2.34	2.19	2.17
	ΔE	0.38	0.37	0.19	0.15
Bchl a	$S_0 \rightarrow S_1$	1.58	1.57	1.64	1.73
	$S_0 \rightarrow S_2$	2.14	2.12	2.04	2.09
	ΔE	0.56	0.55	0.40	0.46

Table S29 Relative angles between the $S_0 \rightarrow S_1$ and the $S_0 \rightarrow S_2$ transition dipole moments.

		RSH ωB97X-D			SRSH-PCM ωPBE		
coordination		4	5	6	4	5	6
ωB97X-D structure	Bchl a	78.4°	74.9°	70.2°	77.7°	77.6°	78.8°
	Chl a	53.8°	53.3°	54.4°	79.7°	75.0°	73.8°
B3LYP-sructure	Bchl a	79.3°	77.9°	79.1°	76.0°	74.6°	75.1°
	Chl a	57.9°	57.4°	57.4°	73.9°	71.3°	67.6°

References:

1. S. A. Kovalenko, A. L. Dobryakov, J. Ruthmann and N. P. Ernsting, *Phys Rev A*, 1999, **59**, 2369-2384.
2. Y. J. Shiu, Y. Shi, M. Hayashi, C. Su, K. L. Han and S. H. Lin, *Chemical Physics Letters*, 2003, **378**, 202-210.
3. P. Martinsson, J. a. I. Oksanen, M. Hilgendorff, P. H. Hynninen, V. Sundström and E. Åkesson, *Chemical Physics Letters*, 1999, **309**, 386-394.
4. T. A. Evans and J. J. Katz, *BBA - Bioenergetics*, 1975, **396**, 414-426.
5. D. Leupold, S. Mory, R. Konig, P. Hoffmann and B. Hieke, *Chemical Physics Letters*, 1977, **45**, 567-571.

6. H. Aksu, A. Schubert, E. Geva and B. D. Dunietz, *Submitted*, 2019.
7. S. Bhandari, M. S. Cheung, E. Geva, L. Kronik and B. D. Dunietz, *J Chem Theory Comput*, 2018, **14**, 6287-6294.
